# Circulating Non-Coding RNAs as Indicators of Fibrosis and Heart Failure Severity

**DOI:** 10.3390/cells14070553

**Published:** 2025-04-07

**Authors:** Veronika Boichenko, Victoria Maria Noakes, Benedict Reilly-O’Donnell, Giovanni Battista Luciani, Costanza Emanueli, Fabio Martelli, Julia Gorelik

**Affiliations:** 1 National Heart and Lung Institute, Imperial College London, Hammersmith Campus, Du Cane Road, London W12 0NN, UK; 2 Department of Surgery, Dentistry, Pediatrics and Gynecology, Cardiovascular and Surgical Sciences, The University of Verona, Policlinico G. B. Rossi, P.le. La Scuro 10, 37134 Verona, Italy; 3 Molecular Cardiology Laboratory, IRCCS Policlinico San Donato, Via Morandi 30, San Donato Milanese, 20097 Milano, Italy

**Keywords:** heart failure, cardiac fibrosis, ncRNAs, microRNAs, long non-coding RNAs, circular RNAs, HFpEF, HFrEF, biomarkers

## Abstract

Heart failure (HF) is a leading cause of morbidity and mortality worldwide, representing a complex clinical syndrome in which the heart’s ability to pump blood efficiently is impaired. HF can be subclassified into heart failure with reduced ejection fraction (HFrEF) and heart failure with preserved ejection fraction (HFpEF), each with distinct pathophysiological mechanisms and varying levels of severity. The progression of HF is significantly driven by cardiac fibrosis, a pathological process in which the extracellular matrix undergoes abnormal and uncontrolled remodelling. Cardiac fibrosis is characterized by excessive matrix protein deposition and the activation of myofibroblasts, increasing the stiffness of the heart, thus disrupting its normal structure and function and promoting lethal arrythmia. MicroRNAs, long non-coding RNAs, and circular RNAs, collectively known as non-coding RNAs (ncRNAs), have recently gained significant attention due to a growing body of evidence suggesting their involvement in cardiac remodelling such as fibrosis. ncRNAs can be found in the peripheral blood, indicating their potential as biomarkers for assessing HF severity. In this review, we critically examine recent advancements and findings related to the use of ncRNAs as biomarkers of HF and discuss their implication in fibrosis development.

## 1. Introduction

### 1.1. Heart Failure

Heart failure (HF) is a cardiovascular disorder affecting approximately 56 million individuals worldwide [1]. In HF, cardiac function is compromised, leading to the heart being unable to pump blood efficiently around the body. Affected individuals experience symptoms of dyspnoea due to circulatory hypoxia, and swelling of the limbs as a result of the abnormal gathering of bodily fluids [1,2]. Due to the strain placed on the heart, HF patients often find it difficult to complete basic daily tasks. As the condition progresses, individuals are susceptible to liver and kidney damage and an increased risk of sudden cardiac arrest [3,4]. HF is a complex and heterogeneous disorder with diverse aetiologies, often arising due to underlying cardiovascular diseases (CVDs), such as coronary artery disease (CAD), myocardial infarction (MI), cardiomyopathies, including dilated cardiomyopathy (DCM), hypertrophic cardiomyopathy (HCM), diabetic cardiomyopathy, and many more [1,5]. CVDs continue to be the leading cause of death globally, highlighting the plight and severity of HF.

A key feature of the pathology of HF is cardiac fibrosis, characterized by the scarring of the myocardium and the excessive deposition of the extracellular matrix (ECM) [6]. Fibrosis can result from injury to the myocardium, such as MI, chronic hypertension, or ischemia [6]. Though initially acting to mitigate damage, prolonged fibrosis weakens the heart muscle, ultimately driving the progression of HF. Thus, identifying circulating biomarkers of HF heavily involves the in-depth examination of myocardial remodelling driven by fibrosis, both of which will be discussed in detail in this review.

### 1.2. Heart Failure with Preserved Ejection Fraction and Heart Failure with Reduced Ejection Fraction

HF is historically subclassified into heart failure with preserved ejection fraction (HFpEF) and heart failure with reduced ejection fraction (HFrEF), which is defined by the left ventricle ejection fraction (LVEF) of a patient. LVEF is a measure of cardiac function, as it provides an indication of the pumping capability of the heart. The LVEF thresholds for HFpEF and HFrEF are ≥50% and ≤40%, respectively [1,2]. It is important to note that an additional subtype of HF, heart failure with mildly reduced ejection fraction (HFmrEF), was defined in 2016 by the AHA/ACC/HFSA and ESC, with LVEF sitting between 41% and 49% [7,8]. However, this review focuses upon HFpEF and HFrEF, as these definitions are used by the majority of published studies investigating circulating biomarkers. In addition to LVEF subtypes, HF can be further defined by the New York Heart Association (NYHA), in which the severity of HF is assessed by the level of physical activity tolerance [8]. Such an assessment is useful to understand general patient health; however, it offers no insights into the molecular pathology driving the change in cardiac function.

**Figure 1:**
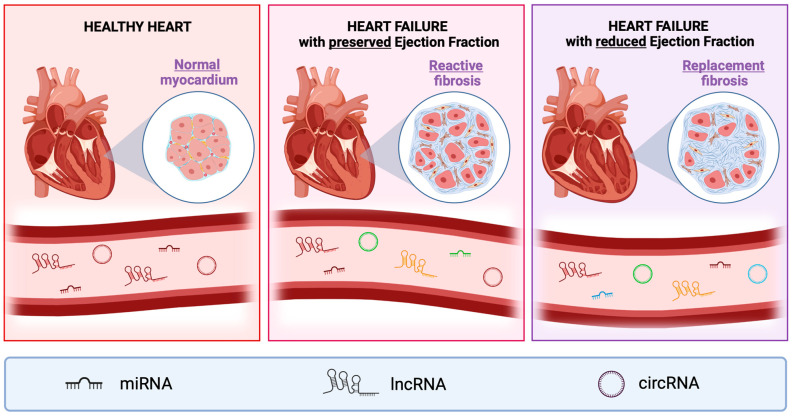
Structure of the heart under healthy and pathological conditions, with differing levels of circulating ncRNAs in the bloodstream. In the healthy heart, the myocardium is predominantly populated with cardiomyocytes. The bloodstream contains various ncRNAs, including microRNAs (miRNAs), long non-coding RNAs (lncRNAs), and circular RNAs (circRNAs). In HFpEF, the LV wall is thickened, and the myocardium undergoes reactive fibrosis. Circulating ncRNAs (miRNAs, lncRNAs, circRNAs) are altered in expression (indicated by different colours), indicating their role in the fibrotic and HF processes. In HFrEF, the LV wall is thinned and accompanied by replacement fibrosis, with loss of cardiomyocytes. The expression profiles of circulating ncRNAs are altered in HF, indicating their potential as biomarkers for disease subtypes [7,8,10]. Acronyms: HFpEF, heart failure with preserved ejection fraction; HFrEF, heart failure with reduced ejection fraction. Created in BioRender, https://BioRender.com/e51z267 (accessed on 27 December 2024).

The difference in the LVEF of HFpEF and HFrEF can be attributed to structural changes in the left ventricle (LV), as illustrated in Figure 1. Compared to a healthy heart, the LV is often thickened in the HFpEF. As the integrity of the LV is maintained, the pump function is likewise preserved. Conversely, the LV free wall is thin and weakened in HFrEF hearts, resulting in the significant impairment of the pumping ability [9].

### 1.3. Remodelling of the Heart During Heart Failure

Under conditions of increased workload, such as sustained hypertension or valvular dysfunction, the heart muscle may undergo hypertrophy, where cardiomyocytes enlarge to accommodate the heightened demands [11]. While this adaptation may initially assist the heart in meeting the increased demand, over time, hypertrophic muscle becomes less efficient and more susceptible to injury. Moreover, chronic injury—stemming from events like MI, ischemia, or prolonged hypertension—can lead to the development of fibrosis within the heart [12,13]. During fibrosis, healthy myocardial tissue is replaced by non-contractile scar tissue, which compromises the heart’s ability to contract, increases myocardial stiffness, and impairs diastolic function. In addition, persistent stress and damage may trigger cardiomyocyte apoptosis, further depleting healthy muscle tissue [14].

As the heart becomes progressively less efficient at pumping blood, compensatory mechanisms may lead to the expansion of the heart chambers, particularly the LV, to maintain cardiac output. In advanced HFrEF (Figure 1), the morphology of the heart may become more spherical, replacing the normal elliptical configuration [15]. This geometric distortion hampers the heart’s ability to generate adequate systolic pressure, further reducing cardiac output.

The clinical identification of cardiac remodelling is based on detecting morphological changes, including shifts in mass (such as hypertrophy and atrophy), chamber dimensions (including dilation), and geometry (heart wall thickness and shape), as well as the presence of fibrosis and inflammatory infiltrates [16].

### 1.4. Fibrosis in Heart Failure

Cardiac fibrosis is characterized by excessive ECM protein deposition [6,17]. Following myocardial injury, e.g., ischemia or MI, inflammatory responses are triggered to repair the myocardium [17]. This results in the differentiation of cardiac fibroblasts (CFs) into myofibroblasts, the activated form of CFs [17]. Excessive fibrosis results in the disruption of the normal structure and function of the heart, which can be subclassified into (1) reactive interstitial fibrosis, and (2) replacement fibrosis (Figure 1) [6]. Reactive interstitial fibrosis is mostly observed in HFpEF, which has been reported to have a greater degree of collagen cross-linking [18,19]. Replacement fibrosis is more commonly associated with HFrEF rather than HFpEF, as its pathology is largely driven by cardiomyocyte necrosis [18,19]. As reactive interstitial fibrosis is a result of progressive pressure overload, the identification of markers of this type of fibrosis could be used to inform the timing of clinical interventions to reduce the risk of CVDs resulting in myocardial injury [6].

Cardiac fibrosis-driven ECM deposition is commonly considered to be the accumulation of collagen. Collagen types I and III are fibrillar collagens that contribute to maintaining the structure of the myocardium [17,20]. When there is an excess of these types, cardiac function is impaired, due to the increased tissue stiffness [17]. Importantly, the ratio of collagen I:III seems to shift as HF progresses [21]. It has been suggested that this increase in collagen III lays the framework for collagen I deposition, further propelling cardiac remodelling [22]. Collagen type VI is non-fibrillar and has been reported to be elevated in HF [20,23], functioning to promote myofibroblast differentiation in vitro [23]. Another family of proteins key in regulating the ECM are matrix metalloproteinases (MMPs). MMP-2 and MMP-9 have been shown to be involved in LV remodelling [24]. MMP-2 is upregulated in non-ischemic DCM hearts, whereas MMP-9 is elevated in both ischemic and non-ischemic DCM hearts [24]. In other studies, increased circulating MMP-2 levels were observed to be associated with greater incidences of both HFpEF and HFrEF, while MMP-9 has been reported to drive extensive collagen deposition, significantly contributing to ECM remodelling [22,23]. Myocardial injury triggers an immune response, resulting in the presence of inflammatory molecules in the myocardium. In particular, Interleukin-11 (IL-11) plays a pivotal role in fibrosis, showing a positive correlation with the extent of myofibroblast activation [25]. While IL-11 has been shown to influence the activity of TGF-β, a known key driver of fibrosis, TGF-β has also been evidenced to upregulate IL-11 levels, thereby creating a pro-fibrotic feedback loop [25,26]. Together, the ECM proteins and inflammatory molecules stiffen the heart muscle and reduce its contractile ability, highlighting the major role of cardiac fibrosis in driving HF progression [6]. As these differences in the ECM landscape are likely to be significant, this raises the possibility of using their changes in expression levels as circulating biomarkers of HF diagnosis and severity.

### 1.5. Circulating Markers of Fibrosis: Unmet Needs and New Opportunities

The typical circulating biomarkers of cardiac disorders used in the clinical setting are B-type natriuretic peptide (BNP), its N-terminal cleaved form (NT-proBNP), and cardiac troponin (cTnT) [27,28]. As markers of ventricular stretch, BNP and NT-proBNP are elevated in most cardiac conditions and are thus nonspecific to HF (or types thereof) [8,28]. There is the further confounding issue that underlying comorbidities influence the level of these proteins, for instance, both BNP and NT-proBNP levels tend to be lower in obese individuals compared to non-obese people [8]. As such, BNP and NT-proBNP have a lowered sensitivity, making them suboptimal as biomarkers for HF alone [8]. High sensitivity cTnT assays are widely employed to detect MI, and a small increase has been found to be associated with HF [27,29]. Unfortunately, due to the lack of specificity of cTnT for diagnosing HF, it has limited use as a biomarker of HF.

Current diagnostic methods of HF rely on imaging methods (such as cardiac magnetic resonance (CMR) imaging), which are time consuming and require hospital visits. CMR not only provides visualization of the architecture of the heart, but can also be used to determine function [29]. Often, CMR is accompanied by echocardiography, which similarly informs on cardiac structure and function by utilizing ultrasound [29]. Though important for understanding HF disease status, these methods are not informative of pathological changes at the molecular level. To understand changes at this level, there is a need to examine biomarkers of HF.

Currently, there are no effective circulating biomarkers of cardiac fibrosis routinely used in the clinical setting [30]. As a common event in the progression of HF, identifying early signs of myocardial remodelling and fibrosis would potentially be beneficial in disease prognosis [6]. Fibrosis is largely driven by the enhanced recruitment and accumulation of the ECM and inflammatory proteins, whose expression can be influenced by ncRNAs [6,31]. As regulatory biological molecules, ncRNAs largely control gene and protein expression, and thus have the potential to alter the pathophysiology and prognosis of cardiac conditions like HF [31]. In the following section, we review the evidence in the literature supporting the influence of ncRNAs in HF and cardiac fibrosis, and evaluate their potential as circulating HF biomarkers.

## 2. ncRNAs

ncRNAs encompass a wide variety of RNA molecules that do not translate into proteins. Since their discovery, these molecules have been recognized as key regulators of numerous biological processes across various cell types and tissues [10,32,33]. ncRNA transcripts are classified based on their structural characteristics, mechanisms of action, biological functions, and sequence length similarities. This review focuses on the three most studied classes of ncRNAs, namely microRNAs (miRNAs), long non-coding RNAs (lncRNAs), and circular RNAs (circRNAs).

Abnormalities in ncRNA expression or function have been linked to the development of several diseases. Divergence in ncRNA expression between HF patients and non-HF individuals may be useful for the detection and diagnosis of HF and its subtypes [34]. Furthermore, deciphering ncRNA signatures in the heart may allow for a better understanding of the remodelling pathology and inform more tailored treatment options.

### 2.1. miRNAs

miRNAs are a class of small ncRNAs, typically comprising ~22 nucleotides, and they have intrinsic regulatory functions [35]. Under normal physiological conditions, miRNAs influence gene transcription and translation and maintain homeostasis [35]. Given the key role of miRNAs, it comes as no surprise that they can influence the pathophysiology of diseases, including cardiovascular disorders [36] and HF pathology [37]. Monitoring changes in miRNA expression levels in the bloodstream may thus inform disease diagnosis, as well as reflect disease severity. In this way, miRNAs have the potential of acting as circulating biomarkers, with the added advantage of having high stability and abundance in circulation [37]. Given that HF is a highly complex and heterogenous disease, identifying a combination of biomarkers to be tested may allow for more accurate and precise prognosis and diagnosis of the condition, thus informing clinical decisions targeted at halting disease progression. Due to the sheer number of miRNAs implicated in HF and fibrosis, this review pays particular attention to those with the most compelling clinical evidence. Information on miRNAs not mentioned in this review can be consulted in the following publications, among others: [38,39,40,41,42,43,44,45,46,47,48]. All miRNAs proposed as potential biomarkers in HF and their involvement in fibrosis are listed in Table 1. The tables in this review are organized to align the references with the corresponding data from the original articles within the same row, thereby facilitating a more transparent and coherent presentation of the information.

### 2.2. lncRNAs

lncRNAs are a heterogenous group of ncRNA molecules characterized by a length of more than 200 nucleotides [84]. Long dismissed as transcriptional noise, lncRNAs are now recognized as key gene regulators, which was revealed through advances in high-throughput and genome sequencing [85]. They exert effects through a variety of mechanisms, such as transcriptional and post-transcriptional modulation, chromatin remodelling, and epigenetic regulation [86]. Growing evidence highlights the role of lncRNAs in pathways associated with cardiovascular diseases, among which fibrosis, cardiac tissue remodelling, inflammation, and apoptosis are particularly significant [87,88].

lncRNAs can modulate gene expression by interacting with miRNAs, primarily through acting as molecular sponges that sequester miRNAs and inhibit their binding to target mRNAs. This results in the upregulation of the expression of specific genes that would otherwise be silenced by miRNAs. By modulating the availability of miRNAs, lncRNAs can influence a wide array of cellular processes, such as cell differentiation, apoptosis, and tissue remodelling [89,90].

Due to their involvement in critical HF-related processes and stability in the bloodstream [91], lncRNAs are emerging as potential biomarkers [92,93]. Changes in their expression can indicate the progression of HF, providing reliable diagnostic and prognostic information. Moreover, lncRNA expression profiles have the potential to guide the personalized treatment of HF, offering more tailored therapeutic options [94]. Table 2 provides an overview of lncRNAs proposed as potential biomarkers in HF and their involvement in fibrosis.

### 2.3. circRNAs

circRNAs are a class of ncRNAs generated through a non-canonical back-splicing mechanism, resulting in stable, covalently closed-loop structures that lack the typical 5′ caps and 3′ poly(A) tails found in linear RNAs [163]. Approximately half of circRNA-expressing host genes generate only a single circRNA isoform, while others produce multiple isoforms [164]. Increasing evidence suggests that circRNAs participate in various biological processes by sponging miRNAs and proteins (a process where circRNAs bind and sequester these molecules to regulate their activity), acting as scaffolds, regulating transcription and splicing, and serving as templates for protein synthesis [165]. Recent advances have deepened our understanding of circRNAs’ involvement in HF, with ongoing research revealing new insights, particularly regarding transcriptional and post-transcriptional regulation in cardiac disease progression [166,167,168]. The remarkable stability of circRNAs, coupled with their abundance in biological fluids, including blood plasma, makes them promising candidates as biomarkers [169,170]. However, the number of large-scale clinical studies investigating circRNAs as biomarkers of HF remains limited, highlighting the need for further research in this area. Table 3 summarizes the circRNAs suggested as potential biomarkers in HF and their roles in fibrosis.

## 3. ncRNAs as Circulating HF Biomarkers of and Their Role in Fibrotic Remodelling

### 3.1. miRNAs

The emerging role of ncRNAs as biomarkers for HF and other cardiac diseases is a rapidly advancing area of research, with various miRNAs, lncRNAs, and circRNAs demonstrating significant potential for disease diagnosis, prognosis, and monitoring treatment responses. Among various potential circulating HF biomarkers, miR-423-5p has shown promise and been examined in multiple studies [38,41,79]. Significant upregulation of this miRNA was observed in both the serum and plasma of HF patients compared to non-HF individuals [38,41]. In a separate study, miR-423-5p levels were drastically upregulated in HF patients experiencing dyspnoea compared to dyspnoeic non-HF patients, with an AUC of 0.85 [79]. The increase in the miR-423-5p level was also found to be correlated with the increasing severity of HF, as determined by NYHA classification [79], with one study reporting significantly elevated miR-423-5p levels in the plasma of NYHA class II, III, and IV HF patients compared to healthy controls, but not in NYHA class I patients [213]. Comparing chronic HF and non-HF groups, acute patients had significantly lowered levels of miR-423-5p, which were associated with worse clinical outcomes [214]. Adding to this, one study reported that a decrease in miR-423-5p in HF patients within 48 h post hospitalization was predictive of 180-day mortality (hazard ratio = 1.681; *p*-value = 0.002) [44]. Taken together, these results indicate the potential of this miRNA to determine the severity of HF in patients and measure LV remodelling and dilation.

Functionally, miR-423-5p inhibition in vitro reduced cardiomyocyte apoptosis and mitigated the mitochondrial dysfunction induced by hypoxia by activating the Wnt/β-catenin signalling pathway [81]. Similarly, the inhibition of miR-423-5p provided protective effects against the cardiomyocyte hypertrophy induced by angiotensin II via the targeting of the Ty 6 homologue (SUPT6H) in human cardiomyocytes [215]. Studies of miR-423-5p in cardiac fibrosis are limited, though in airway fibrosis, miR-423-5p overexpression promotes TGF-β1 expression, elucidating its possible signalling pathway [80].

**Figure 2:**
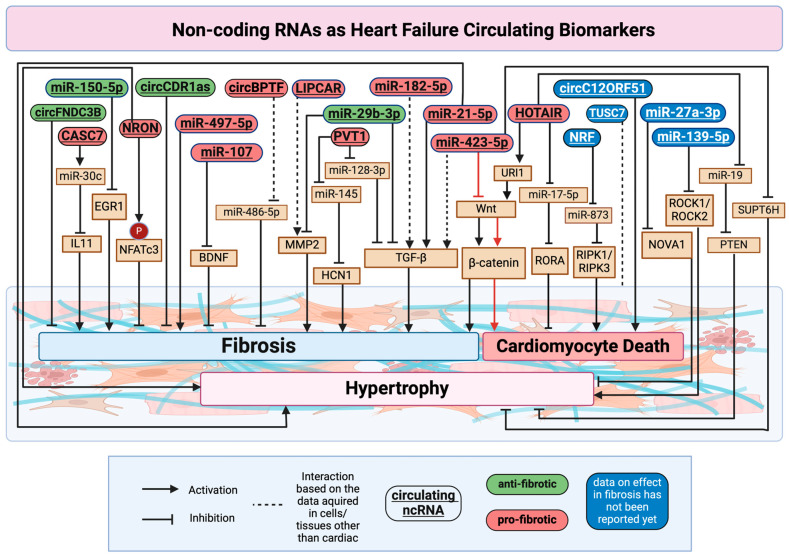
ncRNAs as HF circulating biomarkers. The diagram highlights how circulating ncRNAs act as biomarkers and regulators in HF by modulating hypertrophy (light pink bottom section), fibrosis (blue section), and cardiomyocyte death (dark pink section). Circulating ncRNAs are classified into anti-fibrotic (green ovals), pro-fibrotic (red ovals), and ones with unknown effects in fibrosis (blue ovals), while their specific targets are shown in beige boxes. Arrows and inhibition symbols represent their regulatory actions: activation (→): promotes the downstream target; inhibition (⊥): suppresses the downstream target; dashed lines (---): indicate interactions based on data from non-cardiac tissues or cells. Briefly, circFNDC3B, circCDR1as, miR-150-5p (via inhibiting EGR1), and miR-29b-5p (by inhibiting TGF-β and MMP2) suppress cardiac fibrosis [57,67,192,203,204], while CASC7 (by sponging miR-30c and upregulating IL-11), NRON (by promoting NFATc3 phosphorylation), miR-497-5p, miR-107 (by targeting BDNF), miR-21-5p, and PVT1 (by sponging miR-128-3p and miR-145 and upregulating HCN1 and TGF-β) promote cardiac fibrosis [39,52,83,104,141,144,145,219]. CircBPTF, LIPCAR, miR-182-5p, and miR-423-5p are shown as circulating in HF, with their role in fibrosis validated in other cells/tissues than just cardiac ones (vascular, lung) [73,80,124,187]. Similarly, miR-423-5p (via the Wnt/β-catenin pathway), NRF (by sponging miR-873 and upregulating RIPK1/3), and circC12ORF51 promote cardiomyocyte death [138,205,215], while HOTAIR (by sponging miR-17 and upregulating RORA) diminished it [116]. miR-21-5p and NRON, as well as iR-27a-3p and miR-423-5p, facilitate cardiac hypertrophy by downregulating their respective targets NOVA1 and SUPT6H [56,142,215,228]. Meanwhile, HOTAIR targets PTEN to regulate cardiac hypertrophy [222]. Acronyms: BDNF, brain-derived neurotrophic factor; BPTF, Bromodomain PHD Finger Transcription Factor; CASC7, Cancer Susceptibility Candidate 7; EGR1, Early Growth Response Protein 1; FNDC3B, Fibronectin Type III Domain Containing 3B; HCN1, Potassium/sodium hyperpolarization-activated cyclic nucleotide-gated channel 1; HOTAIR, HOX Transcript Antisense RNA; IL11, Interleukin 11; LIPCAR, Long Intergenic Non-Protein Coding RNA, Cardiac-Associated; MMP2, Matrix Metalloproteinase-2; NFATc3, Nuclear factor of activated T-cells, cytoplasmic 3; NOVA1, Neuro-oncological ventral antigen 1; NRON, non-coding repressor of NFAT; PTEN, Phosphatase and Tensin homologue; RIPK1/RIPK3, Receptor-Interacting Protein Kinase 1/3; ROCK1/ROCK2, Rho-associated protein kinase 1/2; RORA, Retinoic Acid Receptor-Related Orphan Receptor Alpha; SUPT6H, Suppressor of Ty 6 homologue; TGF-β1, Transforming growth factor beta 1; TUSC7, Tumor Suppressor Candidate 7; URI1, Unconventional Prefoldin RPB5 Interactor 1; and Wnt, Wingless and Int-1. Created in BioRender, https://BioRender.com/v93w259 (accessed on 27 December 2024).

Another miRNA involved in the TGF-β pathway and proposed as a circulating biomarker of HF is miR-21-5p (Figure 2). This highly expressed miRNA in cardiomyocytes is vastly reported to induce cardiac fibrosis [216,217]. mir-21-5p serum levels were shown to be correlated to the degree of left atrial fibrosis in atrial fibrillation patients (*n* = 175) [216]. In a study conducted by Marques, F. Z. et al., miR-21-5p was found to be released from the failing hearts of nine HF patients, and their plasma levels were compared to healthy controls (*n* = 8); a significant (1.96-fold) increase in the expression of hsa-miR-21-5p in HF patients was observed [45]. These results concur with those reported by Wong, L. L. et al. in 2019, albeit with a lower fold change of 1.17 [38]. The major mechanism by which hsa-miR-21-5p promotes cardiac fibrosis is through targeting the TGF-β/Smad signalling pathway [52].

In 2015, microarray analysis was performed on a cohort of 20 NYHA class II HF patients, 22 NYHA class III and IV HF patients, along with 15 healthy individuals as controls [48]. The expression of miR-182 was significantly elevated in HF serum and was observed to have superiority as a prognostic marker of HF over NT-proBNP (AUC 0.695 and 0.350, respectively) [48]. Further analysis revealed the potential of this miRNA as a predictor of mortality risk in HF patients [48]. A study in 2022 examined miR-182-5p expression in 82 HF patients and 78 matched healthy controls. The serum miR-182-5p concentration was found to be positively associated with circulating BNP levels and inversely related to LVEF [71]. Though miR-182-5p participates in a wide range of processes in the heart (regulating myocardial proliferation, migration, hypoxia, ischemia, apoptosis, and hypertrophy), the molecular mechanism through which it could regulate fibrosis is still not understood [73]. Interestingly, in lung fibrosis, the silencing of miR-182-5p reduced pathological remodelling via the TGF-β/Smad pathway, indicating that miR-182-5p is pro-fibrotic [72].

In contrast, the role of miR-497-5p in fibrosis has been described in multiple studies. Chen, X. et al. sought to identify the role of miR-497-5p in idiopathic pulmonary fibrosis [82]. Their research revealed a target gene of the miRNA that could functionally inhibit the expression of MMP2 and MMP9 [82]. Wong, L. L. et al. reported a 1.23-fold upregulation of miR-497-5p in HF patients compared to healthy controls [38]. The role of this miRNA in fibrosis is shown in other disease models and may be translatable to HF-associated cardiac fibrosis. Recently, the role of miR-497-5p as a circulating biomarker implicated in cardiac fibrosis was suggested by Tikhomirov, R. et al.; the miRNA was found upregulated in the blood plasma of aortic valve stenosis patients [83,218].

Some miRNAs that are considered potential biomarkers for HF are downregulated in the disease. miR-150-5p has been found to have significantly lower expression levels in HF patients compared to non-HF controls [38,39,67]. In a 2017 study by Scrutinio, D. et al., RT-qPCR was used to identify miRNA expression differences between 29 advanced HF patients (NYHA class III or IV), 25 mild to moderate HF patients (NYHA class I or II), and 15 healthy subjects [66]. They found that miR-150-5p was significantly downregulated in patients with advanced HF compared to healthy controls at a fold change of −2.0, as well as patients with milder and earlier stages of HF, with a fold change of −1.7 [66]. They further confirmed that no significant difference in expression was observed between mild HF patients and healthy controls [66]. Together, this suggests that miR-150-5p may be informative of advanced stages of HF and therefore a marker for HF severity. Furthermore, it was demonstrated that miR-150-5p levels showed a significant inverse correlation with the established cardiac biomarker NT-proBNP; however, unlike NT-proBNP, this miRNA was able to predict a poor prognosis of HF, as its levels were associated with a greater loss of pumping capability and mortality [66]. A reported downstream target of miR-150-5p is the early growth receptor gene 1 (EGR1), which has been implicated in promoting cardiac fibrosis [67]. The reduced expression of miR-150-5p in myocardial fibroblasts prevented the inhibition of EGR1 in vitro, thereby allowing the expression of fibrotic and ECM proteins, such as collagen 1, collagen 3, and MMP-13, ultimately contributing to the progression of myocardial fibroblasts [67]. Similarly, Deng, P. et al. observed a greater extent of cardiac dysfunction and fibrosis in miR-150-5p knockout mice compared to wild-type mice [68]. As this miRNA is fully conserved from mice to humans, this observation may also apply to human physiology.

Another example of a miRNA that is downregulated in the disease is miR-27a-3p. In 2014, Marfella, R. et al detected the expression of miR-27a-3p to be 22.8-fold lower in HF patients compared to healthy individuals [53]. Similarly, Ovchinnikova, E. S. et al. reported that the expression of miR-27a-3p was significantly reduced in patients with acute HF compared to healthy control individuals, but also compared to chronic HF patients [44]. However, this miRNA did not seem to be highly predictive of 180-day mortality. Functionally, miR-27a-3p promoted cardiac hypertrophy by decreasing NOVA1 (Neuro-oncological ventral antigen 1), and was a negative regulator of lung fibrosis [54,55,56].

In 2016, Marques et al. observed a notable 3.48-fold decrease in the expression of miR-29b-3p in nine HF patients compared to eight healthy individuals [45]. LV filling pressure was also found to be negatively correlated with the miR-29b-3p concentration [45]. In accordance with this, Wong, L. L. et al. later reported a 1.30-fold reduction in miR-29b-3p expression in HF patients compared to healthy individuals [38]. The miR-29 family has previously been shown to be involved in cardiac fibrosis and remodelling. In 2019, Liang, J. et al. experimentally verified TGF-β2 and Mmp2 as target genes of miR-29b-3p [57]. In the presence of miR-29b-3p mimic in cardiac fibroblasts, they observed the significant depletion of TGF-β2 and MMP2, both of which heavily influence and partake in the remodelling process of the myocardium. Members of the TGF-β family are key players in the fibrotic process, while MMP2 is a matrix protein and thus promotes ECM remodelling, and miR-29b-3p is proposed to target Smad3 signalling.

The downregulation of miR-107 has been reported in PBMCs and the plasma of chronic HF patients compared to non-HF controls [39,42]. A predicted in silico target of miR-107 in HF is brain-derived neurotrophic factor (BDNF) [39]. BDNF is crucial for the normal functioning of the heart due to its role in cardiac contractility [219]. Thus, its downregulation, for example, via miRNA deregulation, has been associated with pathological LV cardiac remodelling and corresponds to elevated levels of NT-proBNP [219]. However, like NT-proBNP, BDNF may be more suitable as a marker of CVDs in general, as its levels do not solely differ in HF patients. Low levels of BDNF influence the activities of cadherins and TNFα, both of which have been shown to play a role in atrial remodelling and thus potentially in LV remodelling [219].

miR-139-5p was observed to be downregulated in HF patients [39]. ROCK1 and ROCK2 have been predicted in silico as targets of miR-139-5p in HF, and their activities corresponded with the severity of HF, as indicated by the NYHA classification, LVEF, and severity of symptoms [39,61]. ROCK proteins have been implicated in driving inflammatory processes and contributing to cardiac hypertrophy [61].

### 3.2. lncRNAs

A large-scale study involving 788 patients, conducted by the Thum group, revealed that the long intergenic ncRNA predicting cardiac remodelling (LIPCAR) is upregulated during the late stages of post-MI remodelling and in chronic HF (CHF) patients. Furthermore, its expression was associated with a poor prognosis in HF patients [120]. A subsequent clinical trial confirmed the findings: LIPCAR plasma levels from 967 HF patients were significantly associated with functional impairment, as assessed via NYHA classification, and were significantly related to NT-proBNP and cTnT levels. Notably, the expression levels of LIPCAR were not different between the patients with HFpEF and HFrEF [121]. LIPCAR was also identified as a potential biomarker for early HF in post-AMI patients (*n* = 59) compared to non-HF post-AMI patients (*n* = 68) [122]. Moreover, LIPCAR levels, derived from plasma exosomes, were higher in patients characterized by LV remodelling one year post MI, compared to post-MI patients without LV remodelling [123]. The overexpression of LIPCAR in human vascular smooth muscle cells promoted cell proliferation and migration and enhanced the expression levels of MMP2 and MMP9 [124]. Together with findings from clinical studies, these results suggest that LIPCAR may act as a pro-fibrotic regulator [124].

Another pro-fibrotic circulating lncRNA is Cancer Susceptibility Candidate 7 (CASC7), found upregulated in both the plasma and PBMCs of HFpEF and HFrEF patients compared to healthy individuals (*n* = 62 per group) [104]. Functionally, lncRNA-CASC7 overexpression repressed miR-30c expression in H9c2 cells, thereby inhibiting the expression of pro-fibrotic cytokine IL-11 (Figure 2) [26]. A recent study also revealed CASC7’s ability to reduce myocardial apoptosis in myocardial ischemia–reperfusion rats by regulating miR-21 expression [105].

In a 2016 study by Greco, S. et al., HOX Transcript Antisense RNA (HOTAIR) was found to be downregulated in the peripheral blood mononuclear cells (PBMCs) of 25 patients with non-end-stage ischemic dilated cardiomyopathy HF compared to 18 age- and sex-matched controls. A similar downregulation of HOTAIR was observed in heart tissues from both end-stage and non-end-stage HF patients relative to healthy donors [96]. HOTAIR was also identified as a potential biomarker in patients with congenital heart disease and as an important mediator of AMI [93,220]. MI plays a crucial role in the development and progression of HF, as it can either be a primary cause or a major contributing factor. The role and molecular mechanisms of HOTAIR in cardiovascular diseases was fully discussed in a recent review [221]. For example, HOTAIR mitigated cardiomyocyte pyroptosis in HF mice through the miR-17-5p/RORA axis [116]. In cardiac hypertrophy models, HOTAIR functioned as a competing endogenous RNA (ceRNA) for miR-19, thereby regulating miRNA’s target PTEN [222]. It also sponged miR-34a in diabetic cardiomyopathy, restoring SIRT1 expression, which improved cardiac function and decreased oxidative stress and inflammation [115]. HOTAIR also contributes to fibrosis progression; a study by Pan, S.C. et al. suggested that HOTAIR enhances fibrosis by promoting URI1 expression and activating the Wnt pathway [223]. Similar results were observed by Tan, W. et al., who observed HOTAIR binding with PTBP1 and increasing the stability and expression of Wnt5a [114].

Tumor Suppressor Candidate 7 (TUSC7), gene derived lncRNA, also known as LOC285194 or LSAMP antisense RNA 3, was suggested as a potential biomarker for HF in a study by Greco, S. et al.; along with HOTAIR, TUSC7 was downregulated in non-end-stage ischemic dilated cardiomyopathy HF patients [96]. Targeting LOC285194 was reported to promote cell proliferation and inhibit apoptosis in human vascular smooth muscle cells (VSMCs) in vitro, suggesting it as a therapeutic target for atherosclerosis treatment [158]. However, the role of TUSC7 in the heart remains largely unknown.

Conversely, Greco, S. et al. observed a significant upregulation of Antisense Non-coding RNA in the INK4 Locus (ANRIL), also known as CDKN2B-AS1, in non-end-stage ischemic dilated cardiomyopathy HF patients [96]. Overall, ANRIL was widely implicated in clinical studies, especially involving CAD patients, which was already discussed in the review by Li, C. et al. [98]. Notably, Jiao, Y. et al. reported that ANRIL served as a more effective diagnostic marker than cardiac troponin I in patients with stable angina (*n* = 59). Furthermore, ANRIL levels were found to be higher in stable angina patients compared to those with MI (*n* = 59) [95]. The mechanism behind the ANRIL involvement in fibrosis was further elucidated: DNA (cytosine-5)-methyltransferase 1 methylates the lncRNA, leading to its reduced expression. This, in turn, activates the NLRP3/Caspase-1 pathway, facilitating myocardial fibrosis and promoting pyroptosis in cardiac fibroblasts [97].

The necrosis-related factor (NRF) lncRNA also plays a significant role in the development and progression of HF. Its expression was elevated in AMI patients with HF (*n* = 76) compared to those without HF (*n* = 58). Additionally, circulating lncRNA-NRF levels showed a positive correlation with serum NT-proBNP and TnI levels, while being negatively correlated with LVEF [137]. The suppression of NRF elevated miR-873 expression while lowering the levels of its target genes, RIPK1 and RIPK3, which significantly diminished myocardial necrosis (Figure 2) [138].

Another lncRNA associated with HF progression is plasmacytoma variant translocation 1 (PVT1), which exhibited upregulation in the serum of 92 CHF patients, compared to 60 healthy volunteers, while its direct target miR-190a-5p was decreased. Analysis of ROC curves identified PVT1 and miR-190a-5p as diagnostic markers for CHF, with their combination providing greater diagnostic precision than either marker individually [143]. PVT1 also contributes to the development of fibrosis through various pathways, including its role in promoting fibrosis by upregulating HCN1 (hyperpolarization-activated cyclic nucleotide-gated potassium/sodium channel 1) expression via miR-145 sponging [145]. In atrial fibrillation, PVT1 facilitates fibrosis through the miR-128-3p/SP1/TGF-β1 signalling axis [144]. It also plays role in cardiac remodelling via the miR-216/Ccnd3 signalling axis, exacerbating cardiomyocyte apoptosis [146].

Consequently, the non-coding repressor of NFAT (NRON) gene derived lncRNA acted as a predictor of HF severity. Xuan, L. et al. observed NRON to be upregulated in the blood plasma samples of 72 HF patients, compared to 60 non-HF control participants, using RT-PCR. The area under the ROC curve was 0.865, and NRON was negatively correlated with HDL (high-density lipoprotein) [130]. Conversely, the study by Gharbi, N. et al. reported atherosclerotic ischemic stroke patients to have decreased levels of NRON (*n* = 65 per group) [139]. The in vivo overexpression of NRON enhanced TAC-induced hypertrophy, while cardiomyocyte-specific NRON deletion reduced cardiac hypertrophy in mice [142]. NRON mitigated atrial fibrosis by suppressing the M1 macrophages activated by atrial myocytes and promoting NFATc3 phosphorylation [140,141].

### 3.3. circRNAs

In 2018, the Devaux group identified circPRDM5 (the main transcript variant in humans is hsa_circ_0005654) and other upregulated circular RNAs (cBPTF, cFNDC3B, cEXOC6B, cLAMA2-2, cPLCE1) as novel biomarkers for HF through an analysis of cardiac biopsies from ICM (*n* = 17) and DCM (*n* = 26) patients versus controls (*n* = 23) [186]. A later study by Liu, R. et al. reported circPRDM5 downregulation in serum samples from AMI patients (*n* = 18) compared to healthy controls (*n* = 60). CircPRDM5 showed an area under the ROC curve of 0.862 and no correlation with LVEF. Notably, in AMI patients (*n* = 77), circPRDM5 expression was significantly elevated on the first day after primary percutaneous coronary intervention (PCI) compared to pre-surgery levels [190]. Although direct evidence of circPRDM5’s role in cardiac tissues is lacking, it has been shown to promote the expression of fibrosis-related genes in human lens epithelial cells and to be upregulated following TGF-β2 treatment [191]. While circPRDM5 may play a role in fibrosis-related processes, its specific function in different tissues, including the heart, remains unclear and requires further investigation.

CircBPTF (hsa_circ_0000799) has been identified as a biomarker for HF in a patent by the Devaux group [186]. Similarly, studies have shown its significant upregulation in the cardiac tissues of patients with non-end-stage ischemic HF (IHF) (*n* = 12) compared to matched control subjects (*n* = 12). Additionally, circBPTF levels were also elevated in cardiac tissues from end-stage IHF patients (*n* = 36) when compared to their matched controls (*n* = 44). CircBPTF is reported to be well expressed in blood; however, there is a lack of follow-up studies on assessing circulating circBPTF in CVDs [186].

In vitro circBPTF (hsa_circ_0000799) was induced by hypoxia in HUVEC endothelial cells and was shown to target and decrease miR-196b-5p levels [165]. Interestingly, miR-196b-5p attenuated the TGF-β-induced increased expression of Col1a2 in mouse fibroblasts [224], indicting the potential pro-fibrotic role of circBPTF. A further circBPTF isoform, hsa_circ_0045462, serves as a miR-486-5p sponge and promotes hypoxic pulmonary arterial smooth muscle cell proliferation (Figure 2) [187]. Varied evidence suggests that miR-486-5p is anti-fibrotic, influencing fibroblast activation, inflammation, and the epithelial to mesenchymal transition [188,225,226]. CircBPTF therefore shows promise as a potential biomarker for HF and is likely a pro-fibrotic circular RNA. However, further research is required to fully understand its role and mechanisms in the heart.

CircFNDC3B, derived from the fibronectin type III domain-containing protein 3B (FNDC3B) gene, is among the most extensively studied circular RNAs. Emerging research has highlighted its diverse roles across various diseases, suggesting its potential as a biomarker [193]. CircFNDC3B (hsa_circ_0006156) has been recognized as a biomarker for HF by Devaux, Y.’s group in the patent [186]. Although circFNDC3B is known to be prominently expressed in blood, there is a notable gap in the research regarding the assessment of circulating circFNDC3B in the context of CVDs [186]. CircFNDC3B was significantly downregulated in the murine hearts after MI, while the AAV9-mediated overexpression of circFNDC3B enhanced neovascularization and reduced fibrosis after MI, suggesting its anti-fibrotic functions. Moreover, circFNDC3B in cardiac endothelial cells improved endothelial function and protected cardiomyocytes from death [192].

CircC12ORF51 (hsa_circ_0097435) is a circRNA produced from the C12orf51 gene. The study by Jiaqi Han revealed that hsa_circ_0097435 was upregulated in peripheral blood samples and plasma exosomes derived from HF patients (*n* = 40) compared to healthy individuals (*n* = 40), using RNA sequencing and RT-qPCR validation [205]. In loss-of-function and gain-of-function in vitro experiments, hsa_circ_0097435 silencing inhibited doxorubicin-induced myocardial apoptosis, while hsa_circ_0097435 overexpression promoted cardiomyocyte apoptosis. Additionally, hsa_circ_0097435 appears to contribute to HF, acting as a sponge for several miRNAs (miR-6799-5p, miR-5000-5p, miR-609, and miR-1294). While the role of hsa_circ_0097435 in fibrosis remains unexplored, it might exert an impact on fibroblasts through exosomes. Overall, these findings indicate that hsa_circ_0097435 holds promise as a blood-based biomarker and uncover a novel regulatory pathway associated with myocardial cell injury.

CircCDR1as is encoded by sequence antisense to the cerebellum degeneration-related antigen 1 (CDR1) gene [227]. CircCDR1as levels were demonstrated to be upregulated in patients with chronic HF (*n* = 30) compared to healthy controls (*n* = 30), using RT-qPCR in the study by Chen, C. et al. [202]. Moreover, circCDR1as functions as a molecular sponge for miR-135a and miR-135b, influencing the proliferation and apoptosis of human cardiomyocytes by modulating the miR-135/HMOX1 signalling pathways (Figure 2). These findings suggest that circCDR1as could serve as a promising biomarker for chronic HF. Another study by Gonzalez, C. et al. discussed the role of circCDR1as in macrophage cardiac inflammation and fibrosis. The researchers observed the downregulation of circCDR1as in cardiomyocytes and macrophages three days post MI in mice, while AAV9 circCDR1as administration in vivo significantly improved LVEF and decreased the fibrotic area at 3 and 4 weeks post MI [203]. A separate study by Mester-Tonczar, J. et al. reported similar effects of circular CDR1as in post-MI HF pig heart; treatment with the anti-fibrotic agent bufalin resulted in increased LVEF and elevated circRNAs’ CDR1as expression [204]. Moreover, positive correlations between CDR1as levels and LVEF, LV stroke volume, and a negative correlation with infarct size were observed. This evidence highlights the potential anti-fibrotic function of circCDR1as and its utility as a circulating biomarker for HF.

Similarly to circFNDC3B and circBPTF, circular MYO9A (circ_0036176) was upregulated in the myocardial tissues of HF patients (*n* = 24) compared to healthy organ donors (*n* = 18) [212]. However, any evidence of these circRNAs circulating in the blood is lacking. Notably, circ_0036176 features an internal ribosome entry site (IRES) and an open reading frame (ORF) of 627 nucleotides, which encodes a 208-amino acid protein referred to as Myo9a-208. This protein was found to mediate the suppressive effects of circ_0036176 on cardiac fibroblast proliferation, while miR-218-5p can bind to circ_0036176 and inhibit Myo9a-208 expression.

The involvement of ncRNAs in cardiac remodelling, proposed as circulating biomarkers for HF and discussed in Section 3, is illustrated in Figure 2.

## 4. ncRNAs Associated with HFpEF

**Figure 3:**
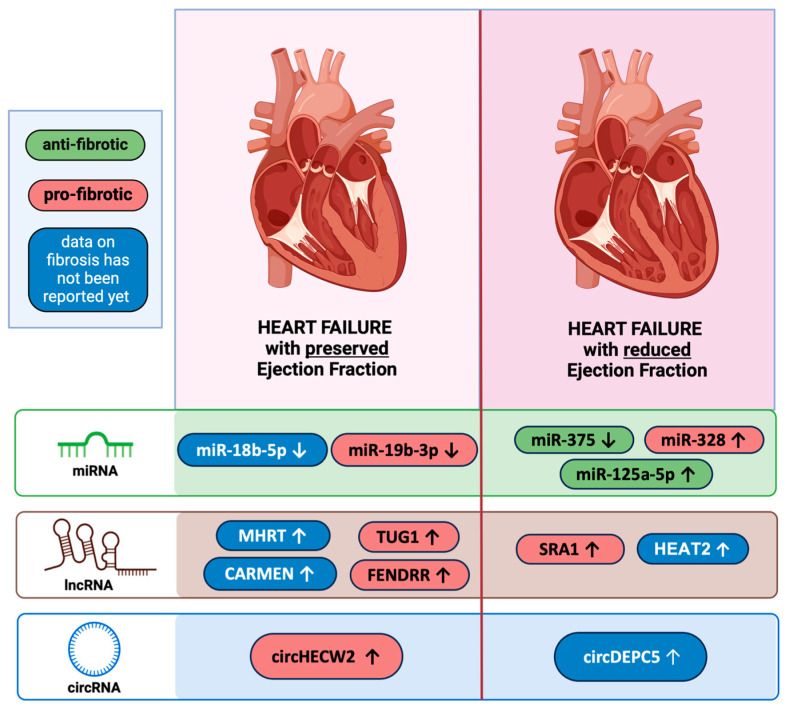
ncRNAs associated with HFpEF and HFrEF. The schematic highlights circulating molecular biomarkers associated with Heart Failure with preserved Ejection Fraction (HFpEF) and Heart Failure with reduced Ejection Fraction (HFrEF). Change in expression of ncRNAs are indicated with arrows (↑ for upregulation; ↓ for downregulation). For HFpEF, biomarkers such as miR-18b-5p and miR-19b-3p [38,50] are decreased, while lncRNAs (e.g., MHRT, CARMEN, TUG1, FENDRR) and circRNA circHECW2 are elevated [101,161,216]. Conversely, in HFrEF, miRNAs like miR-375, miR-328, and miR-125a-5p [43,46,74] are modulated, alongside increased lncRNAs (e.g., SRA1, HEAT2) and circRNA circDEPCS [92,149,171]. Acronyms: CARMEN, Cardiac Mesoderm Enhancer-associated Non-coding; DEPC5, DEP Domain Containing 5, GATOR1 Subcomplex Subunit; FENDRR, FOXF1 Adjacent Non-coding Developmental Regulatory RNA; HEAT2, heart-disease-associated transcript 2; HECW2, HECT, C2 And WW Domain Containing E3 Ubiquitin Protein Ligase 2; MHRT, myosin heavy-chain-associated RNA; SRA1, steroid receptor RNA activator 1; and TUG1, taurine upregulated 1. Created in BioRender, https://BioRender.com/f60l673 (accessed on 27 December 2024).

HFpEF is a complex condition, characterized by diastolic dysfunction and often accompanied by comorbidities like hypertension, diabetes, and obesity. Despite its prevalence, the molecular mechanisms underlying HFpEF remain poorly understood, and effective biomarkers for diagnosis and prognosis are lacking [229,230]. Recent research has highlighted the potential roles of ncRNAs in HFpEF pathophysiology, including reactive fibrosis progression [38,152,173]. This section examines ncRNAs involved in HFpEF and their potential as biomarkers (Figure 3).

In 2015, a cohort of 100 acute HF patients were identified with reduced miR-18b-5p 48 h after hospital admission, which was associated with a greater 180-day mortality rate (hazard ratio = 1.851; *p*-value = 0.013), hinting at its predictive value of mortality [44]. Wong, L. L. et al. observed a 1.28-fold downregulation of miR-18b-5p in HF patients compared to healthy individuals [38]. Furthermore, when comparing miRNA levels between HFpEF and HFrEF patients, HFpEF patients showed a 1.25-fold lowered expression level compared to HFrEF [38]. This raises the potential for this miRNA to differentiate patient diagnosis between the two HF subtypes.

The studies reporting miR-18b-5p’s functional role in the heart are scarce; however, an axis involving miR-18b-5p in diabetic nephropathy is important to note. lncRNA KCNQ1OT1 affected cell proliferation, apoptosis, and fibrosis through targeting miR-18b-5p [231]. KCNQ1OT1 (KCNQ1 overlapping transcript 1) serum levels were alleviated in patients with diabetic cardiomyopathy versus healthy individuals (*n* = 6 per group). Generally, in diabetic cardiomyopathy metabolic disturbances, including insulin resistance, hyperglycemia and altered lipid metabolism lead to structural and functional changes in the heart. The changes include diastolic dysfunction and the impaired relaxation of the LV, which are hallmark features of HFpEF [232]. Taken together with miR-18b-5p downregulation in HFpEF compared to HFrEF, it makes the investigation of KCNQ1OT1 in this context of HF particularly intriguing [233]. The clinical significance of KCNQ1OT1 was also evaluated in a cohort of 267 patients with coronary heart disease (CHD), compared to 50 individuals with unexplained chest pain (DC) and 50 healthy controls. KCNQ1OT1 was able to differentiate CHD patients from DCs, with an AUC of 0.757. Additionally, its expression was positively correlated with triglycerides, low-density lipoprotein cholesterol, cardiac troponin I, and C-reactive protein levels [118]. In HF mice, KCNQ1OT1 promoted cardiomyocyte apoptosis by targeting FUS [119]. Silencing KCNQ1OT1 in vitro and in vivo reduced pyroptosis and fibrosis via the miR-214-3p/caspase-1/TGF-β1 pathway [117].

miR-19b-3p has been identified as a ncRNA involved in the progression of HFpEF. In the 2019 study by Wong et al., miR-19b-3p was not only identified to be significantly downregulated in HF patients compared to non-HF controls, but was also significantly reduced by 1.17-fold in HFpEF individuals in comparison to those diagnosed with HFrEF [38]. Similarly, Paim, L. R. et al. observed differences in the expression of miR-19b-3p, with it being significantly downregulated in HFpEF individuals compared to HFrEF patients [50]. Furthermore, they showed that LVEF was inversely correlated with circulating levels of miR-19b-3p [50]. Taken together, these results highlight the potential of this miRNA in classifying HF patients into HFpEF/HFrEF. Regarding cardiac fibrosis-associated remodelling, the reports have been contradictory, as miR-19b-3p was also reported to be upregulated in the blood plasma of HCM patients with post-contrast T_1_ mapping < 470 ms (indicator of fibrotic remodelling in cardiac magnetic resonance imaging (MRI)), compared to HCM patients with T_1_ ≥ 470 ms (indicates non-fibrotic tissues in cardiac MRI) [51]. This suggests that miR-19b-3p might also change its expression levels throughout the development of different CVDs.

Taurine upregulated 1 (TUG1) lncRNA is among the ncRNAs with strong evidence supporting its utility as a potential biomarker for HFpEF. In the study of Zhang, S. et al., TUG1 and NT-proBNP were increased in the blood serum of 80 elderly hypertensive patients with HFpEF compared to age-matched hypertensive patients without HF [152]. Another study found that the differential expression of TUG1 and miR-145-5p reflects the severity of chronic HF and can predict 2-year survival prognosis. Specifically, lncRNA TUG1 was upregulated in 98 CHF patients with LVEF < 40% compared to 86 non-CHF participants, while miR-145-5p was downregulated. The 2-year follow-up revealed that patients with low TUG1 and high miR-145-5p expression had significantly better overall survival [153]. An intriguing target of TUG1 to be studied more in the future is miR-142-3p. In 2019, Su, Q. et al. determined that miR-142-3p is downregulated by the lncRNA TUG1, in turn resulting in the apoptosis and autophagy of cardiomyocytes [65]. However, miR-142-3p has been found to be increased in chronic HF patients with non-ischemic DCM, compared to controls and chronic HF patients with ischemic DCM [42]. Furthermore, increased expression of this miRNA was associated with higher rates of hospitalization due to the development of HF as an adverse cardiac event in STEMI patients [234]. Due to the conflicting reports with regard to the expression changes in this miRNA, combining two ncRNAs might provide a better diagnostic value for distinguishing HF subtypes, and elucidate the conflicting results.

TUG1 was also found to be upregulated in the blood plasma of AMI patients (*n* = 15—AMI, *n* = 18—healthy), and was shown to enhance cardiomyocyte apoptosis in myocardial ischemia/reperfusion injury in mice [154]. Functionally, TUG1 served as a competitive endogenous RNA for miR-9, and the silencing of lncRNA TUG1 reduced cardiomyocyte apoptosis by increasing miR-9 expression, which exerted anti-cardiomyocyte apoptotic affects by targeting Krüppel-like factor 5 (KLF5) [156]. Another mechanism of cardiomyocyte apoptosis involved TUG1 sequestering miR-132-3p, leading to the upregulation of HDAC3, which in turn, reduced H3K9 acetylation and epigenetically suppressed the expression of antioxidative genes such as Bcl-xL, Prdx2, and Hsp70 [157]. TUG1 has also been implicated in promoting myocardial fibrosis in mice through the recently identified CHI3L1/TUG1/miR-495-3p/ETS1 axis. Chitinase-3-like protein 1 (CHI3L1) upregulated TUG1 expression, which subsequently reduced the inhibitory effect of miR-495-3p on the protein C-ets-1 (ETS1) by acting as a molecular sponge, thereby facilitating the progression of myocardial fibrosis [155]. Another study revealed that TUG1 sponged pro-miR-29b-3p, and that the downregulation of anti-fibrotic miR-29b-3p, which inhibits TGF-β1, counteracted TUG1’s effects on cardiac fibroblast (CF) proliferation [58]. In conclusion, lncRNA TUG1 acts as a versatile regulator involved in cardiac hypertrophy and fibrosis, highlighting its potential role as a key contributor to the development of HF.

Several clinical studies have also suggested the power of the myosin heavy-chain-associated RNA transcript (MHRT) gene derived from lncRNA to act as a predictor of HF severity. Xuan, L. et al. observed MHRT (and another lncRNA NRON) to be upregulated in the blood plasma samples of 72 HF patients, compared to 60 non-HF control participants [130]. Similarly, MHRT (along with lncRNA FENDRR and CARMEN) was upregulated in the PBMCs of hypertensive patients with HFpEF (*n* = 55) compared to healthy volunteers (*n* = 25), but no change was observed compared to hypertensive patients without signs of HF (*n* = 23) [102]. In another study by Zhang, L. et al., the plasma levels of chronic HF patients (*n* = 88) were downregulated compared to healthy controls (*n* = 65), with an AUC of 0.9295. A follow-up study revealed that chronic HF patients with lower levels of lncRNA MHRT expression had poorer survival outcomes compared to those with higher expression levels [129]. Notably, single nucleotide polymorphisms (SNPs) (rs7140721, rs3729829, and rs3729825) in the MHRT gene were associated with the risk and prognosis of chronic HF [235]. MHRT was also linked with AMI in the study of Zhang, J. et al.; the lncRNA was upregulated in AMI patients (*n* = 47) versus healthy volunteers (*n* = 28), while in vitro MHRT was reported to inhibit the apoptosis of cardiomyocytes [131]. There are conflicting reports regarding the role of MHRT in fibrosis. In a pressure overload TAC mouse model, MHRT was found to reduce cardiac hypertrophy and fibrosis by inhibiting Brg1, a key component of the pathological stress-activated Brg1-Hdac-Parp chromatin repressor complex [132]. Conversely, MHRT was increased in MI mice, and in vitro studies revealed that MHRT overexpression promoted collagen production and cardiac fibroblast proliferation through miR-3185, while silencing MHRT had the opposite effect [133]. These findings, combined with clinical studies, indicate the presence of redundant pathways involving MHRT in MI and HF, highlighting potential new directions for research.

CARMEN, or Cardiac Mesoderm Enhancer-associated Non-coding RNA, is a super enhancer-associated lncRNA, playing a crucial role in the differentiation of cardiac precursor cells into cardiomyocytes [103]. Together with FENDRR and MHRT, CARMEN was found to be upregulated in the PBMCs of hypertensive patients with HFpEF (*n* = 55) compared to healthy volunteers (*n* = 25) [102]. Although CARMEN expression was increased during pathological remodelling in both mouse and human hearts, its role in fibroblasts still requires further investigation [103].

In addition to potential biomarker function in essential hypertension and HFpEF patients [102], FENDRR or FOXF1 Adjacent Non-coding Developmental Regulatory RNA is an important player in cardiac fibrosis. FENDRR was upregulated in the heart tissues of TAC mice, while its silencing significantly reduced fibrotic remodelling though inhibiting the miR-106b/Smad3 pathway [108]. Conversely, FENDRR demonstrated a protective effect in the heart, as its overexpression mitigated H_2_O_2_-induced damage in cardiomyocytes, shown by enhanced cell viability and reduced cell apoptosis [109].

Research on the role of circRNAs in differentiating between HFpEF and HFrEF is still in its early stages and remains scarce (Figure 3). The RNA sequencing of epicardial adipose tissue samples obtained from patients with HFpEF (*n* = 5) and patients without HF (*n* = 5) revealed a total of 131 differentially expressed circRNAs. Hsa_circ_0118464, corresponding to the HECW2 gene, showed the highest-fold change of 36 during qPCR validation [173]. Another study revealed that circHECW2 isoform hsa_circ_0057576 is upregulated in CAD patients (*n* = 3) compared to healthy controls (*n* = 3), and inhibits hsa-miR-130a-3p expression. Notably, hsa-miR-130a-3p reduces the inflammatory and fibrotic response in pulmonary fibrosis by regulating the proinflammatory factor TNF-α and the pro-fibrotic receptor TGF-βRII [174]. In the TAC mouse model, mmu-Hecw2_0009, a circRNA also derived from the Hecw2 gene, was found to play a role in the progression of both fibrosis and hypertrophy [175].

The overview of ncRNAs reported as circulating biomarkers in HFpEF and HFrEF is illustrated in Figure 3.

## 5. ncRNAs Associated with HFrEF

HFrEF is a severe and debilitating condition characterized by the heart’s inability to effectively pump blood, leading to decreased cardiac output. This impairment in heart function results in heightened morbidity and mortality, making it a major public health concern [236]. Despite recent advancements in treatments, including novel therapies such as SGLT2 inhibitors, vericiguat, and transcatheter mitral valve repair, the prognosis for patients with HFrEF remains poor, with a 5-year survival rate of only 25% following hospitalization [237]. Over the years, considerable effort has been devoted to identifying biomarkers that can aid in the diagnosis, prognosis, and differentiation of various HF subtypes [238]. This section focuses on key ncRNAs and their associations with HFrEF. Gaining insight into the molecular profiles of these RNA molecules offers the potential to enhance early diagnosis, refine prognostic assessments, and enable the development of targeted therapies for HF (Figure 3).

In 2015, Watson, C. J. et al. observed a significant reduction in the levels of circulating miR-375 in all HF patients compared to the non-HF cohort, with this reduction reaching a greater extent in HFrEF patients compared to those with HFpEF [43]. They further demonstrated the potential of using miR-375 alone and in combination with BNP and other miRNAs for diagnosing HF and distinguishing between HFpEF and HFrEF [43]. With regard to HFpEF/HFrEF distinction, BNP alone had an AUC of 0.66 [43]. miR-375 displayed an AUC of 0.75 on its own, which was similar to that in combination with BNP (AUC 0.78), and it improved to 0.86 in the presence of three other miRNAs [43]. As opposed to the findings of Watson et al., Wong, L. L. et al. saw an upregulation of miR-375 in HF patients by 1.44-fold compared to healthy individuals. Wong, L. L. et al. performed miRNA profiling in the plasma of HF patients (*n* = 58) and healthy controls (*n* = 28) [46]. They also identified hsa-miR-125a-5p to be significantly upregulated in HFrEF patients compared to control and HFpEF patients, an observation that was verified in a validation cohort (HF, *n* = 60; healthy, *n* = 30) [46]. This was further shown in their later study, which reported a 1.17-fold increase in expression in HFrEF patients compared to HFpEF [38]. However, there have been contradictory reports regarding the expression levels of this miRNA in HF. In the same study, Wong, L. L. et al. observed an overall 1.14-fold reduction in hsa-miR-125a-5p expression in HF patients compared to healthy individuals [38]. In agreement with this, Galluzzo et al. reported 0.69-fold decrease in expression in HF patients (*n* = 30) relative to age- and gender-matched healthy controls (*n* = 36) [47]. In 2022, Vilella-Figuerola et al. observed the expression of miR-125a-5p to be significantly downregulated in the plasma of chronic HF patients compared with healthy controls [39]. It has been suggested that miR-125a-5p has anti-fibrotic properties [60]. In a myocardial ischemia/reperfusion swine and murine model, the delivery of the miRNA resulted in improved cardiac function and limited fibroblast proliferation, and thus the remodelling of the myocardium [60]. In its turn, miR-375 protected cardiomyocytes following hypoxic-reoxygenation injury by reducing caspase-3 activity [78]. Though the role of miR-375 requires further validation, it acted as anti-fibrotic in lung fibrosis by inhibiting the Wnt/β-catenin pathway [77].

Another miRNA associated with HFrEF is miR-328. The upregulation of miR-328 in acute MI patients was associated with an increased risk of HFrEF development after 6 months, with this ability of miR-328 to predict patients with HF onset to those without showing an AUC of 0.762 [74]. Whether this also occurs in patients who have not experienced AMI in the past remains to be elucidated. Notably, cardiomyocyte-derived miR-328 fostered cardiac fibrosis via the paracrine regulation of neighbouring fibroblasts [75]. Moreover, exosomal miR-328-3p derived from MI cardiomyocytes promoted apoptosis through caspase signalling [76].

Among lncRNAs, steroid receptor RNA activator 1 (SRA1) lncRNA was markedly elevated in CHF patients (*n* = 93) versus healthy individuals *(n* = 63), and showed a positive correlation with BNP levels, left atrial diameter, and LV end-diastolic diameter, while being negatively associated with LVEF. Importantly, SRA1 demonstrated significant potential for distinguishing between subtypes of CHF patients, effectively identifying HFrEF patients (AUC = 0.891) as well as HFpEF and HFmrEF patients (AUC = 0.652) compared to healthy controls. Notably, SRA1 also showed an ability to differentiate between HFrEF patients and those with HFpEF or HFmrEF (AUC = 0.778) [149]. SRA1 is pro-fibrotic—it facilitates the activation of cardiac myofibroblasts by downregulating miR-148b [150]. It also reduced hypoxia-induced damage in cardiomyocytes by modulating the PPARγ/NF-κB signalling pathway [151].

HEAT2 is another lncRNA found to be associated with HFrEF. A study by Boeckel, J. et al. reported the regulation of the lncRNA heart-disease-associated transcript 2 (HEAT2) in HFrEF patients with DCM (*n* = 6) and ICM (*n* = 10) compared to elderly individuals (*n* = 8). In a larger cohort study (*n* = 69—HFrEF, *n* = 38—controls), HEAT2 expression levels have a discriminatory power to predict the presence of HFrEF with an AUC of 0.705, and predicted mortality with an AUC of 0.712 [92]. The role of HEAT2 in cardiac function is yet to be fully elucidated.

Similarly to HFpEF, clinical studies linking circRNAs specifically to HFrEF are scarce (Figure 3). The study of Zhang, C. compared circRNA expression profiles in blood plasma obtained from patients with HF (*n* = 3) compared to healthy donors (*n* = 3) using circRNA microarrays [171]. Among the 696 differentially expressed circRNAs, the authors emphasized circDEPC5 (hsa_circ_0062960), which was further validated through RT-qPCR in plasma samples from a cohort of healthy individuals (*n* = 30) and HF patients with EF < 40% (*n* = 30). This circRNA demonstrated an AUC of 0.838 for the ROC curve and showed a strong correlation with the serum levels of BNP. The roles of circDEPC5 are still not fully understood. Notably, Depcd5 gene knockout in mice has been linked to blood and lymphatic vascular abnormalities [172].

## 6. ncRNAs Associated with Cardiac Remodelling

**Figure 4:**
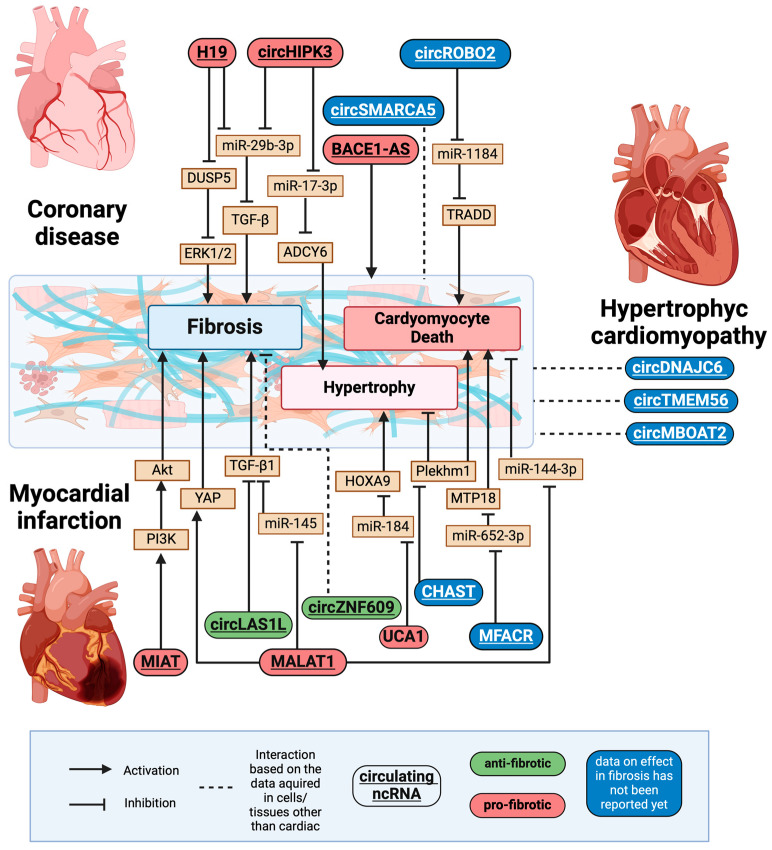
Circulating ncRNAs associated with cardiac remodelling. The figure depicts the involvement of circulating ncRNAs across three HF-related conditions (coronary disease, myocardial infarction, and hypertrophic cardiomyopathy) in regulating fibrosis (blue section), cardiomyocyte death (dark pink section), and hypertrophy (light pink). Circulating ncRNAs are classified into anti-fibrotic (green ovals), pro-fibrotic (red ovals), and ones with unknown effects in fibrosis (blue ovals), while their specific targets are shown in beige boxes. Arrows and inhibition symbols represent their regulatory actions: activation (→): promotes the downstream target; inhibition (⊥): suppresses the downstream target; and dashed lines (---): indicate interactions based on data from non-cardiac tissues or cells. Briefly, among ncRNAs circulating in myocardial infarction, MIAT (via PI3K/Akt), MALAT (by activating YAP and TGF-β), and UCA1 (in liver) are pro-fibrotic [110,127,135,159,160,250], while circLAS1L and circZNF609 are anti-fibrotic (the latter is demonstrated in lung fibrosis) [166,185,206]. CHAST and MFACR have not yet been reported as playing a role in fibrosis, though CHAST promotes hypertrophy and represses cardiomyocyte death by downregulating Plekhm1. UCA1 facilities hypertrophy via miR-184/HOXA9 and MFACR acts as a positive hypertrophy regulator via miR-652-3p/MTP18 [106,107,159,161,208,209]. MALAT1 also increases apoptosis via miR-144-3p [128]. Among the ncRNAs circulating in coronary disease, H19 and circHIPK3 are pro-fibrotic by upregulating TGF-β [112,198,244,254], whereas circROBO2 promotes cardiomyocyte death [210]. The roles of circDNAJ6, circTMEM56, and circMBOAT2 in the heart, shown circulating in hypertrophic cardiomyopathy, remain to be further elucidated [207]. Acronyms: ADCY6, Adenylyl cyclase type 6; Akt, Protein kinase B; BACE1-AS, Beta-Secretase-1 Antisense RNA; CHAST, Cardiac Hypertrophy-Associated Transcript; DNAJC6, DnaJ heat shock protein family (Hsp40) member C6; DUSP5, Dual specificity protein phosphatase 5; ERK1/2, Extracellular signal-regulated kinases; HIPK3, Homeodomain-Interacting Protein Kinase 3; LAS1L, LAS1-Like Ribosome Biogenesis Factor; MBOAT2, Membrane-Bound O-Acyltransferase Domain Containing 2; MTP18, Mitochondrial protein 18 kDa; HOXA9, Homeobox protein Hox-A9; MALAT1, Metastasis-associated lung adenocarcinoma transcript 1; MFACR, Mitochondrial fission and apoptosis-related circRNA; MIAT, MI-associated transcript; PI3K, Phosphoinositide 3-kinases; Plekhm1, Pleckstrin homology domain-containing family M member 1; TGF-β, Transforming growth factor β; ROBO2, Roundabout Guidance Receptor 2; SMARCA5, SWI/SNF-Related, Matrix-Associated, Actin-Dependent Regulator of Chromatin Subfamily A Member 5; TMEM56, Transmembrane Protein 56; TRADD, Tumor necrosis factor receptor type 1-associated DEATH domain protein; UCA1, urothelial carcinoma-associated 1; YAP, Yes-associated protein; and ZNF609, Zinc Finger Protein 609. Created in BioRender, https://BioRender.com/r50y649 (accessed on 28 December 2024).

Cardiac remodelling is a crucial process in the pathogenesis of various heart diseases, referring to the structural and functional changes that occur in the heart due to injury or stress, such as hypertension, coronary disease, MI, and HF. These changes often involve alterations in myocardial structure, fibrosis, hypertrophy, and apoptosis, which ultimately impair cardiac function and contribute to disease progression [16]. In this section, we examine lncRNAs and circRNAs found in patients who do not yet have HF, but are affected by cardiovascular conditions that could potentially progress to HF (Figure 4).

### 6.1. Circulating ncRNAs Associated with Coronary Diseases

Coronary artery disease (CAD) is a leading risk factor for the onset and progression of HF. The development of HF in patients with CAD is primarily driven by the gradual reduction in blood flow to the heart muscle, resulting in ischemia, myocardial injury, and subsequent cardiac remodelling [239]. Over time, this can impair the heart’s ability to pump blood effectively, contributing to both the onset of HF and its worsening.

Circular RNA ROBO2 (hsa_circ_0124644) was initially identified as upregulated in coronary artery disease (CAD) patients compared to healthy controls (*n* = 12 per group) via RNA microarray analysis. Subsequent validation in larger cohorts (115 healthy vs. 137 CAD) using RT-qPCR confirmed its significance. Including circROBO2 with traditional CAD risk factors enhanced diagnostic accuracy, achieving an AUC of 0.804 [210]. Mechanistically, circROBO2 knockdown in mice reduced cardiomyocyte apoptosis by upregulating miR-1184, which subsequently suppressed TRADD expression in the myocardium following MI [211]. Another study suggested that circROBO2 facilitated the proliferation and migration of human aortic smooth muscle cells by activating NF-κB signalling [240].

Two groups have recently shown the relevance of circular RNA SMARCA5 (hsa_circ_0001445) for coronary artery (heart) disease (CAD or CHD) in clinical studies. The earlier study by Vilades, D. et al. showed that the plasma levels of hsa_circ_0001445 were decreased in patients with a greater extent and severity of coronary atherosclerosis, based on a cohort of 200 patients with suspected stable CAD [177]. Remarkably, hsa_circ_0001445 exhibited remarkable stability; at room temperature, minimal effects were observed after storing the plasma samples for 72 h. Moreover, hsa_circ_0001445 implementation was able to correctly reclassify the patients misclassified by a multiparameter model of stable CAD, based on clinical history and cardiovascular risk factors. A consecutive study revealed hsa_circ_0001445 to be downregulated in the peripheral blood leukocytes of CHD patients (*n* = 94) compared to healthy controls (*n* = 126) [178]. ROC curve analyses showed the AUC for hsa_circ_0001445 to be 0.816. These findings prove that hsa_circ_0001445 is a prominent candidate as a biomarker of coronary heart disease.

Another ncRNA playing a role in the progression of CAD is lncRNA H19. The current evidence suggests that H19 is involved in various mechanisms linked to the development and genetic regulation of cardiovascular pathology [241]. Its levels were increased in the plasma of CAD patients compared to the plasma of individuals with normal coronary arteries (*n* = 300—CAD, *n* = 180 control subjects) [242]. PBMC-derived H19 also promoted AMI, another important contributor to HF, and taken together with lncRNAs MIAT and MALAT1, which also exerted elevated expression levels, was suggested as novel biomarker of this CVD [110]. Moreover, in clinical studies, circulating H19 levels could distinguish pulmonary arterial hypertension patients (*n* = 52—existing cohort; *n* = 75—validation cohort) from controls (*n* = 57—existing cohort, *n* = 54—validation cohort), correlated with right ventricular function, and serve as a predictor of long-term survival [111]. lncRNA H19 is highly conserved and abundant in cardiac tissues [243]. In the mouse model H19-miR-675 axis, targeting CaMKIIδ acted as a negative regulator of cardiac hypertrophy [113]. lncRNA H19 also plays a role in the progression of cardiac fibrosis; its upregulation enhances the proliferation and synthesis of ECM-related proteins by inhibiting the miR-29a-3p/miR-29b-3p-VEGFA/TGF-β axis, as well as H19 promoting fibrosis via the repression of DUSP5/ERK1/2 [59,112]. Overall, lncRNA H19 holds potential as a CVD biomarker due to its role in key pathological processes, particularly when considered alongside other ncRNAs like MIAT and MALAT1. However, further clinical studies are required to fully establish its utility in HF.

One more intriguing example of a promising HF biomarker is Beta-Secretase-1 Antisense RNA (BACE1-AS), the lncRNA antisense to the Beta-Secretase-1 (BACE1) gene, which encodes a key enzyme in β-amyloid production associated with Alzheimer’s disease [100]. BACE1-AS, derived from PBMCs, was suggested as an independent predictor of major adverse cardiovascular events in high-cardiovascular-risk patients (the study population consisted of 259 non-CVD patients, 90 stable CAD patients, and 85 acute coronary syndrome patients (ACS)) [99]. Moreover, BACE1-AS was upregulated in LV biopsies from non-end-stage ischemic HF patients (*n* = 18) compared to non-CVD donors (*n* = 17) [101]. The transcriptomic analysis of cells overexpressing BACE1-AS highlighted alterations in the TGFβ, TNFα, p38, and EGFR signalling pathways, suggesting the potential role of the BACE1-AS in pathological remodelling and fibrosis.

A further circRNA involved in cardiac fibrosis and associated with coronary diseases is circular RNA homeodomain interacting protein kinase 3 (circHIPK3) [244]. Silencing circHIPK3 has been shown to reduce the proliferation and migration of cardiac fibroblasts, as well as alleviate cardiac fibrosis in both in vitro and in vivo models by releasing anti-fibrotic miR-29b-3p (Figure 4) [198]. In a post-MI HF mouse model, circHIPK3 was increased and enhanced the effects of adrenaline in HF via the miR-17-3p-ADCY6 axis [245]. Overall, circHIPK3 was shown to inhibit the development of atherosclerosis, myocardial injury, and MI, but it also contributed to cardiomyopathy, myocardial fibrosis, and HF, which was fully discussed in the review by Zhang, L. [244]. A research study by Liu, X. described hsa_circ_0000284 (circHIPK3) and hsa_circ_0075269 (circRUFY1), both carried by exosomes, as potential biomarkers that could differentiate between chronic coronary syndrome patients (*n* = 135) and non-cardiac chest pain patients (*n* = 83) [195]. Another study by Bazan, H. A. identified that the circHIPK3 (hsa_circ_0000284, labelled as circR-284 in the article) to miR-221 ratio has potential as a diagnostic biomarker of carotid plaque rupture and stroke. Stable carotid plaques feature a necrotic core, covered by fibrous caps composed of VSMCs within the collagen-rich matrix, formed as a result of intimal thickening in response to arterial inflammation [194]. miR-221 promotes intimal thickening by downregulating p27^Kip1^, a cyclin-dependent kinase inhibitor that restricts VSMC cell cycle progression [246]. In the study, the ratio of circR-284–mir-221 expression levels was able to discriminate between both asymptomatic (*n* = 47) and symptomatic patients (*n* = 24) and permitted the identification of patients with a recent cerebrovascular event (*n* = 41) with an AUC of 0.98. A research paper by Chen, M. showed hsa_circ_0000284 as a risk factor and potential biomarker for prehypertension and hypertension [196]. Hypertension, chronic coronary syndrome, and carotid plaque rupture are significant contributors to cardiovascular events, including MI and HF. Considering circHIPK3’s diverse roles, it is a strong candidate for further investigation as a biomarker in HF studies.

### 6.2. Circulating ncRNAs Associated with Myocardial Infarction

MI can directly and indirectly lead to the progression of HF through a combination of myocardial damage, ventricular remodelling, and neurohormonal changes [247]. The early detection of pathological cardiac remodelling is crucial for managing HF in patients following a MI.

Metastasis-associated lung adenocarcinoma transcript 1 (MALAT1), also known as non-coding, nuclear-enriched abundant transcript 2 (NEAT2), is among the well-known lncRNAs involved in numerous biological processes [248]. At least two studies identified MALAT1 as a potential biomarker of AMI [110,125]. A study by Li, R. et al. reported the upregulation of MALAT1 in a cohort of 160 AMI patients compared to 50 angina pectoris patients. MALAT1 demonstrated strong potential for distinguishing AMI patients from controls, with an AUC of 0.823, and was positively correlated with C-reactive protein, low-density lipoprotein cholesterol, cardiac troponin I, and infarct size. Notably, MALAT1 polymorphism rs3200401 predicted major adverse cardiac and cerebrovascular events in AMI patients [249]. A recent review by Li, Y. et al. describes MALAT1 as a regulator of fibrosis in various pathologies, including CVD [250]. In brief, lncRNA MALAT1 acts as a pro-fibrotic regulator, for example, MALAT1 was upregulated in high-glucose treated CFs and facilitated the nuclear translocation of YAP, promoting cardiac fibrosis [127]. In cardiomyocytes, MALAT1 promoted apoptosis after MI by sponging miR-144-3p (Figure 4) [128]. In summary, these findings suggest MALAT1 as a promising biomarker for AMI and a potential indicator for HF, although further large-scale studies are required.

MI-associated transcript (MIAT) lncRNA expression, along with H19 and MALAT1 expression, was higher in AMI patients compared to healthy controls [110]. Patients with acute ST-segment–elevation MI or STEMI (*n* = 274) had lower levels of MIAT in PBMCs when compared with patients with non-ST-segment–elevation MI or NSTEMI (*n* = 140) [134]. MIAT is a pro-fibrotic lncRNA; its silencing reduces cardiac fibrosis and alleviates HF via PI3K/Akt [90,135]. In diabetic cardiomyopathy, the upregulation of glucose-induced lncRNA-MIAT contributes to the reduction in miR-214-3p’s inhibitory effect on proinflammatory IL-17 expression [136,251].

Circulating levels of Cardiac Hypertrophy-Associated Transcript (CHAST) were increased in 53 early-stage (24 h after the stroke) AMI patients versus 90 controls, and served as a predictor of LV contractile function and cardiac remodelling [106]. The early detection of pathological cardiac remodelling is crucial for managing HF in patients following MI. CHAST was primarily identified as a hypertrophy-associated transcript in the research paper by Viereck, J. et al.; CHAST was upregulated in hypertrophic heart tissues from aortic stenosis patients and in the cardiomyocytes of TAC mice. The overexpression of CHAST was found to be enough to trigger cardiomyocyte hypertrophy both in vitro and in vivo, whereas silencing CHAST mitigated the pathological cardiac remodelling caused by TAC [107]. Functionally, CHAST repressed autophagy regulator Plekhm1 (Pleckstrin homology domain-containing family M member), preventing cardiomyocyte autophagy and facilitating hypertrophy [252].

Finally, lncRNA UCA1 (lncRNA urothelial carcinoma-associated 1) was also associated with MI; its levels were decreased at early stages and increased at 3 days in AMI patients [159]. Moreover, UCA1 and SARRAH (SCOT1-antisense RNA regulated during ageing in the heart) were upregulated in the atrial fibrillation patients compared with patients without a history of atrial fibrillation [147]. Atrial fibrillation (AF), the most common arrhythmia, is characterized by irregular and rapid electrical impulses in the atria, leading to ineffective atrial contractions. AF and HF are closely linked, with each one worsening the other [253]. Functionally, the in vivo overexpression of SARRAH facilitated recovery following AMI [148]. On molecular level, UCA1 facilitated cardiomyocyte hypertrophy via the miR-184/HOXA9 axis and enhanced cardiomyocyte proliferation by suppressing the miR-128/SUZ12/P27 signalling pathway [161,162]. Though the significance of UCA1 in cardiac fibrosis remains to be fully characterized, UCA1 was found to promote the progression of liver fibrosis via the miR18a/Smad3/TGF-β1 pathway [160].

Among the circular RNAs implicated in AMI, stands out circRNA ZNF609, in particular its isoform hsa_circ_0000615, also known as MICRA—Myocardial Infarction-associated Circular RNA. A comprehensive study by the Devaux group found that the MICRA levels were significantly lower in blood samples from acute MI patients (AMI) (*n* = 642) compared to those from healthy volunteers (*n* = 86). Patients with reduced levels of MICRA were at higher risk of LV dysfunction [184]. A subsequent study by the same group examined AMI patients categorized by ejection fraction: reduced (≤40%, *n* = 87), mid-range (41–49%, *n* = 106), and preserved (≥50%, *n* = 297). MICRA demonstrated its ability to enhance risk stratification following MI, reinforcing its potential as a valuable biomarker for future prognostic strategies [166]. CircZNF609 also holds promise as a potential biomarker for CAD; its levels were significantly lower in patients with CAD (*n* = 330) compared to healthy controls (*n* = 209) [183]. In addition, circZNF609 expression levels demonstrated a significant association with C-reactive protein levels and lymphocyte counts. Moreover, the overexpression of circZNF609 in vitro led to a decrease in the expression of IL-6 and TNF-α, while IL-10 levels increased, demonstrating the anti-inflammatory role of circZNF609 [183]. Another study demonstrated that circZNF609 was downregulated in activated fibroblasts, while its overexpression attenuated lung fibrosis in vivo via the miR-145-5p/KLF4 axis and circZNF609-encoded peptides [185]. To summarize, circRNA ZNF609 (MICRA) exhibits both anti-fibrotic and anti-inflammatory effects and demonstrates diagnostic and therapeutic potential, providing promising opportunities for future research and clinical applications.

Mitochondrial fission and apoptosis-related circRNA (MFACR) was significantly upregulated in AMI patients, while miR-125b was downregulated, compared to healthy controls (*n* = 61 per group) [208]. In mouse cardiomyocytes, this circRNA regulates mitochondrial fission and apoptosis in the heart by directly targeting and downregulating miR-652-3p; this in turn blocks mitochondrial fission and cardiomyocyte cell death by suppressing MTP18 translation (Figure 4) [209].

One more circRNA found circulating in AMI and contributing to fibrosis progression is circLAS1L (hsa_circ_0090876). Its expression was found to be significantly downregulated in AMI patients (*n* = 30) compared to healthy controls (*n* = 30) via RT-qPCR [206]. Importantly, circLAS1L overexpression inhibited human fibroblast proliferation and migration, and promoted apoptosis. On molecular level, circLAS1L inhibited the expression of pro-fibrotic markers α-SMA, collagen I, and collagen III. These findings indicate that circLAS1L exhibits a cardioprotective anti-fibrotic effect, though its specific role in fibrosis associated with HF remains to be further explored.

### 6.3. Circulating ncRNAs Associated with Hypertrophic Cardiomyopathy

Another cardiac disease in which fibrosis plays a significant role in contributing to progression to HF is hypertrophic cardiomyopathy (HCM). Several circRNAs were identified in the study of Sonneschein, K. as potential biomarkers for HCM. The serum expression levels of circDNAJC6, circTMEM56, and circMBOAT2 were downregulated in 64 patients with HCM (among which 31 were diagnosed with obstructive HCM or HOCM) compared to 53 healthy control individuals (Figure 4) [207]. Moreover, circTMEM56 and circDNAJC6 could serve as indicators of disease severity, as they show a negative correlation with echocardiographic parameters in HOCM. However, the functions of these circRNAs in the heart require further clarification.

In conclusion, circulating ncRNAs are promising candidates for the early detection, prognosis, and potential treatment of HF, particularly in patients with underlying cardiovascular conditions such as CAD, MI, and HCM. However, additional large-scale clinical studies are necessary to confirm their clinical applicability and to explore their therapeutic potential in the management of HF.

The overview of the ncRNAs discussed in this section is illustrated in Figure 4.

## 7. ncRNAs Involved in Fibrosis Mechanisms in the Heart

Numerous ncRNAs have been linked to fibrotic remodelling in cardiac disease, but not all are established as circulating biomarkers. For example, circRNA_010567 was shown to reduce levels of miR-141, which targets TGF-β1. Moreover, circRNA_010567 silencing was associated with the decreased expression of Col I, Col III, and α-SMA [255,256,257]. CircRNA_000203 also demonstrated pro-fibrotic features by sponging miR-26b-5p, which interacts with 3′UTRs of Col1a2 and CTGF [258]. CircCAMTA1 was upregulated in the atrial muscle tissues of atrial fibrosis patients and functionally facilitated fibrosis by downregulating the inhibitory effect of miR-214-3p on Transforming growth factor β receptor 1 (TGFBR1) expression [259]. In the rat model of MI, circPAN3 enhanced cardiac fibrosis by sponging miR-221 and upregulating FoxO3 [260]. CircHelz promoted fibrosis by directly binding to Yes-associated protein (YAP) and facilitating its translocation to the nucleus to promote fibroblast growth and proliferation [261]. Another recently discovered potential target for the treatment of cardiac fibrosis is circNSD1, involved in remodelling progression via the miR-429-3p/SULF1/Wnt/β-catenin signalling pathway [262]. Among the anti-fibrotic circRNAs, it is worth mentioning mmu_circ_0005019 (circPcca), which exerted its function by sponging miR-499-5p [263]. CircCELF1 alleviated myocardial fibrosis by reducing the m^6^A methylation level of DKK2 (Dickkopf WNT signalling pathway inhibitor 2) through the upregulation of FTO expression. This inhibited the binding of miR-636 to DKK2, leading to increased DKK2 expression and reduced fibrosis [264]. Another example of anti-fibrotic circRNAs is circBMP2K, which enhances miR-455-3p expression, suppresses SUMO1 expression, and ultimately inhibits the activation, proliferation, and migration of cardiac fibroblasts [265].

CircNFIB is an important player in cardiac fibrosis development; it decreased in a post-MI mouse model, as well in primary adult cardiac fibroblasts treated with TGF-β, suggesting its anti-fibrotic properties. Moreover, circNFIB upregulation was demonstrated to reduce cardiac fibrosis in vitro by sponging miR-433, while the inhibition of circNFIB exhibited the opposite results, showing that circNFIB is critical for protection against cardiac fibrosis [200]. The study by Jiu Liu revealed endogenous SO_2_ to enhance circNFIB expression in neonatal rat cardiac fibroblasts, which in turn suppressed the Wnt/β-catenin and p38 MAPK pathways, thereby mitigating cardiac fibrosis [201]. Although circNFIB plays a critical role in fibrosis, its implication as a circulating biomarker in HF remains unexplored. The Zheng, M. RNA sequencing study found circNFIB (hsa_circ_0086376) to be downregulated in the adipose tissues of CAD patients with HF (*n* = 5) compared to CAD non-HF patients (*n* = 5) [199]. Further research is needed to clarify the role of circNFIB in HF and fibrosis in patients.

Among the lncRNAs, colorectal neoplasia differentially expressed (CRNDE) lncRNA expression, was negatively correlated with COL1A1 expression in 376 human heart tissues, and mechanistically inhibited the binding of Smad3 to the α-SMA gene promoter, attenuating cardiac fibrosis [266]. It also played a role in cardiac remodelling by protecting cardiomyocytes from apoptosis in HF by regulating high mobility group box-1 (HMGB1) cytoplasm translocation through poly-ADP-ribose polymerase 1 (PARP-1) [267]. Another CRNDE signalling pathway involved the miR-489-3p/Nrf2 axis, which suppressed MI injury progression [268]. Notably, CRNDE was proposed as a circulating biomarker for colorectal cancer; however, studies of this lncRNA as a blood-based biomarker in CVD are scarce.

Wisp2 super-enhancer-associated RNA (WISPER) expression has been linked to severe fibrosis in cardiac biopsies of patients with aortic stenosis, underscoring its potential as a clinical biomarker and a promising target for anti-fibrotic therapy. On a molecular level, WISPER acts as a super enhancer, controlling critical fibrosis-related genes, including COL1A1, COL3A1, FN1, and aSMA, in cardiac fibroblasts [269]. Notably, a recent study proposed the synthetic antisense oligonucleotide HTX-001, which inhibits WISPER, to reduce cardiac fibrosis and improve heart function [270].

Pro-fibrotic lncRNA (PFL) presents a promising target for future clinical research, as its expression was upregulated in an in vivo MI model. Additionally, silencing PFL reduced TGF-β1-induced myofibroblast formation and fibrogenesis by acting as a sponge for let-7d [162].

An appealing combination of ncRNAs was proposed in the research paper by Zeng, Y., who identified circAMOTL1 (hsa_circ_0004214) as being upregulated in neonatal hearts compared to mature hearts [179]. On the other hand, circFOXO3 (hsa_circ_0006404) is expressed at higher levels in the heart tissues of older individuals compared to younger ones [181]. The combined potential of these circRNAs to evaluate the severity of HF might be studied in the future. Subsequent studies demonstrated that circAMOTL1 contributes to cardiac fibrosis; one of the proposed mechanisms involves its interaction with EIF4A3 and stabilizing MARCKS expression, as shown in a streptozotocin-induced diabetes mouse model of diabetic cardiomyopathy [180]. Another in vitro identified pathway involves circAMOTL1 suppressing miR-330-3p and enhancing *Smad7* expression [271]. In its turn, the overexpression of circFOXO3 in mouse embryonic fibroblasts promoted senescence, while its knockdown suppressed it. CircFOXO3 interferes with the activity of anti-stress proteins HIF1α, FAK, E2F1, and ID-1, driving cellular senescence and cardiac fibrosis [181,272]. The knockdown of circFOXO3 has also been shown to reduce cardiac ischemia–reperfusion (I/R) injury during heart transplantation and improve heart graft function in vivo [273], while in the context of MI-related cardiac dysfunction in vivo, circFoxo3 alleviated I/R injury by targeting the KAT7/HMGB1 axis [182]. Together, these findings highlight the potential role of circFOXO3 as a biomarker for HF.

## 8. Conclusions and Future Perspectives

Many of the ncRNAs described in this review have been shown to be involved in other conditions, both cardiac-related and non-cardiac-related diseases. For example, the lncRNA PVT1 has been shown to promote gallbladder cancer progression by targeting miR-18b-5p [274]. miR-107 has been shown to partake in Alzheimer’s disease through regulating amyloid-β levels [275,276]. miR-21-5p is highly abundant in the body and its circulating levels have been implicated in several types of cancers, such as pancreatic and oral [277,278]. Similarly, circSMARCA5 has been implicated in multiple cancers such as hepatocellular carcinoma and colorectal cancer [279,280]. It is also pertinent to mention that ncRNAs extend beyond the microRNAs, lncRNAs, and circRNAs, discussed in this review, and also feature other subtypes, among which are P-element Induced Wimpy testis-interacting RNAs (piRNAs), recently suggested as fibrosis regulators and promising biomarkers of CVD [281,282,283].

Due to their inherent lack of organ specificity, a single circulating ncRNA cannot be diagnostic of HF with high accuracy. To circumvent this challenge, a multiplexed biomarker detection approach may be the key. Multiple studies have evidenced the benefits of combining several ncRNAs for increased diagnostic accuracy. In the 2015 study by Watson et al., the specificity and diagnostic value of BNP for HF was significantly enhanced by the inclusion of one or more of the miRNAs (miR-375, miR-146a, miR-30c, miR-328, miR-221) investigated in the study [43]. This further demonstrated the significance of combining BNP with two or more of these miRNAs to improve the ability to distinguish between HFpEF and HFrEF, compared to using BNP alone [43]. Similarly, the power of combining multiple miRNAs to distinguish between HFpEF and HFrEF was shown by Vilella-Figuerola et al. [39]. Their analysis indicated the discriminative values of the following two combinations of miRNAs: (1) miR-107, miR-139-5p, and miR-150-5p; and (2) let-7a-5p, miR-107, miR-125a-5p, miR-139-5p, miR-150-5p, miR-30b-5p, and miR-342-3p [39]. The latter combination further distinguished chronic HF patients by their underlying aetiologies, namely ischemic or non-ischemic [39].

Several emerging technologies could potentially provide the solution to address this issue. These include the nanopore DNA-barcoded biomarker detection platform developed by Oxford Nanopore Technologies (ONT) [284,285], the Simoa Bead Technology developed by Quanterix [286], and the Luminex Multiplexed Assay developed by R&D Systems [287]. The ONT nanopore platform can be adapted to combine biomarker detection with next generation sequencing to decipher and quantify the composition of biomarkers in a patient sample [284,285]. Simoa Bead Technology utilizes magnetic beads and antibodies to screen for biomarkers in a sample, even at extremely low concentrations [286]. Similarly, the Luminex Multiplexed Assay detects biomarkers with the use of magnetic beads and specific antibodies [287]. The multiplexed feature of these platforms allows for the simultaneous detection of multiple biomarkers, gearing towards a more high-throughput approach and fostering promising potential in enhancing confidence in the diagnosis of heterogenous and complex diseases like HF.

Standardized pre-analytical and analytical parameters are critical for ensuring reliable and reproducible results when measuring HF biomarkers. These parameters help to mitigate the variability caused by sample handling, processing, and assay performance. Proper standardization minimizes the errors caused by sample collection, storage, and assay variability, thereby enhancing the clinical utility and reliability of biomarker measurements. A recent paper from the EU-CardioRNA COST action CA17129 provided contributions on this issue [288].

Another issue regarding the use of ncRNAs as disease biomarkers that is evident from the contrasting studies presented in this review is that the degree and direction of change in the levels of circulating ncRNAs can vary substantially among HF patients, even though they may present with the same cardiac condition. This high variability is attributable to several factors, including individual patient characteristics such as age, gender, ethnicity, etc., the stage and subtype of the disease, the ncRNA detection method used, and more [42]. As such, setting a threshold pathological value for individual ncRNAs may not be entirely appropriate for the diagnosis of HF. Rather, a personalized approach may be crucial to address this issue. Though seemingly tedious, the availability of the bespoke platforms mentioned above makes the task more plausible. With increasing technological advancements, assays quality is improving and the cost of sequencing techniques is reducing. Along with increased computing power, these technological advancements allow for more comprehensive data processing and analysis at the point of use [284]. Together, these qualities offer potential for exploiting such technologies to produce individual patient biomarker fingerprints for more accurate diagnosis, if adopted in the clinical setting.

## Figures and Tables

**Table 1:** miRNAs as potential biomarkers of HF and their role in fibrosis progression.

miRNA	Change in Expression	Sample Type (Human)	Patient Experimental Group	Patient Control Group	Fibrosis	Other Functions	Study
miR-18b-5p	↓ (all HF and HFpEF relative to HFrEF)	Plasma	HFrEF, *n* = 180 HFpEF, *n* = 158	Healthy, *n* = 208			[38]
	↓	Plasma	AHF, *n* = 100	Healthy, *n* = 24			[44]
						Upregulated in atrial fibrillation in humans	[49]
miR-19b-3p	↓ (all HF and HFpEF relative to HFrEF)	Plasma	HFrEF, *n* = 180 HFpEF, *n* = 158	Healthy, *n* = 208			[38]
	↓ (HFpEF relative to HFrEF)	Serum	HFrEF, *n* = 31 HFpEF, *n* = 36			Inverse correlation with LVEF	[50]
	↑	Plasma	HCM, *n* = 27 T_1_ < 470 ms (patients likely to have diffuse cardiac fibrosis)	HCM, *n* = 28, T_1_ ≥ 470 ms (patients unlikely to have cardiac fibrosis)	Potentially PF: suggested as biomarker for diffuse fibrosis in hypertrophic cardiomyopathy in humans		[51]
miR-21-5p	↑ (all HF)	Plasma	HFrEF, *n* = 180 HFpEF, *n* = 158	Healthy, *n* = 208			[38]
	↑	Plasma	HF, *n* = 9	Healthy, *n* = 8			[45]
					PF: promotes cardiac fibrosis in rat H9c2 cells via TGF-β/Smad signalling pathway		[52]
miR-27a-3p	↓ (all AHF; discovery and validation cohorts)	Plasma	AHF, *n* = 100	Healthy, *n* = 24			[44]
	↓ (all HF relative to healthy and non-HF)	Plasma	HF, *n* = 81	Healthy, *n* = 15 Non-HF, *n* = 60			[53]
					Potentially AF: negatively regulated lung fibrosis in human fibroblasts and bleomycin-treated mice		[54,55]
						Promoted cardiac hypertrophy by decreasing NOVA1 in mice	[56]
miR-29b-3p	↓ (all HF)	Plasma	HFrEF, *n* = 180 HFpEF, *n* = 158	Healthy, *n* = 208			[38]
	↓	Plasma	HF, *n* = 9	Healthy, *n* = 8			[45]
					AF: overexpression in mouse CFs reduced fibrosis by targeting TGF-β2 and MMP2		[57]
						lncRNAs TUG1 and H19 act as competing endogenous RNAs formiR-29b-3p to inhibit its anti-fibrotic role	[58,59]
miR-107	↓ (all CHF)	Plasma	CHF, *n* = 46	Non-CHF, *n* = 26			[39]
	↓ (all CHF)	PBMC	CHF with NIDCM, *n* = 19 CHF with ICM, *n* = 15	Non-CHF, *n* = 19			[42]
					Potentially PF: miR-107 in silico was predicted to downregulate BDNF, which may result in pathological LV cardiac remodelling		[39]
miR-125a-5p	↓ (all HF)	Plasma	HFrEF, *n* = 180 HFpEF, *n* = 158	Healthy, *n* = 208			[38]
	↓	Plasma	CHF, *n* = 46	Non-CHF, *n* = 26			[39]
	↑ (HFrEF relative to HFpEF and healthy)	Plasma	HFrEF, *n* = 30 HFpEF, *n* = 30	Healthy, *n* = 30			[46]
	↓	Plasma	HF, *n* = 30	Healthy, *n* = 36			[47]
					AF: overexpression in myocardial ischemia/reperfusion mice improved cardiac function and limited fibroblast proliferation	Targeted Klf13, Tgfbr1, and Daam1 to regulate macrophage functions, fibroblasts, and endothelial cells in mice	[60]
miR-139-5p	↓	Plasma	CHF, *n* = 46	Non-CHF, *n* = 26		In silico targeted ROCK1 and ROCK2, to potentially promote inflammation and cardiac hypertrophy	[39,61]
					Potentially AF: overexpression in vivo reduced liver fibrosis in mice and in human uterine leiomyoma cells		[62,63]
miR-142-3p	↑	PBMC	CHF with NIDCM, *n* = 19	Non-CHF, *n* = 19			[42]
					AF: reduced high-salt-induced cardiac fibrosis in rats		[64]
						miR-142-3p targeted by TUG1, resulting in apoptosis and autophagy of mouse cardiomyocytes	[65]
miR-150-5p	↓ (all HF)	Plasma	HFrEF, *n* = 180 HFpEF, *n* = 158	Healthy, *n* = 208			[38]
	↓	Plasma	CHF, *n* = 46	Non-CHF, *n* = 26			[39]
	↓ (advanced HF relative to mild/moderate HF and healthy)	Serum	Advanced HF, *n* = 29 Mild/moderate HF, *n* = 25	Healthy, *n* = 15			[66]
					AF: targeted EGR1 to promote fibrosis in human CFs and in MI mice		[67]
						Knockout of miR-150 in mice resulted in cardiac dysfunction and fibrosis	[68]
miR-181b-5p	↓ (all HF)	Plasma	HFrEF, *n* = 180 HFpEF, *n* = 158	Healthy, *n* = 208			[38]
	↑	Plasma	HF, *n* = 9	Healthy, *n* = 8			[45]
					PF: miR-181b antagomir reduced atrial fibrosis in TGF-β-transgenic mice		[69]
						Overexpression in HF rats reduced inflammation by downregulating TNF-α, IL-1β, and IL-6	[70]
miR-182-5p	↑	Serum	HF, *n* = 42	Healthy, *n* = 15			[48]
	↑	Serum	HF, *n* = 82	Healthy, *n* = 78			[71]
					PF (lungs): silencing miR-182-5p reduced pathological remodelling via TGF-β/Smad pathway in human embryonic fibroblasts and bleomycin-treated mice		[72]
						Regulated myocardial proliferation, migration, hypoxia, apoptosis, and hypertrophy	[73]
miR-328	↓ (all HF)	Serum	HFrEF, *n* = 75 HFpEF, *n* = 75	Non-HF, *n* = 75			[43]
	↑	Plasma	AMI (high risk of HF), *n* = 359	Healthy, *n* = 30			[74]
					PF: induced cardiac fibrosis via paracrine regulation of neighbouring fibroblasts in mice		[75]
						Exosomal miR-328-3p from MI mice cardiomyocytes promoted apoptosis through caspase signalling	[76]
miR-375	↑ (all HF)	Plasma	HFrEF, *n* = 180 HFpEF, *n* = 158	Healthy, *n* = 208			[38]
	↓ (HFrEF relative to HFpEF and non-HF)	Serum	HFrEF, *n* = 75 HFpEF, *n* = 75	Non-HF, *n* = 75			[43]
					AF (lungs): inhibited Wnt/β-catenin pathway in rat alveolar epithelial cells		[77]
						Protected cardiomyocytes following hypoxic injury by reducing caspase-3 activity in mice	[78]
miR-423-5p	↑ (all HF)	Plasma	HFrEF, *n* = 180 HFpEF, *n* = 158	Healthy, *n* = 208			[38]
	↑	Serum	CHF, *n* = 30	Non-CHF, *n* = 30			[41]
	↑ (all HF relative to healthy and non-HF)	Plasma	HF, *n* = 30	Healthy, *n* = 39 Non-HF (dyspoenic), *n* = 20			[79]
					Potentially PF: promoted airway fibrosis via upregulation of TGF-β in human epithelial cells		[80]
						Silencing of miR-423-5p in rat H9c2 cells reduced cardiomyocyte apoptosis by activating Wnt/β-catenin signalling pathway	[81]
miR-497-5p	↑ (all HF)	Plasma	HFrEF, *n* = 180 HFpEF, *n* = 158	Healthy, *n* = 208			[38]
					PF: targeted Mmp2 and Mmp9 in mice to promote pulmonary fibrosis		[82]
						Circulating biomarker of cardiac fibrosis in AVS patients	[83]

miRNAs are sorted in ascending order. HF, heart failure; HFrEF, heart failure with reduced ejection fraction; HFpEF, heart failure with preserved ejection fraction; HCM, hypertrophic cardiomyopathy; DCM, dilated cardiomyopathy; AMI, acute myocardial infarction; MI, myocardial infarction; AVS, aortic valve stenosis; PBMC, peripheral blood mononuclear cell; LV, left ventricle; LVEF, left ventricle ejection fraction; BDNF, brain-derived neurotrophic factor; Col1a1, collagen type I alpha 1; Daam1, dishevelled associated activator of morphogenesis 1; EGR1, Early Growth Response 1; IL-1β, Interleukin-1 beta; IL-6, Interleukin 6; Klf13, Krüppel-like factor 13; Mmp2, Matrix metallopeptidase 2; Mmp9, Matrix metallopeptidase 9; NOVA1, Neuro-oncological ventral antigen 1; ROCK1, Rho-associated coiled-coil containing protein kinase 1; ROCK2, Rho-associated coiled-coil containing protein kinase 2; Smad, suppressor of mothers against decapentaplegic homologue; TAC, Transverse Aortic Constriction; TGF-β, Transforming growth factor beta; Tgfbr1, TGF beta receptor 1; TNF-α, Tumor necrosis factor; TUG1, taurine upregulated 1; Wnt, Wingless and Int-1; PF, pro-fibrotic; AF, anti-fibrotic; ↑, upregulation; and ↓, downregulation.

**Table 2:** lncRNAs as potential biomarkers of HF and their role in fibrosis progression.

lncRNA	Change in Expression	Sample Type (Human)	Patient Experimental Group	Patient Control Group	Fibrosis	Other Functions	Study
ANRIL (CDKN2B-AS1)	↑ (stable angina and MI)	Plasma	Stable angina, *n* = 59 MI, *n* = 62	Healthy, *n* = 48			[95]
	↑	PBMC	Non-end-stage IDC HF, *n* = 25	Healthy, *n* = 18			[96]
					AF: ANRIL epigenetic silencing promoted cardiac fibrosis in mice		[97]
						A potential biomarker in CAD, contributes to atherosclerosis development	[98]
BACE1-AS	↑ (CAD and ACS)	PBMC	CAD, *n* = 90 ACS, *n* = 85	Non-CVD, *n* = 259			[99]
					PF: elevated in LV biopsies of HF patients compared to non-CVD donors	Transcriptomic analysis of cells overexpressing BACE1-AS highlighted alterations in TGFβ-, TNFα-, p38-, and EGFR signalling pathways	[100,101]
CARMEN	↑ (compared to healthy, but not EH, no HFpEF)	PBMC	EH + HFpEF, *n* = 55	EH, no HFpEF, *n* = 23 Healthy, *n* = 25			[102]
					CARMEN expression was increased during pathological remodelling in mouse and human hearts	CARMEN plays a crucial role in the differentiation of cardiac precursor cells into cardiomyocytes in humans and mice	[103]
CASC7	↑ (all HF, in both PBMC and plasma)	PBMC and plasma	HFpEF, *n* = 62 HFrEF, *n* = 62	Healthy, *n* = 62	AF: overexpression repressed miR-30c in rat H9c2 cells, inhibiting pro-fibrotic cytokine IL-11		[104]
						Reduced myocardial apoptosis in rats by regulating miR-21	[105]
CHAST	↑	Whole blood	AMI, *n* = 53	Non-AMI, *n* = 90			[106]
						Repressed autophagy regulator Plekhm1, preventing cardiomyocyte autophagy and facilitating hypertrophy in TAC mice	[107]
FENDRR	↑ (compared to healthy, but not EH, no HFpEF)	PBMC	EH + HFpEF, *n* = 55	EH, no HFpEF, *n* = 23 Healthy, *n* = 25			[102]
					PF: silencing FENDRR reduced fibrosis via miR-106b/ Smad3 pathway in TAC mice		[108]
						Cardioprotective, as overexpression mitigated H_2_O_2_-induced damage in H9c2 rat cardiomyocytes	[109]
H19	↑	PBMC	AMI, *n* = 132	Healthy, *n* = 104			[110]
	↑ (all idiopathic PAH; discovery and validation cohorts)	Plasma	Idiopathic PAH, *n* = 52	Controls with normal RV functions, *n* = 57			[111]
	↑	Plasma	CAD, *n* = 300	Controls with normal coronary arteries, *n* = 180			[112]
					PF: upregulation enhances the synthesis of ECM proteins by inhibiting miR-29b-3p/ VEGFA/ TGF-β and DUSP5/ERK1/2 axis in rats		[59]
						H19-miR-675 axis targeting CaMKIIδ in mice acted as a negative regulator of cardiac hypertrophy	[113]
HEAT2	↑ (HFrEF + DCM and HFrEF + ICM)	Whole blood	HFrEF + DCM, *n* = 6 HFrEF + ICM, *n* = 10	Healthy, *n* = 8		HEAT2 expression levels had the power to predict the presence of HFpEF and mortality of HF patients	[92]
	↑	Whole blood	HFrEF, *n* = 69	Individuals without obvious risk of developing HF, *n* = 38			[92]
HOTAIR	↓	PBMC	Non-end-stage IDC HF, *n* = 25	Healthy, *n* = 18			[96]
	↓	Plasma	AMI, *n* = 50	Healthy, *n* = 50			[93]
					PF: enhanced fibrosis by activating Wnt pathway in mice		[114]
						Improved cardiac function via miR-17-5p/RORA and miR-34a/SIRT1 axis in murine models	[115,116]
KCNQ1OT1	↑	Serum	Diabetic cardiomyopathy, *n* = 6	Healthy, *n* = 6	Silencing KCNQ1OT1 reduced pyroptosis and fibrosis via miR-214-3p/ caspase-1/ TGF-β1 pathway in mice		[117]
	↑	PBMC	CHD, *n* = 267	Unexplained chest pain, *n* = 50 Healthy, *n* = 50			[118]
						Promoted cardiomyocyte apoptosis by targeting FUS in HF mice	[119]
LIPCAR	↑	Plasma	Post MI with LV remodelling, *n* = 87	Post MI without LV remodelling, *n* = 139	Potentially PF: upregulated in humans during the late stages of post-MI remodelling		[120]
	↑	Plasma	HF (cardiovascular death), *n* = 99	HF (no cardiovascular death), *n* = 99			[120]
	↑ (increased in HF patients with higher NYHA class, impaired kidney function, and lower hemoglobin)	Plasma	CHF, *n* = 967	-			[121]
	↑	Whole blood	HF post AMI, *n* = 59	Non-HF post AMI, *n* = 68			[122]
	↑	Plasma exosomes	One year post MI with LV remodelling, *n* = 5	One year post MI without LV remodelling, *n* = 5			[123]
						Overexpression in human VSMCs promoted cell proliferation and migration and enhanced the expression levels of MMP2 and MMP9	[124]
MALAT1	↑	PBMC	AMI, *n* = 132	Healthy, *n* = 104			[110]
	↑	PBMC	AMI, *n* = 160	Angina pectoris, *n* = 50			[125]
					PF: MALAT1 knockdown attenuated cardiac fibrosis via miR-145 in mice Promoted nuclear translocation of YAP in diabetic cardiomyopathy, facilitating cardiac fibrosis in mice		[126,127]
						Promoted cardiomyocyte apoptosis after MI via sponging miR-144-3p in mice	[128]
MHRT	↑ (compared to healthy, but not EH, no HFpEF)	PBMC	EH + HFpEF, *n* = 55	EH, no HFpEF, *n* = 23 Healthy, n = 25			[102]
	↓	Plasma	CHF, *n* = 88	Healthy, *n* = 65			[129]
	↑	Plasma	HF, *n* = 72	Non-HF, *n* = 60			[130]
	↑	Plasma	AMI, *n* = 47	Healthy, *n* = 28		Inhibited apoptosis in rat cardiomyocytes	[131]
					AF: in pressure overload TAC mouse model by inhibiting Brg1 PF: overexpression promoted collagen production via miR-3185 in MI mice		[132,133]
MIAT	↓	PBMC	STEMI, *n* = 274	NSTEMI, *n* = 140			[134]
	↑	PBMC	AMI, *n* = 132	Healthy, *n* = 104			[110]
					PF: MIAT silencing reduced cardiac fibrosis and alleviated HF via the PI3K/Akt pathway in human CFs and rats		[135]
						Contributed to the increase in proinflammatory IL-17 expression in diabetic cardiomyopathy mice	[136]
NRF	↑	Whole blood	AMI with HF, *n* = 76	AMI without HF, *n* = 58			[137]
						NRF silencing diminished myocardial necrosis via miR-873/RIPK1-RIPK3 axis in murine cardiomyocytes	[138]
NRON	↑	Plasma	HF, *n* = 72	Non-HF, *n* = 60			[130]
	↓	Whole blood	AIS, *n* = 65	Healthy, *n* = 65			[139]
					AF: mitigated atrial fibrosis by suppressing rat M1 macrophages and promoting NFATc3 phosphorylation in rat atrial fibroblasts		[140,141]
						Contributed to the progression of cardiac hypertrophy in the mouse heart	[142]
PVT1	↑	Serum	CHF, *n* = 92	Healthy, *n* = 60			[143]
					PF: via miR-145/HCN1 and miR-128-3p/SP1/ TGF-β1 axis in human CFs		[144,145]
						Intensified murine cardiomyocyte apoptosis via miR-216/Ccnd3 signalling axis	[146]
SARRAH	↑	Serum	AF, *n* = 95	Non-AF, *n* = 66			[147]
						Overexpression in mice facilitated recovery following AMI	[148]
SRA1	↑	Plasma	CHF, *n* = 93	Healthy, *n* = 62			[149]
					PF: SRA1 facilitated the activation of rat cardiac myofibroblasts by downregulating miR-148b		[150]
						Reduced hypoxia-induced damage in rat H9c2 cardiomyocytes by modulating the PPARγ/NF-κB signalling pathway	[151]
TUG1	↑	Serum	Hypertensive with HFpEF, *n* = 80	Hypertensive, without HF, *n* = 80			[152]
	↑	Serum	CHF, *n* = 98	Non-CHF, *n* = 86			[153]
	↑	Plasma	AMI, *n* = 15	Healthy, *n* = 18			[154]
					PF: via CHI3L1/TUG1/ miR-495-3p/ ETS1 axis in mice and TUG1/ miR-29b-3p /TGF-β1 axis in human CFs		[58,155]
						Reduced cardiomyocyte apoptosis by regulating miR-9/KLF5 and miR-132-3p/HDAC3 in rodents	[156,157]
TUSC7 (LOC285194)	↓	PBMC	Non-end-stage IDC HF, *n* = 25	Healthy, *n* = 18			[96]
						Targeting LOC285194 promoted proliferation and inhibited apoptosis in human VSMCs	[158]
UCA1	↑	Serum	AF, *n* = 96	Non-AF, *n* = 67			[147]
	↓ (2–48 h after AMI)	Plasma	AMI, *n* = 49	Non-AMI, *n* = 15			[159]
					Potentially PF: promoted progression of liver fibrosis in mice via miR18a/Smad3/TGF-β1 pathway		[160]
						Facilitated mouse cardiomyocyte hypertrophy via miR-184/HOXA9 axis, and enhanced rat cardiomyocyte proliferation by suppressing the miR-128/SUZ12/P27 pathway	[161,162]

lncRNAs are sorted in alphabetical order. HF, heart failure; HFrEF, heart failure with reduced ejection fraction; HFpEF, heart failure with preserved ejection fraction; CHF, chronic heart failure; ACS, acute coronary syndrome; CAD, coronary artery disease; DCM, dilated cardiomyopathy; IDC, ischemic dilated cardiomyopathy; EH, essential hypertension; ECM, extracellular matrix; MI, myocardial infarction; AMI, acute myocardial infarction; AIS, atherosclerotic ischemic stroke; PAH, pulmonary arterial hypertension; STEMI, ST elevated myocardial infarction; NSTEMI, non-ST elevated myocardial infarction; ParAF, paroxysmal atrial fibrillation; PerAF, persistent atrial fibrillation; RV, right ventricle; T2DM, type 2 diabetes mellitus; PBMC, peripheral blood mononuclear cell; VSMC, vascular smooth muscle cell; ANRIL, Antisense Non-coding RNA in the INK4 Locus; BACE1-AS, Beta-Secretase-1 Antisense RNA; CARMEN, Cardiac Mesoderm Enhancer-associated Non-coding; CASC7, Cancer Susceptibility Candidate 7; CHAST, Cardiac Hypertrophy-Associated Transcript; FENDRR, FOXF1 Adjacent Non-coding Developmental Regulatory RNA; HEAT2, heart-disease-associated transcript 2; HOTAIR, HOX Transcript Antisense RNA; KCNQ1OT1, KCNQ1 overlapping transcript 1; LICAR, Long Intergenic non-coding RNA Predicting Cardiac Remodelling; MALAT1, Metastasis-associated lung adenocarcinoma transcript 1; MHRT, myosin heavy-chain-associated RNA; MIAT, MI-associated transcript; NRF, necrosis-related factor; NRON, non-coding repressor of NFAT; PVT1, plasmacytoma variant translocation 1; SARRAH, SCOT1-antisense RNA regulated during ageing in the heart; SRA1, steroid receptor RNA activator 1; TUG1, taurine upregulated 1; TUSC7, Tumor Suppressor Candidate 7; UCA1, urothelial carcinoma-associated 1; Akt, Protein kinase B; Brg1, ATP-dependent chromatin remodeler SMARCA4; CaMKIIδ, Ca^2+^/calmodulin dependent protein kinase II delta; Ccnd3, G1/S-specific cyclin-D3; CHI3L1, Chitinase-3-like protein 1; DUSP5, Dual specificity protein phosphatase 5; ERK1/2, Extracellular signal-regulated kinases; ETS1, protein C-ets-1; FUS, RNA-binding protein fused in sarcoma; HCN1, Potassium/sodium hyperpolarization-activated cyclic nucleotide-gated channel 1; HDAC3, Histone Deacetylase 3; HOXA9, Homeobox protein Hox-A9; Il-11, Interleukin-11; KLF5, Krüppel-like factor 5; MMP2, Matrix metallopeptidase 2; MMP9, Matrix metallopeptidase 9; NF-κB, Nuclear factor kappa-light-chain-enhancer of activated B cells; NFATc3, Nuclear factor of activated T-cells, cytoplasmic 3; P27, cyclin-dependent kinase inhibitor p27; PI3K, Phosphoinositide 3-kinases; Plekhm1, Pleckstrin homology domain-containing family M member 1; PPARγ, Peroxisome proliferator-activated receptor gamma; RIPK1, Receptor-interacting serine/threonine-protein kinase 1; RIPK3, Receptor-interacting serine/threonine-protein kinase 3; RORA, Retinoic Acid Receptor-Related Orphan Receptor Alpha; Smad3, mothers against decapentaplegic homologue 3; SIRT1, Sirtuin 1; SUZ12, Polycomb protein SUZ12; TAC, Transverse Aortic Constriction; TGF-β, Transforming growth factor beta; VEGFA, Vascular endothelial growth factor A; Wnt, Wingless and Int-1; YAP, Yes-associated protein 1; PF, pro-fibrotic; AF, anti-fibrotic; ↑, upregulation; and ↓, downregulation.

**Table 3:** circRNAs as potential biomarkers of HF and their role in fibrosis progression.

circRNA	Change in Expression	Sample Type (Human)	Patient Experimental Group	Patient Control Group	Fibrosis	Other Functions	Study
circDEPC5 (hsa_circ_0062960)	↑	Plasma	HFrEF, *n* = 30	Healthy, *n* = 30			[171]
						Host gene Depc5 knockout is linked with vascular defects in mice	[172]
circHECW2 (hsa_circ_0118464)	↑	Epicardial adipose tissue	HFpEF, *n* = 5	Non-HF control, *n* = 5			[173]
(hsa_circ_0057576)	↑	Plasma	CAD, *n* = 3	Healthy, *n* = 3	PF: targets AF miR-130a-3p in humans		[174]
(mmu_Hecw2_0009)					PF: promoted fibrosis and hypertrophy in mice		[175]
						Host gene HECW2 variant is linked with congenital long QT syndrome	[176]
circSMARCA5 (hsa_circ_0001445)	↓	Plasma	CAD, *n* = 200	--	circSMARCA5 is reduced in atherosclerosis	circSMARCA5 was stable in blood plasma after 72 h of storing at room temperature	[177]
(hsa_circ_0001445)	↓	Peripheral blood leukocytes	CHD, *n* = 94	Healthy, *n* = 126			[178]
circAMOTL1	↑ (Young patients)	Heart tissues	Tissue mix from 3 patients under one year old, *n* = 1	Tissue mix from 3 elderly patients, *n* = 1			[179]
					PF: interacted with EIF4A3 and stabilized MARCKS in diabetic cardiomyopathy mice	Silencing circAmotl1 decreased cell proliferation and levels of reactive oxygen species (ROS) in vitro	[180]
circFOXO3	↑ (Humans > 50 years old)	Heart tissues	Humans > 50 years old, *n* = 11	Humans < 50 years old, *n* = 9	PF: reduced activity of anti-stress proteins HIF1α, FAK, and E2F1 in mouse embryonic fibroblasts		[181]
						Promoted senescence, alleviated I/R injury	[182]
circZNF609 (hsa_circ_0000615)	↓	Peripheral blood leukocytes	CAD, *n* = 330	Healthy, *n* = 209		Overexpression in mouse macrophages is anti-inflammatory (decreases IL-6 and TNF-α, increases IL-10)	[183]
MICRA (hsa_circ_0000615)	↓	Peripheral blood	AMI, *n* = 642	Healthy, *n* = 86			[184]
	↓ (AMI with rEF)	Whole blood	AMI, rEF ≤ 40%, *n* = 87 mrEF = 41–49%, *n* = 106 pEF ≥ 50%, *n* = 279	—			[166]
					Potentially AF: reduced lung fibrosis in mice via miR-145-5p/KLF4 axis		[185]
circBPTF (hsa_circ_0000799)	↑ (DCM and ICM)	LV tissue	DCM, *n* = 26 ICM, *n* = 17	Non-failing heart donor, *n* = 23			[186]
(hsa_circ_0000799)	↑	LV tissue	Non-end-stage IHF, *n* = 12	Healthy, *n* = 12			[165]
(hsa_circ_0000799)	↑	LV tissue	End-stage IHF, *n* = 36	Healthy, *n* = 44			[165]
(hsa_circ_0045462)					Potentially PF: sponges miR-486-5p (AF in heart) in human arterial smooth muscle cell		[187,188]
						Knockdown of circBPTF reduced inflammation and oxidative stress in HUVEC cells via miR-384/LIN28B pathway	[189]
circPRDM5 (hsa_circ_0005654)	↑ (DCM and ICM)	LV tissue	DCM, *n* = 26 ICM, *n* = 17	Non-failing heart donor, *n* = 23			[186]
(hsa_circ_0005654)	↓	Serum	AMI, *n* = 118	Healthy, *n* = 60		Potential biomarker for dynamic post-surgery monitoring	[190]
(hsa_circ_0070820)					PF: in human lens epithelial cells		[191]
circFNDC3B (hsa_circ_0006156)	↑ (DCM and ICM)	LV tissue	DCM, *n* = 26 ICM, *n* = 17	Non-failing heart donor, *n* = 23			[186]
					AF: AAV9-driven circFNDRC3B overexpression attenuates fibrosis following MI in mice		[192]
						circFNDRC3B in mouse cardiac endothelial cells improved endothelial function and protected cardiomyocytes from death	[193]
circHIPK3 (circR-284)	↑ (circR-284 to miR-221 ratio)	Serum	Urgent ICD, *n* = 41 Symptomatic ICD, *n* = 24	Asymptomatic disease, *n* = 47			[194]
(hsa_circ_0000284)	↑	Plasma exosomes	Chronic coronary syndrome, *n* = 135	Non-cardiac chest pain, *n* = 83			[195]
(hsa_circ_0000284)	↑	Peripheral blood	Hypertension, *n* = 100 Prehypertension, *n* = 100	Non-hypertensive, *n* = 100			[196]
						Inhibited development of atherosclerosis, myocardial injury, and MI, but also contributed to cardiomyopathy, myocardial fibrosis, and HF	[197]
(mmu_circ_0001052)					AF: silencing circHIPK3 in mice alleviated cardiac fibrosis in vitro and in vivo by releasing AF miR-29b-3p		[198]
circNFIB (hsa_circ_0086376)	↓	Epicardial adipose tissue	CAD with HF, *n* = 5	CAD, non-HF, *n* = 5			[199]
(mmu_circ_0011794)					AF: upregulation in mouse fibroblasts reduced cardiac fibrosis by sponging miR-433		[200]
						SO_2_ enhanced circNFIB expression in neonatal rat CFs, which in turn suppressed the Wnt/β-catenin and p38 MAPK pathways, mitigating cardiac fibrosis	[201]
circCDR1as	↑	Plasma	CHF, *n* = 30	Healthy, *n* = 30			[202]
(mmu_circ_001946)					AF: AAV9 circCDR1as administration in vivo (mice) improved %EF and decreased fibrotic area at 3 and 4 weeks post MI		[203]
						circCDR1as levels positively correlated with EF% and LV stroke volume, and negatively correlated with infarct size in heart of post-MI HF pigs, treated with AF agent bufalin	[204]
circC12ORF51 (hsa_circ_0097435)	↑	Peripheral blood cells and plasma exosomes	HF, *n* = 40	Healthy, *n* = 40		circC12ORF51 silencing in AC16 human cardiomyocytes inhibited myocardial apoptosis, while hsa_circ_0097435 overexpression promoted cardiomyocyte apoptosis	[205]
circLAS1L (hsa_circ_0090876)	↓	Whole blood	AMI, *n* = 30	Healthy, *n* = 30	AF: inhibited expression of pro-fibrotic markers in human CFs: α-SMA, collagen I, collagen III	Overexpression inhibited human fibroblast proliferation and migration, as well as promoted apoptosis	[206]
circDNAJC6 circTMEM56 circMBOAT2	↓	Serum	HCM, *n* = 64, among which obstructive HCM, *n* = 31	Healthy, *n* = 53		circTMEM56 and circDNAJC6 negatively correlated with echocardiographic parameters in HCM	[207]
MFACR	↑	Plasma	AMI, *n* = 61	Healthy, *n* = 61			[208]
(mm9_circ_016597)						Regulated mitochondrial fission and apoptosis by targeting miR-652-3p in mice cardiomyocytes	[209]
circROBO2 (hsa_circ_0124644)	↑	Peripheral blood	CAD, *n* = 137	Healthy, *n* = 115			[210]
						circROBO2 knockdown in mice reduced cardiomyocyte apoptosis by upregulating miR-1184	[211]
circMYO9A (hsa_circ_0036176)	↑	Myocardial tissue	HF, *n* = 24	Healthy donor, *n* = 18	AF: suppressed human cardiac fibroblast proliferation via Myo9a-208	circMYO9A encodes 208 amino acids length protein Myo9a-208	[212]

HF, heart failure; HFrEF, heart failure with reduced ejection fraction; HFpEF, heart failure with preserved ejection fraction; mrEF, mid-range ejection fraction; CHF, chronic heart failure; IHF, ischemic heart failure; CAD, coronary artery disease; DCM, dilated cardiomyopathy; HCM, hypertrophic cardiomyopathy; ICM, ischemic cardiomyopathy; MI, myocardial infarction; ICD, ischemic cerebrovascular disease; I/R, ischemia/reperfusion; PBMC, peripheral blood mononuclear cell; VSMC, vascular smooth muscle cell; AMOTL1, Angiomotin-like protein 1; BPTF, Bromodomain PHD Finger Transcription Factor; DEPC5, DEP Domain Containing 5, GATOR1 Subcomplex Subunit; DNAJC6, DnaJ heat shock protein family (Hsp40) member C6; EIF4A3, Eukaryotic Translation Initiation Factor 4A3; FAK, Focal Adhesion Kinase; FNDC3B, Fibronectin Type III Domain Containing 3B; FOXO3, Forkhead Box O3; HECW2, HECT, C2 And WW Domain Containing E3 Ubiquitin Protein Ligase 2; HIF1α, Hypoxia-Inducible Factor 1 Alpha; HIPK3, Homeodomain-Interacting Protein Kinase 3; IL-10, Interleukin-10; IL-6, Interleukin-6; KLF4, Krüppel-Like Factor 4; LAS1L, LAS1-Like Ribosome Biogenesis Factor; LIN28B, LIN-28 homologue B; MAPK, Mitogen-Activated Protein Kinase; MARCKS, Myristoylated Alanine-Rich C Kinase Substrate; MBOAT2, Membrane-Bound O-Acyltransferase Domain Containing 2; MFACR, Mitochondrial fission and apoptosis-related circRNA; MICRA, Myocardial Infarction-associated Long Non-Coding RNA; MYO9A, Myosin IXA; NFIB, Nuclear factor I B; p38, p38 Mitogen-Activated Protein Kinase; PDM5, Polycomb Domain Protein 5; ROBO2, Roundabout Guidance Receptor 2; SMARCA5, SWI/SNF-Related, Matrix-Associated, Actin-Dependent Regulator of Chromatin Subfamily A Member 5; TMEM56, Transmembrane Protein 56; TNF-α, Tumor necrosis factor alpha; Wnt, Wingless and Int-1; ZNF609, Zinc Finger Protein 609; PF, pro-fibrotic; AF, anti-fibrotic; ↑, upregulation; and ↓, downregulation.

## Data Availability

No new data were created or analyzed in this study.

## References

[1] Khan M.S., Shahid I., Bennis A., Rakisheva A., Metra M., Butler J. (2024). Global Epidemiology of Heart Failure. Nat. Rev. Cardiol..

[2] Bozkurt B., Coats A.J.S., Tsutsui H., Abdelhamid C.M., Adamopoulos S., Albert N., Anker S.D., Atherton J., Böhm M., Butler J. (2021). Universal Definition and Classification of Heart Failure: A Report of the Heart Failure Society of America, Heart Failure Association of the European Society of Cardiology, Japanese Heart Failure Society and Writing Committee of the Universal Definition of Heart Failure: Endorsed by the Canadian Heart Failure Society, Heart Failure Association of India, Cardiac Society of Australia and New Zealand, and Chinese Heart Failure Association. Eur. J. Heart Fail..

[3] Arrigo M., Jessup M., Mullens W., Reza N., Shah A.M., Sliwa K., Mebazaa A. (2020). Acute Heart Failure. Nat. Rev. Dis. Primers.

[4] Shah S.J., Borlaug B.A., Kitzman D.W., McCulloch A.D., Blaxall B.C., Agarwal R., Chirinos J.A., Collins S., Deo R.C., Gladwin M.T. (2020). Research Priorities for Heart Failure with Preserved Ejection Fraction: National Heart, Lung, and Blood Institute Working Group Summary. Circulation.

[5] Ziaeian B., Fonarow G.C. (2016). Epidemiology and Aetiology of Heart Failure. Nat. Rev. Cardiol..

[6] Tikhomirov R., Donnell B.R.O., Catapano F., Faggian G., Gorelik J., Martelli F., Emanueli C. (2020). Exosomes: From Potential Culprits to New Therapeutic Promise in the Setting of Cardiac Fibrosis. Cells.

[7] Bayés-Genís A., Aimo A., Metra M., Anker S., Seferovic P., Rapezzi C., Castiglione V., Núñez J., Emdin M., Rosano G. (2022). Head-to-Head Comparison between Recommendations by the ESC and ACC/AHA/HFSA Heart Failure Guidelines. Eur. J. Heart Fail..

[8] Abovich A., Matasic D.S., Cardoso R., Ndumele C.E., Blumenthal R.S., Blankstein R., Gulati M. (2023). The AHA/ACC/HFSA 2022 Heart Failure Guidelines: Changing the Focus to Heart Failure Prevention. Am. J. Prev. Cardiol..

[9] Frisk M., Le C., Shen X., Røe Å.T., Hou Y., Manfra O., Silva G.J.J., van Hout I., Norden E.S., Aronsen J.M. (2021). Etiology-Dependent Impairment of Diastolic Cardiomyocyte Calcium Homeostasis in Heart Failure with Preserved Ejection Fraction. J. Am. Coll. Cardiol..

[10] Robinson E.L., Baker A.H., Brittan M., Mccracken I., Condorelli G., Emanueli C., Srivastava P.K., Gaetano C., Thum T., Vanhaverbeke M. (2022). Dissecting the Transcriptome in Cardiovascular Disease. Cardiovasc. Res..

[11] Tham Y.K., Bernardo B.C., Ooi J.Y.Y., Weeks K.L., McMullen J.R. (2015). Pathophysiology of Cardiac Hypertrophy and Heart Failure: Signaling Pathways and Novel Therapeutic Targets. Arch. Toxicol..

[12] Ma Z.G., Yuan Y.P., Wu H.M., Zhang X., Tang Q.Z. (2018). Cardiac Fibrosis: New Insights into the Pathogenesis. Int. J. Biol. Sci..

[13] Maruyama K., Imanaka-Yoshida K. (2022). The Pathogenesis of Cardiac Fibrosis: A Review of Recent Progress. Int. J. Mol. Sci..

[14] Xia P., Liu Y., Cheng Z. (2016). Signaling Pathways in Cardiac Myocyte Apoptosis. Biomed. Res. Int..

[15] Konstam M.A., Kramer D.G., Patel A.R., Maron M.S., Udelson J.E. (2011). Left Ventricular Remodeling in Heart Failure: Current Concepts in Clinical Significance and Assessment. JACC Cardiovasc. Imaging.

[16] Azevedo P.S., Polegato B.F., Minicucci M.F., Paiva S.A.R., Zornoff L.A.M. (2016). Cardiac Remodeling: Concepts, Clinical Impact, Pathophysiological Mechanisms and Pharmacologic Treatment. Arq. Bras. Cardiol..

[17] Jiang W., Xiong Y., Li X., Yang Y. (2021). Cardiac Fibrosis: Cellular Effectors, Molecular Pathways, and Exosomal Roles. Front. Cardiovasc. Med..

[18] Simmonds S.J., Cuijpers I., Heymans S., Jones E.A.V. (2020). Cellular and Molecular Differences between HFpEF and HFrEF: A Step Ahead in an Improved Pathological Understanding. Cells.

[19] Shah S.J., Kitzman D.W., Borlaug B.A., Van Heerebeek L., Zile M.R., Kass D.A., Paulus W.J. (2016). Phenotype-Specific Treatment of Heart Failure with Preserved Ejection Fraction. Circulation.

[20] Crossman D.J., Shen X., Jüllig M., Munro M., Hou Y., Middleditch M., Shrestha D., Li A., Lal S., Dos Remedios C.G. (2017). Increased Collagen within the Transverse Tubules in Human Heart Failure. Cardiovasc. Res..

[21] Mukherjee D., Sen S. (1991). Alteration of Collagen Phenotypes in Ischemic Cardiomyopathy. J. Clin. Investig..

[22] Whittaker P., Boughner D.R., Kloner R.A. (1989). Analysis of Healing After Myocardial Infarction Using Polarized Light Microscopy. Am. J. Pathol..

[23] Naugle J.E., Olson E.R., Zhang X., Mase S.E., Pilati C.F., Maron M.B., Folkesson H.G., Horne W.I., Doane K.J., Gary Meszaros J. (2006). Type VI Collagen Induces Cardiac Myofibroblast Differentiation: Implications for Postinfarction Remodeling. Am. J. Physiol. Heart Circ. Physiol..

[24] Spinale F.G., Coker M.L., Heung L.J., Bond B.R., Gunasinghe H.R., Etoh T., Goldberg A.T., Zellner J.L., Crumbley A.J. (2000). A Matrix Metalloproteinase Induction/Activation System Exists in the Human Left Ventricular Myocardium and Is Upregulated in Heart Failure. Circulation.

[25] Schafer S., Viswanathan S., Widjaja A.A., Lim W.W., Moreno-Moral A., DeLaughter D.M., Ng B., Patone G., Chow K., Khin E. (2017). IL-11 Is a Crucial Determinant of Cardiovascular Fibrosis. Nature.

[26] Sweeney M., O’Fee K., Villanueva-Hayes C., Rahman E., Lee M., Vanezis K., Andrew I., Lim W.W., Widjaja A., Barton P.J.R. (2023). Cardiomyocyte-Restricted Expression of IL11 Causes Cardiac Fibrosis, Inflammation, and Dysfunction. Int. J. Mol. Sci..

[27] Xue Y., Clopton P., Peacock W.F., Maisel A.S. (2011). Serial Changes in High-Sensitive Troponin i Predict Outcome in Patients with Decompensated Heart Failure. Eur. J. Heart Fail..

[28] Nadar S.K., Shaikh M.M. (2019). Biomarkers in Routine Heart Failure Clinical Care. Card. Fail. Rev..

[29] Inamdar A.A., Inamdar A.C. (2016). Heart Failure: Diagnosis, Management and Utilization. J. Clin. Med..

[30] Kumari S., Sharma U., Jindal D., Basak T. (2023). A Narrative Review on Serum Biomarkers of Cardiac Fibrosis. J. Pract. Cardiovasc. Sci..

[31] Frangogiannis N.G. (2021). Cardiac Fibrosis. Cardiovasc. Res..

[32] Nemeth K., Bayraktar R., Ferracin M., Calin G.A. (2024). Non-Coding RNAs in Disease: From Mechanisms to Therapeutics. Nat. Rev. Genet..

[33] Caporali A., Anwar M., Devaux Y., Katare R., Martelli F., Srivastava P.K., Pedrazzini T., Emanueli C. (2024). Non-Coding RNAs as Therapeutic Targets and Biomarkers in Ischaemic Heart Disease. Nat. Rev. Cardiol..

[34] Jalink E.A., Schonk A.W., Boon R.A., Juni R.P. (2023). Non-Coding RNAs in the Pathophysiology of Heart Failure with Preserved Ejection Fraction. Front. Cardiovasc. Med..

[35] Shang R., Lee S., Senavirathne G., Lai E.C. (2023). MicroRNAs in Action: Biogenesis, Function and Regulation. Nat. Rev. Genet..

[36] Quah S., Subramanian G., Tan J.S.L., Utami K.H., Sampath P. (2024). MicroRNAs: A Symphony Orchestrating Evolution and Disease Dynamics. Trends Mol. Med..

[37] Hou B., Yu D., Bai H., Du X. (2024). Research Progress of MiRNA in Heart Failure: Prediction and Treatment. J. Cardiovasc. Pharmacol..

[38] Wong L.L., Zou R., Zhou L., Lim J.Y., Phua D.C.Y., Liu C., Chong J.P.C., Ng J.Y.X., Liew O.W., Chan S.P. (2019). Combining Circulating MicroRNA and NT-ProBNP to Detect and Categorize Heart Failure Subtypes. J. Am. Coll. Cardiol..

[39] Vilella-Figuerola A., Gallinat A., Escate R., Mirabet S., Padró T., Badimon L. (2022). Systems Biology in Chronic Heart Failure—Identification of Potential MiRNA Regulators. Int. J. Mol. Sci..

[40] Navickas R., Gal D., Laucevičius A., Taparauskaite A., Zdanyte M., Holvoet P. (2016). Identifying Circulating MicroRNAs as Biomarkers of Cardiovascular Disease: A Systematic Review. Cardiovasc. Res..

[41] Goren Y., Kushnir M., Zafrir B., Tabak S., Lewis B.S., Amir O. (2012). Serum Levels of MicroRNAs in Patients with Heart Failure. Eur. J. Heart Fail..

[42] Voellenkle C., van Rooij J., Cappuzzello C., Greco S., Arcelli D., Di Vito L., Melillo G., Rigolini R., Costa E., Crea F. (2010). MicroRNA Signatures in Peripheral Blood Mononuclear Cells of Chronic Heart Failure Patients. Physiol. Genom..

[43] Watson C.J., Gupta S.K., O’Connell E., Thum S., Glezeva N., Fendrich J., Gallagher J., Ledwidge M., Grote-Levi L., McDonald K. (2015). MicroRNA Signatures Differentiate Preserved from Reduced Ejection Fraction Heart Failure. Eur. J. Heart Fail..

[44] Ovchinnikova E.S., Schmitter D., Vegter E.L., Ter Maaten J.M., Valente M.A.E., Liu L.C.Y., Van Der Harst P., Pinto Y.M., De Boer R.A., Meyer S. (2016). Signature of Circulating MicroRNAs in Patients with Acute Heart Failure. Eur. J. Heart Fail..

[45] Marques F.Z., Vizi D., Khammy O., Mariani J.A., Kaye D.M. (2016). The Transcardiac Gradient of Cardio-MicroRNAs in the Failing Heart. Eur. J. Heart Fail..

[46] Wong L.L., Armugam A., Sepramaniam S., Karolina D.S., Lim K.Y., Lim J.Y., Chong J.P.C., Ng J.Y.X., Chen Y.T., Chan M.M.Y. (2015). Circulating MicroRNAs in Heart Failure with Reduced and Preserved Left Ventricular Ejection Fraction. Eur. J. Heart Fail..

[47] Galluzzo A., Gallo S., Pardini B., Birolo G., Fariselli P., Boretto P., Vitacolonna A., Peraldo-Neia C., Spilinga M., Volpe A. (2021). Identification of Novel Circulating MicroRNAs in Advanced Heart Failure by Next-Generation Sequencing. ESC Heart Fail..

[48] Cakmak H.A., Coskunpinar E., Ikitimur B., Barman H.A., Karadag B., Tiryakioglu N.O., Kahraman K., Vural V.A. (2015). The Prognostic Value of Circulating MicroRNAs in Heart Failure: Preliminary Results from a Genome-Wide Expression Study. J. Cardiovasc. Med..

[49] van den Berg N.W.E., Kawasaki M., Nariswari F.A., Fabrizi B., Neefs J., van der Made I., Wesselink R., van Boven W.J.P., Driessen A.H.G., Jongejan A. (2023). MicroRNAs in Atrial Fibrillation Target Genes in Structural Remodelling. Cell Tissue Res..

[50] Paim L.R., da Silva L.M., Antunes-Correa L.M., Ribeiro V.C., Schreiber R., Minin E.O.Z., Bueno L.C.M., Lopes E.C.P., Yamaguti R., Coy-Canguçu A. (2024). Profile of Serum MicroRNAs in Heart Failure with Reduced and Preserved Ejection Fraction: Correlation with Myocardial Remodeling. Heliyon.

[51] Fang L., Ellims A.H., Moore X.l., White D.A., Taylor A.J., Chin-Dusting J., Dart A.M. (2015). Circulating MicroRNAs as Biomarkers for Diffuse Myocardial Fibrosis in Patients with Hypertrophic Cardiomyopathy. J. Transl. Med..

[52] Zhang Y., Yuan B., Xu Y., Zhou N., Zhang R., Lu L., Feng Z. (2022). MiR-208b/MiR-21 Promotes the Progression of Cardiac Fibrosis Through the Activation of the TGF-Β1/Smad-3 Signaling Pathway: An in Vitro and in Vivo Study. Front. Cardiovasc. Med..

[53] Marfella R., Di Filippo C., Potenza N., Sardu C., Rizzo M.R., Siniscalchi M., Musacchio E., Barbieri M., Mauro C., Mosca N. (2013). Circulating microRNA Changes in Heart Failure Patients Treated with Cardiac Resynchronization Therapy: Responders vs. Non-Responders. Eur. J. Heart Fail..

[54] Cui H., Banerjee S., Xie N., Ge J., Liu R.M., Matalon S., Thannickal V.J., Liu G. (2016). MicroRNA-27a-3p Is a Negative Regulator of Lung Fibrosis by Targeting Myofibroblast Differentiation. Am. J. Respir. Cell. Mol. Biol..

[55] Cheng Q., Chen M., Wang H., Chen X., Wu H., Du Y., Xue J. (2022). MicroRNA-27a-3p Inhibits Lung and Skin Fibrosis of Systemic Sclerosis by Negatively Regulating SPP1. Genomics.

[56] Li D., Shen M., Deng X., Bai Y. (2022). MicroRNA MiR-27a-3p Accelerates Cardiac Hypertrophy by Targeting Neuro-Oncological Ventral Antigen 1. Bioengineered.

[57] Liang J.N., Zou X., Fang X.H., Xu J.D., Xiao Z., Zhu J.N., Li H., Yang J., Zeng N., Yuan S.J. (2019). The Smad3-MiR-29b/MiR-29c Axis Mediates the Protective Effect of Macrophage Migration Inhibitory Factor against Cardiac Fibrosis. Biochim. Biophys. Acta Mol. Basis Dis..

[58] Guo Y., Sun Z., Chen M., Lun J. (2021). LncRNA TUG1 Regulates Proliferation of Cardiac Fibroblast via the MiR-29b-3p/TGF-Β1 Axis. Front. Cardiovasc. Med..

[59] Guo F., Tang C., Huang B., Gu L., Zhou J., Mo Z., Liu C., Liu Y. (2022). LncRNA H19 Drives Proliferation of Cardiac Fibroblasts and Collagen Production via Suppression of the MiR-29a-3p/MiR-29b-3pVEGFA/TGF-β Axis. Mol. Cells.

[60] Gao L., Qiu F., Cao H., Li H., Dai G., Ma T., Gong Y., Luo W., Zhu D., Qiu Z. (2023). Therapeutic Delivery of MicroRNA-125a-5p Oligonucleotides Improves Recovery from Myocardial Ischemia/Reperfusion Injury in Mice and Swine. Theranostics.

[61] Dong M., Liao J.K., Fang F., Lee A.P.W., Yan B.P.Y., Liu M., Yu C.M. (2012). Increased Rho Kinase Activity in Congestive Heart Failure. Eur. J. Heart Fail..

[62] Wang Q., Wei S., Li L., Bu Q., Zhou H., Su W., Liu Z., Wang M., Lu L. (2021). MiR-139-5p Sponged by LncRNA NEAT1 Regulates Liver Fibrosis via Targeting β-Catenin/SOX9/TGF-Β1 Pathway. Cell Death Discov..

[63] Ahn S.H., Kim H., Lee I., Lee J.H., Cho S., Choi Y.S. (2021). MicroRNA-139-5p Regulates Fibrotic Potentials via Modulation of Collagen Type 1 and Phosphorylated P38 MAPK in Uterine Leiomyoma. Yonsei Med. J..

[64] Li Y., Zhao K., Hu Y., Yang F., Li P., Liu Y. (2024). MicroRNA-142-3p Alleviated High Salt-Induced Cardiac Fibrosis via Downregulating Optineurin-Mediated Mitophagy. iScience.

[65] Su Q., Liu Y., Lv X.W., Ye Z.L., Sun Y.H., Kong B.H., Qin Z.B. (2019). Inhibition of LncRNA TUG1 Upregulates MiR-142-3p to Ameliorate Myocardial Injury during Ischemia and Reperfusion via Targeting HMGB1- and Rac1-Induced Autophagy. J. Mol. Cell. Cardiol..

[66] Scrutinio D., Conserva F., Passantino A., Iacoviello M., Lagioia R., Gesualdo L. (2017). Circulating MicroRNA-150-5p as a Novel Biomarker for Advanced Heart Failure: A Genome-Wide Prospective Study. J. Heart Lung Transplant..

[67] Shen J., Xing W., Gong F., Wang W., Yan Y., Zhang Y., Xie C., Fu S. (2019). MiR-150-5p Retards the Progression of Myocardial Fibrosis by Targeting EGR1. Cell Cycle.

[68] Deng P., Chen L., Liu Z., Ye P., Wang S., Wu J., Yao Y., Sun Y., Huang X., Ren L. (2016). MicroRNA-150 Inhibits the Activation of Cardiac Fibroblasts by Regulating c-Myb. Cell. Physiol. Biochem..

[69] Lai Y.J., Tsai F.C., Chang G.J., Chang S.H., Huang C.C., Chen W.J., Yeh Y.H. (2022). MiR-181b Targets Semaphorin 3A to Mediate TGF-β–Induced Endothelial-Mesenchymal Transition Related to Atrial Fibrillation. J. Clin. Investig..

[70] Yang H., Shan L., Gao Y., Li L., Xu G., Wang B., Yin X., Gao C., Liu J., Yang W. (2021). MicroRNA-181b Serves as a Circulating Biomarker and Regulates Inflammation in Heart Failure. Dis. Markers.

[71] Fang F., Zhang X., Li B., Gan S. (2022). MiR-182-5p Combined with Brain-Derived Neurotrophic Factor Assists the Diagnosis of Chronic Heart Failure and Predicts a Poor Prognosis. J. Cardiothorac. Surg..

[72] Chen Y., Zhang Q., Zhou Y., Yang Z., Tan M. (2020). Inhibition of MiR-182-5p Attenuates Pulmonary Fibrosis via TGF-β/Smad Pathway. Hum. Exp. Toxicol..

[73] Pei G., Chen L., Wang Y., He C., Fu C., Wei Q. (2023). Role of MiR-182 in Cardiovascular and Cerebrovascular Diseases. Front. Cell Dev. Biol..

[74] He F., Lv P., Zhao X., Wang X., Ma X., Meng W., Meng X., Dong S. (2014). Predictive Value of Circulating MiR-328 and MiR-134 for Acute Myocardial Infarction. Mol. Cell. Biochem..

[75] Zhao D., Li C., Yan H., Li T., Qian M., Zheng N., Jiang H., Liu L., Xu B., Wu Q. (2018). Cardiomyocyte Derived MiR-328 Promotes Cardiac Fibrosis by Paracrinely Regulating Adjacent Fibroblasts. Cell. Physiol. Biochem..

[76] Huang J., Wang F., Sun X., Chu X., Jiang R., Wang Y., Pang L. (2021). Myocardial Infarction Cardiomyocytes-Derived Exosomal MiR-328-3p Promote Apoptosis via Caspase Signaling. Am. J. Transl. Res..

[77] Wang Y., Huang C., Reddy Chintagari N., Bhaskaran M., Weng T., Guo Y., Xiao X., Liu L. (2013). MiR-375 Regulates Rat Alveolar Epithelial Cell Trans-Differentiation by Inhibiting Wnt/β-Catenin Pathway. Nucleic Acids Res..

[78] Ali Sheikh M.S. (2020). Overexpression of MiR-375 Protects Cardiomyocyte Injury Following Hypoxic-Reoxygenation Injury. Oxid. Med. Cell. Longev..

[79] Tijsen A.J., Creemers E.E., Moerland P.D., De Windt L.J., Van Der Wal A.C., Kok W.E., Pinto Y.M. (2010). MiR423-5p as a Circulating Biomarker for Heart Failure. Circ. Res..

[80] Chen Y., Li X., Li Y., Wu Y., Huang G., Wang X., Guo S. (2022). Downregulation of MicroRNA-423-5p Suppresses TGF-Β1-Induced EMT by Targeting FOXP4 in Airway Fibrosis. Mol. Med. Rep..

[81] Zhu X., Lu X. (2019). MiR-423-5p Inhibition Alleviates Cardiomyocyte Apoptosis and Mitochondrial Dysfunction Caused by Hypoxia/Reoxygenation through Activation of the Wnt/β-Catenin Signaling Pathway via Targeting MYBL2. J. Cell. Physiol..

[82] Chen X., Shi C., Wang C., Liu W., Chu Y., Xiang Z., Hu K., Dong P., Han X. (2017). The Role of MiR-497-5p in Myofibroblast Differentiation of LR-MSCs and Pulmonary Fibrogenesis. Sci. Rep..

[83] Tikhomirov R., Reilly-O’Donnell B., Lucarelli C., Srivastava P.K., Anwar M., Yiu C.H.K., Dielesen J., Piella S.N., Kwan Z., Zaccagnini G. (2023). Interleukin 11-Induced MicroRNAs as Functional Mediators and Circulating Biomarkers of Cardiac Fibrosis. bioRxiv.

[84] Mattick J.S., Amaral P.P., Carninci P., Carpenter S., Chang H.Y., Chen L.L., Chen R., Dean C., Dinger M.E., Fitzgerald K.A. (2023). Long Non-Coding RNAs: Definitions, Functions, Challenges and Recommendations. Nat. Rev. Mol. Cell Biol..

[85] Uszczynska-Ratajczak B., Lagarde J., Frankish A., Guigó R., Johnson R. (2018). Towards a Complete Map of the Human Long Non-Coding RNA Transcriptome. Nat. Rev. Genet..

[86] Zhang X., Wang W., Zhu W., Dong J., Cheng Y., Yin Z., Shen F. (2019). Mechanisms and Functions of Long Non-Coding RNAs at Multiple Regulatory Levels. Int. J. Mol. Sci..

[87] Nie X., Fan J., Wang D.W. (2023). The Function and Therapeutic Potential of LncRNAs in Cardiac Fibrosis. Biology.

[88] Shen S., Jiang H., Bei Y., Xiao J., Li X. (2017). Long Non-Coding RNAs in Cardiac Remodeling. Cell. Physiol. Biochem..

[89] Uchida S., Dimmeler S. (2015). Long Noncoding RNAs in Cardiovascular Diseases. Circ. Res..

[90] Qu X., Du Y., Shu Y., Gao M., Sun F., Luo S., Yang T., Zhan L., Yuan Y., Chu W. (2017). MIAT Is a Pro-Fibrotic Long Non-Coding RNA Governing Cardiac Fibrosis in Post-Infarct Myocardium. Sci. Rep..

[91] Wylezinski L.S., Shaginurova G.I., Spurlock C.F. (2020). Longitudinal Assessment and Stability of Long Non-Coding RNA Gene Expression Profiles Measured in Human Peripheral Whole Blood Collected into PAXgene Blood RNA Tubes. BMC Res. Notes.

[92] Boeckel J.N., Perret M.F., Glaser S.F., Seeger T., Heumüller A.W., Chen W., John D., Kokot K.E., Katus H.A., Haas J. (2019). Identification and Regulation of the Long Non-Coding RNA Heat2 in Heart Failure. J. Mol. Cell. Cardiol..

[93] Gao L., Liu Y., Guo S., Yao R., Wu L., Xiao L., Wang Z., Liu Y., Zhang Y. (2017). Circulating Long Noncoding RNA HOTAIR Is an Essential Mediator of Acute Myocardial Infarction. Cell. Physiol. Biochem..

[94] Jha S., Thasma Loganathbabu V.K., Kumaran K., Krishnasamy G., Aruljothi K.N. (2024). Long Non-Coding RNAs (LncRNAs) in Heart Failure: A Comprehensive Review. Noncoding RNA.

[95] Jiao Y., Meng F., Ma G., Lei H., Liu J. (2023). An Increase in a Long Noncoding RNA ANRIL in Peripheral Plasma Is an Indicator of Stable Angina. Clinics.

[96] Greco S., Zaccagnini G., Perfetti A., Fuschi P., Valaperta R., Voellenkle C., Castelvecchio S., Gaetano C., Finato N., Beltrami A.P. (2016). Long Noncoding RNA Dysregulation in Ischemic Heart Failure. J. Transl. Med..

[97] Liu Z., Gao L., Kan C., Chen X., Shi K., Wang W. (2024). DNMT1 Methylation of LncRNA-ANRIL Causes Myocardial Fibrosis Pyroptosis by Interfering with the NLRP3/Caspase-1 Pathway. Cell. Mol. Biol..

[98] Chen L., Qu H., Guo M., Zhang Y., Cui Y., Yang Q., Bai R., Shi D. (2020). ANRIL and Atherosclerosis. J. Clin. Pharm. Ther..

[99] Bampatsias D., Mavroeidis I., Tual-Chalot S., Vlachogiannis N.I., Bonini F., Sachse M., Mavraganis G., Mareti A., Kritsioti C., Laina A. (2022). Beta-Secretase-1 Antisense RNA Is Associated with Vascular Ageing and Atherosclerotic Cardiovascular Disease. Thromb. Haemost..

[100] Greco S., Piella S.N., Made’ A., Tascini A.S., Garcia-Manteiga J.M., Castelvecchio S., Menicanti L., Martelli F. (2022). Basic Science-Cardiac Diseases LncRNA BACE1-AS: A Link between Heart Failure and Alzheimer’s Disease. Eur. Heart J..

[101] Greco S., Zaccagnini G., Fuschi P., Voellenkle C., Carrara M., Sadeghi I., Bearzi C., Maimone B., Castelvecchio S., Stellos K. (2017). Increased BACE1-AS Long Noncoding RNA and β-Amyloid Levels in Heart Failure. Cardiovasc. Res..

[102] Kontaraki J.E., Marketou M.E., Kochiadakis G.E., Patrianakos A., Maragkoudakis S., Plevritaki A., Papadaki S., Alevizaki A., Theodosaki O., Parthenakis F.I. (2021). Long Noncoding RNAs in Peripheral Blood Mononuclear Cells of Hypertensive Patients with Heart Failure with Preserved Ejection Fraction in Relation to Their Functional Capacity. Hell. J. Cardiol..

[103] Ounzain S., Micheletti R., Arnan C., Plaisance I., Cecchi D., Schroen B., Reverter F., Alexanian M., Gonzales C., Ng S.Y. (2015). CARMEN, a Human Super Enhancer-Associated Long Noncoding RNA Controlling Cardiac Specification, Differentiation and Homeostasis. J. Mol. Cell. Cardiol..

[104] Xu Y.l., Liu Y., Cai R.p., He S.r., Dai R.x., Yang X.h., Kong B.h., Qin Z.b., Su Q. (2020). Long Non-Coding RNA CASC7 Is Associated with the Pathogenesis of Heart Failure via Modulating the Expression of MiR-30c. J. Cell. Mol. Med..

[105] Liao B., Gao F., Lin F., Yang S., Xu Z., Dong S. (2022). LncRNA CASC7 Inhibits Myocardial Apoptosis in Myocardial Ischemia-Reperfusion Rats by Regulating MiR-21 Expression. Panminerva Med..

[106] Wang X., Wang L., Ma Z., Liang W., Li J., Li Y., Gui Y., Ai S. (2020). Early Expressed Circulating Long Noncoding RNA CHAST Is Associated with Cardiac Contractile Function in Patients with Acute Myocardial Infarction. Int. J. Cardiol..

[107] Viereck J., Kumarswamy R., Foinquinos A., Xiao K., Avramopoulos P., Kunz M., Dittrich M., Maetzig T., Zimmer K., Remke J. (2016). Long Noncoding RNA Chast Promotes Cardiac Remodeling. Sci. Transl. Med..

[108] Gong L., Zhu L., Yang T. (2020). Fendrr Involves in the Pathogenesis of Cardiac Fibrosis via Regulating MiR-106b/SMAD3 Axis. Biochem. Biophys. Res. Commun..

[109] Chen C., Lin X., Tang Y., Sun H., Yin L., Luo Z., Wang S., Liang P., Jiang B. (2023). LncRNA Fendrr: Involvement in the Protective Role of Nucleolin against H_2_O_2-_Induced Injury in Cardiomyocytes. Redox Rep..

[110] Wang X.M., Li X.M., Song N., Zhai H., Gao X.M., Yang Y.N. (2019). Long Non-Coding RNAs H19, MALAT1 and MIAT as Potential Novel Biomarkers for Diagnosis of Acute Myocardial Infarction. Biomed. Pharmacother..

[111] Omura J., Habbout K., Shimauchi T., Wu W.H., Breuils-Bonnet S., Tremblay E., Martineau S., Nadeau V., Gagnon K., Mazoyer F. (2020). Identification of Long Noncoding RNA H19 as a New Biomarker and Therapeutic Target in Right Ventricular Failure in Pulmonary Arterial Hypertension. Circulation.

[112] Tao H., Cao W., Yang J.J., Shi K.H., Zhou X., Liu L.P., Li J. (2016). Long Noncoding RNA H19 Controls DUSP5/ERK1/2 Axis in Cardiac Fibroblast Proliferation and Fibrosis. Cardiovasc. Pathol..

[113] Liu L., An X., Li Z., Song Y., Li L., Zuo S., Liu N., Yang G., Wang H., Cheng X. (2016). The H19 Long Noncoding RNA Is a Novel Negative Regulator of Cardiomyocyte Hypertrophy. Cardiovasc. Res..

[114] Tan W., Wang K., Yang X., Wang N., Jiang T.B. (2022). LncRNA HOTAIR Promotes Myocardial Fibrosis in Atrial Fibrillation through Binding with PTBP1 to Increase the Stability of Wnt5a. Int. J. Cardiol..

[115] Gao L., Wang X., Guo S., Xiao L., Liang C., Wang Z., Li Y., Liu Y., Yao R., Liu Y. (2019). LncRNA HOTAIR Functions as a Competing Endogenous RNA to Upregulate SIRT1 by Sponging MiR-34a in Diabetic Cardiomyopathy. J. Cell. Physiol..

[116] He L., Lu F., Zhang F., Fan S., Xu J. (2023). Mechanism of LncRNA HOTAIR in Attenuating Cardiomyocyte Pyroptosis in Mice with Heart Failure via the MiR-17-5p/RORA Axis. Exp. Cell Res..

[117] Yang F., Qin Y., Lv J., Wang Y., Che H., Chen X., Jiang Y., Li A., Sun X., Yue E. (2018). Silencing Long Non-Coding RNA Kcnq1ot1 Alleviates Pyroptosis and Fibrosis in Diabetic Cardiomyopathy. Cell Death Dis..

[118] Zhu L., Feng Q., Fan J., Huang J., Zhu Y., Wu Y., Hou A., Huo Y. (2023). Clinical Value of Long Non-Coding RNA KCNQ1OT1 in Estimating the Stenosis, Lipid Level, Inflammation Status, and Prognostication in Coronary Heart Disease Patients. J. Clin. Lab. Anal..

[119] Lai L., Xu Y., Kang L., Yang J., Zhu G. (2020). LncRNA KCNQ1OT1 Contributes to Cardiomyocyte Apoptosis by Targeting FUS in Heart Failure. Exp. Mol. Pathol..

[120] Kumarswamy R., Bauters C., Volkmann I., Maury F., Fetisch J., Holzmann A., Lemesle G., De Groote P., Pinet F., Thum T. (2014). Circulating Long Noncoding RNA, LIPCAR, Predicts Survival in Patients with Heart Failure. Circ. Res..

[121] Meessen J.M.T.A., Bär C., di Dona F.M., Staszewsky L.I., Di Giulio P., Di Tano G., Costa A., Leonardy J., Novelli D., Nicolis E.B. (2021). LIPCAR Is Increased in Chronic Symptomatic HF Patients. A Sub-Study of the GISSI-HF Trial. Clin. Chem..

[122] Yan L., Zhang Y., Wang M., Wang L., Zhang W., Ge Z.R. (2021). Circulating LIPCAR Is a Potential Biomarker of Heart Failure in Patients Post-Acute Myocardial Infarction. Exp. Biol. Med..

[123] Turkieh A., Beseme O., Saura O., Charrier H., Michel J.B., Amouyel P., Thum T., Bauters C., Pinet F. (2024). LIPCAR Levels in Plasma-Derived Extracellular Vesicles Is Associated with Left Ventricle Remodeling Post-Myocardial Infarction. J. Transl. Med..

[124] Wang X., Li D., Chen H., Wei X., Xu X. (2019). Expression of Long Noncoding RNA LIPCAR Promotes Cell Proliferation, Cell Migration, and Change in Phenotype of Vascular Smooth Muscle Cells. Med. Sci. Monit..

[125] Li R., Jin J., Liu E., Zhang J. (2022). A Novel Circulating Biomarker Lnc-MALAT1 for Acute Myocardial Infarction: Its Relationship with Disease Risk, Features, Cytokines, and Major Adverse Cardiovascular Events. J. Clin. Lab. Anal..

[126] Huang S., Zhang L., Song J., Wang Z., Huang X., Guo Z., Chen F., Zhao X. (2019). Long Noncoding RNA MALAT1 Mediates Cardiac Fibrosis in Experimental Postinfarct Myocardium Mice Model. J. Cell. Physiol..

[127] Liu J., Xu L., Zhan X. (2020). Lncrna Malat1 Regulates Diabetic Cardiac Fibroblasts through the Hippo–Yap Signaling Pathway. Biochem. Cell Biol..

[128] Gong X., Zhu Y., Chang H., Li Y., Ma F. (2019). Long Noncoding RNA MALAT1 Promotes Cardiomyocyte Apoptosis after Myocardial Infarction via Targeting MIR-144-3p. Biosci. Rep..

[129] Zhang L., Wu Y.J., Zhang S.L. (2019). Circulating LncRNA MHRT Predicts Survival of Patients with Chronic Heart Failure. J. Geriatr. Cardiol..

[130] Xuan L., Sun L., Zhang Y., Huang Y., Hou Y., Li Q., Guo Y., Feng B., Cui L., Wang X. (2017). Circulating Long Non-Coding RNAs NRON and MHRT as Novel Predictive Biomarkers of Heart Failure. J. Cell. Mol. Med..

[131] Zhang J., Gao C., Meng M., Tang H. (2016). Long Noncoding RNA MHRT Protects Cardiomyocytes against H_2_O_2_-Induced Apoptosis. Biomol. Ther..

[132] Han P., Li W., Lin C.H., Yang J., Shang C., Nurnberg S.T., Jin K.K., Xu W., Lin C.Y., Lin C.J. (2014). A Long Noncoding RNA Protects the Heart from Pathological Hypertrophy. Nature.

[133] Lang M., Ou D., Liu Z., Li Y., Zhang X., Zhang F. (2021). LncRNA MHRT Promotes Cardiac Fibrosis via MiR-3185 Pathway Following Myocardial Infarction. Int. Heart J..

[134] Vausort M., Wagner D.R., Devaux Y. (2014). Long Noncoding RNAs in Patients with Acute Myocardial Infarction. Circ. Res..

[135] Zhao X., Ren Y., Ren H., Wu Y., Liu X., Chen H., Ying C. (2021). The Mechanism of Myocardial Fibrosis Is Ameliorated by Myocardial Infarction-Associated Transcript through the PI3K/Akt Signaling Pathway to Relieve Heart Failure. J. Int. Med. Res..

[136] Qi Y., Wu H., Mai C., Lin H., Shen J., Zhang X., Gao Y., Mao Y., Xie X. (2020). LncRNA-MIAT-Mediated MiR-214-3p Silencing Is Responsible for IL-17 Production and Cardiac Fibrosis in Diabetic Cardiomyopathy. Front. Cell Dev. Biol..

[137] Yan L., Zhang Y., Zhang W., Deng S.Q., Ge Z.R. (2020). LncRNA-NRF Is a Potential Biomarker of Heart Failure After Acute Myocardial Infarction. J. Cardiovasc. Transl. Res..

[138] Wang K., Liu F., Liu C.Y., An T., Zhang J., Zhou L.Y., Wang M., Dong Y.H., Li N., Gao J.N. (2016). The Long Noncoding RNA NRF Regulates Programmed Necrosis and Myocardial Injury during Ischemia and Reperfusion by Targeting MiR-873. Cell Death Differ..

[139] Gharbi N., Mahmoudinasab H., Hooshmandi E., Rahimi M., Bayat M., Karimi N., Hojati S.S., Zayani Z., Tabrizi R., Borhani-Haghighi A. (2024). Altered Expression of Long Non-Coding RNAs NRON and SNHG11 in Patients with Ischemic Stroke. Egypt. J. Med. Hum. Genet..

[140] Sun F., Guo Z., Zhang C., Che H., Gong W., Shen Z., Shi Y., Ge S. (2019). LncRNA NRON Alleviates Atrial Fibrosis through Suppression of M1 Macrophages Activated by Atrial Myocytes. Biosci. Rep..

[141] Wang Y., Xu P., Zhang C., Feng J., Gong W., Ge S., Guo Z. (2019). LncRNA NRON Alleviates Atrial Fibrosis via Promoting NFATc3 Phosphorylation. Mol. Cell. Biochem..

[142] Hoepfner J., Leonardy J., Lu D., Schmidt K., Hunkler H.J., Biß S., Foinquinos A., Xiao K., Regalla K., Ramanujam D. (2022). The Long Non-Coding RNA NRON Promotes the Development of Cardiac Hypertrophy in the Murine Heart. Mol. Ther..

[143] Sun B., Meng M., Wei J., Wang S. (2020). Long Noncoding RNA PVT1 Contributes to Vascular Endothelial Cell Proliferation via Inhibition of MiR-190a-5p in Diagnostic Biomarker Evaluation of Chronic Heart Failure. Exp. Ther. Med..

[144] Cao F., Li Z., Ding W.M., Yan L., Zhao Q.Y. (2019). LncRNA PVT1 Regulates Atrial Fibrosis via MiR-128-3p-SP1-TGF-Β1-Smad Axis in Atrial Fibrillation. Mol. Med..

[145] Tian C., Hu S., Yu J., Li W., Li P., Huang H. (2022). CREB1 Transcription-Activated LncRNA PVT1 Promotes Cardiac Fibrosis via MiR-145/HCN1 Axis. Int. J. Cardiol..

[146] Hu Y., Luo M., Xue Y., Lv D., Huang L., Li X., Shen J. (2024). LncRNA Pvt1 Aggravates Cardiomyocyte Apoptosis via the MicroRNA-216/Ccnd3 Axis. Heliyon.

[147] Ramos K.S., Li J., Wijdeveld L.F.J., van Schie M.S., Taverne Y.J.H.J., Boon R.A., de Groot N.M.S., Brundel B.J.J.M. (2023). Long Noncoding RNA UCA1 Correlates with Electropathology in Patients with Atrial Fibrillation. JACC Clin. Electrophysiol..

[148] Trembinski D.J., Bink D.I., Theodorou K., Sommer J., Fischer A., van Bergen A., Kuo C.C., Costa I.G., Schürmann C., Leisegang M.S. (2020). Aging-Regulated Anti-Apoptotic Long Non-Coding RNA Sarrah Augments Recovery from Acute Myocardial Infarction. Nat. Commun..

[149] Yu Y., Ge X., Cao L., Li F. (2024). Diagnostic and Prognostic Value of Plasma LncRNA SRA1 in Chronic Heart Failure. Rev. Cardiovasc. Med..

[150] Zhang S., Gao S., Wang Y., Jin P., Lu F. (2019). LncRNA SRA1 Promotes the Activation of Cardiac Myofibroblasts Through Negative Regulation of MiR-148b. DNA Cell Biol..

[151] Zhang C., Pan S., Aisha A., Abudoukelimu M., Tang L., Ling Y. (2018). Long Noncoding RNA SRA1 Attenuates Hypoxia-Induced Injury in H9c2 Cardiomyocytes through Regulating PPARγ/NF-ΚB Signaling Pathway. Int. J. Clin. Exp. Pathol..

[152] Zhang S., Jin R., Li B. (2021). Serum NT-proBNP and TUG1 as Novel Biomarkers for Elderly Hypertensive Patients with Heart Failure with Preserved Ejection Fraction. Exp. Ther. Med..

[153] Zhu Q., Li S., Ji K., Zhou H., Luo C., Sui Y. (2021). Differentially Expressed TUG1 and MiR-145-5p Indicate Different Severity of Chronic Heart Failure and Predict 2-year Survival Prognosis. Exp. Ther. Med..

[154] Fu D., Gao T., Liu M., Li C., Li H., Jiang H., Li X. (2021). LncRNA TUG1 Aggravates Cardiomyocyte Apoptosis and Myocardial Ischemia/Reperfusion Injury. Histol. Histopathol..

[155] Sun Y., Shan X., Guo J., Liu X., Ma D. (2023). CHI3L1 Promotes Myocardial Fibrosis via Regulating LncRNA TUG1/MiR-495-3p/ETS1 Axis. Apoptosis.

[156] Yang D., Yu J., Liu H.B., Yan X.Q., Hu J., Yu Y., Guo J., Yuan Y., Du Z.M. (2019). The Long Non-Coding RNA TUG1-MiR-9a-5p Axis Contributes to Ischemic Injuries by Promoting Cardiomyocyte Apoptosis via Targeting KLF5. Cell Death Dis..

[157] Su Q., Liu Y., Lv X.W., Dai R.X., Yang X.H., Kong B.H. (2020). LncRNA TUG1 Mediates Ischemic Myocardial Injury by Targeting MiR-132-3p/HDAC3 Axis. Am. J. Physiol. Heart Circ. Physiol..

[158] Cheng Q., Zhang M., Zhang M., Ning L., Chen D. (2020). Long Non-Coding RNA LOC285194 Regulates Vascular Smooth Muscle Cell Apoptosis in Atherosclerosis. Bioengineered.

[159] Yan Y., Zhang B., Liu N., Qi C., Xiao Y., Tian X., Li T., Liu B. (2016). Circulating Long Noncoding RNA UCA1 as a Novel Biomarker of Acute Myocardial Infarction. Biomed. Res. Int..

[160] Yang Z., Zhang H., Yin M., Cheng Z., Jiang P., Feng M., Liu Z., Liao B. (2022). TGF-Β1/Smad3 Upregulates UCA1 to Promote Liver Fibrosis through DKK1 and MiR18a. J. Mol. Med..

[161] Zhou G., Li C., Feng J., Zhang J., Fang Y. (2018). LncRNA UCA1 Is a Novel Regulator in Cardiomyocyte Hypertrophy through Targeting the MiR-184/HOXA9 Axis. Cardiorenal Med..

[162] Huang K., Huang D., Li Q., Zhong J., Zhou Y., Zhong Z., Tang S., Zhang W., Chen Z., Lu S. (2024). Upregulation of LncRNA UCA1 Promotes Cardiomyocyte Proliferation by Inhibiting the MiR-128/SUZ12/P27 Pathway. Heliyon.

[163] Carrara M., Fuschi P., Ivan C., Martelli F. (2018). Circular RNAs: Methodological Challenges and Perspectives in Cardiovascular Diseases. J. Cell. Mol. Med..

[164] Santer L., Bär C., Thum T. (2019). Circular RNAs: A Novel Class of Functional RNA Molecules with a Therapeutic Perspective. Mol. Ther..

[165] Madè A., Bibi A., Garcia-Manteiga J.M., Tascini A.S., Piella S.N., Tikhomirov R., Voellenkle C., Gaetano C., Leszek P., Castelvecchio S. (2023). CircRNA-MiRNA-MRNA Deregulated Network in Ischemic Heart Failure Patients. Cells.

[166] Salgado-Somoza A., Zhang L., Vausort M., Devaux Y. (2017). The Circular RNA MICRA for Risk Stratification after Myocardial Infarction. IJC Heart Vasc..

[167] Li H., Xu J.D., Fang X.H., Zhu J.N., Yang J., Pan R., Yuan S.J., Zeng N., Yang Z.Z., Yang H. (2021). Circular RNA CircRNA_000203 Aggravates Cardiac Hypertrophy via Suppressing MiR-26b-5p and MiR-140-3p Binding to Gata4. Cardiovasc. Res..

[168] Bibi A., Bartekova M., Gandhi S., Greco S., Madè A., Sarkar M., Stopa V., Tastsoglou S., de Gonzalo-Calvo D., Devaux Y. (2024). Circular RNA Regulatory Role in Pathological Cardiac Remodelling. Br. J. Pharmacol..

[169] Ren L., Jiang Q., Mo L., Tan L., Dong Q., Meng L., Yang N., Li G. (2022). Mechanisms of Circular RNA Degradation. Commun. Biol..

[170] Ge L., Sun Y., Shi Y., Liu G., Teng F., Geng Z., Chen X., Xu H., Xu J., Jia X. (2022). Plasma CircRNA Microarray Profiling Identifies Novel CircRNA Biomarkers for the Diagnosis of Ovarian Cancer. J. Ovarian Res..

[171] Sun Y., Jiang X., Lv Y., Liang X., Zhao B., Bian W., Zhang D., Jiang J., Zhang C. (2020). Circular RNA Expression Profiles in Plasma from Patients with Heart Failure Related to Platelet Activity. Biomolecules.

[172] Hughes J., Dawson R., Tea M., McAninch D., Piltz S., Jackson D., Stewart L., Ricos M.G., Dibbens L.M., Harvey N.L. (2017). Knockout of the Epilepsy Gene Depdc5 in Mice Causes Severe Embryonic Dysmorphology with Hyperactivity of MTORC1 Signalling. Sci. Rep..

[173] He S., Zhu H., Zhang J., Yang X., Zhao L. (2023). Genome-Wide Screening for CircRNAs in Epicardial Adipose Tissue of Heart Failure Patients with Preserved Ejection Fraction. Am. J. Transl. Res..

[174] Pan R.Y., Liu P., Zhou H.T., Sun W.X., Song J., Shu J., Cui G.J., Yang Z.J., Jia E.Z. (2017). Circular RNAs Promote TRPM3 Expression by Inhibiting Hsa-MiR- 130a-3p in Coronary Artery Disease Patients. Oncotarget.

[175] Chen Y., Zhou J., Wei Z., Cheng Y., Tian G., Quan Y., Kong Q., Wu W., Liu X. (2022). Identification of Circular RNAs in Cardiac Hypertrophy and Cardiac Fibrosis. Front. Pharmacol..

[176] Imanishi R., Nakau K., Shimada S., Oka H., Takeguchi R., Tanaka R., Sugiyama T., Nii M., Okamoto T., Nagaya K. (2023). A Novel HECW2 Variant in an Infant with Congenital Long QT Syndrome. Hum. Genome Var..

[177] Vilades D., Martínez-Camblor P., Ferrero-Gregori A., Bär C., Lu D., Xiao K., Vea À., Nasarre L., Sanchez Vega J., Leta R. (2020). Plasma Circular RNA Hsa_circ_0001445 and Coronary Artery Disease: Performance as a Biomarker. FASEB J..

[178] Dinh P.S., Peng J.H., Tran T.L., Wu D.F., Tran C.M.T., Dinh T.P.H., Pan S.L. (2023). Identification of Hsa_circ_0001445 of a Novel CircRNA-MiRNA-MRNA Regulatory Network as Potential Biomarker for Coronary Heart Disease. Front. Cardiovasc. Med..

[179] Zeng Y., Du W.W., Wu Y., Yang Z., Awan F.M., Li X., Yang W., Zhang C., Yang Q., Yee A. (2017). A Circular RNA Binds to and Activates AKT Phosphorylation and Nuclear Localization Reducing Apoptosis and Enhancing Cardiac Repair. Theranostics.

[180] Yang Y., Yang H., Yang C. (2023). Circ-AMOTL1 Enhances Cardiac Fibrosis through Binding with EIF4A3 and Stabilizing MARCKS Expression in Diabetic Cardiomyopathy. Cell Signal.

[181] Du W.W., Yang W., Chen Y., Wu Z.-K., Foster F.S., Yang Z., Li X., Yang B.B. (2016). Foxo3 Circular RNA Promotes Cardiac Senescence by Modulating Multiple Factors Associated with Stress and Senescence Responses. Eur. Heart J..

[182] Sun G., Shen J.F., Wei X.F., Qi G.X. (2021). Circular RNA Foxo3 Relieves Myocardial Ischemia/ Reperfusion Injury by Suppressing Autophagy via Inhibiting HMGB1 by Repressing KAT7 in Myocardial Infarction. J. Inflamm. Res..

[183] Liang B., Li M., Deng Q., Wang C., Rong J., He S., Xiang Y., Zheng F. (2020). CircRNA ZNF609 in Peripheral Blood Leukocytes Acts as a Protective Factor and a Potential Biomarker for Coronary Artery Disease. Ann. Transl. Med..

[184] Vausort M., Salgado-Somoza A., Zhang L., Leszek P., Scholz M., Teren A., Burkhardt R., Thiery J., Wagner D.R., Devaux Y. (2016). Myocardial Infarction-Associated Circular RNA Predicting Left Ventricular Dysfunction. J. Am. Coll. Cardiol..

[185] Sun W., Zhou S., Peng L., Liu Y., Cheng D., Wang Y., Ni C. (2023). CircZNF609 Regulates Pulmonary Fibrosis via MiR-145-5p/KLF4 Axis and Its Translation Function. Cell. Mol. Biol. Lett..

[186] Devaux Y., Zhang L. (2020). Novel Circular RNA Biomarkers for Heart Failure 2018. U.S. Patent.

[187] Wang C., Liu Y., Zhang W., Huang J., Jiang J., Wang R., Zeng D. (2023). Circ-Bptf Serves as a Mir-486-5p Sponge to Regulate Cemip and Promotes Hypoxic Pulmonary Arterial Smooth Muscle Cell Proliferation in Copd. Acta Biochim. Biophys. Sin..

[188] Chen H., Lv L., Liang R., Guo W., Liao Z., Chen Y., Zhu K., Huang R., Zhao H., Pu Q. (2022). MiR-486 Improves Fibrotic Activity in Myocardial Infarction by Targeting SRSF3/P21-Mediated Cardiac Myofibroblast Senescence. J. Cell. Mol. Med..

[189] Zhang W., Sui Y. (2020). CircBPTF Knockdown Ameliorates High Glucose-Induced Inflammatory Injuries and Oxidative Stress by Targeting the MiR-384/LIN28B Axis in Human Umbilical Vein Endothelial Cells. Mol. Cell. Biochem..

[190] Liu R., Hu L., Zhou Y., Cao Y. (2024). Serum CircPRDM5 as a Novel Diagnostic Biomarker for Acute Myocardial Infarction. Gene.

[191] Huang P., Hu Y., Duan Y. (2022). TGF-Β2-Induced Circ-PRDM5 Regulates Migration, Invasion, and EMT through the MiR-92b-3p/COL1A2 Pathway in Human Lens Epithelial Cells. J. Mol. Histol..

[192] Garikipati V.N.S., Verma S.K., Cheng Z., Liang D., Truongcao M.M., Cimini M., Yue Y., Huang G., Wang C., Benedict C. (2019). Circular RNA CircFndc3b Modulates Cardiac Repair after Myocardial Infarction via FUS/VEGF-A Axis. Nat. Commun..

[193] Sun K., Yao H., Zhang P., Sun Y., Ma J., Xia Q. (2023). Emerging Landscape of CircFNDC3B and Its Role in Human Malignancies. Front. Oncol..

[194] Bazan H.A., Hatfield S.A., Brug A., Brooks A.J., Lightell D.J., Woods T.C. (2017). Carotid Plaque Rupture Is Accompanied by an Increase in the Ratio of Serum CircR-284 to MiR-221 Levels. Circ. Cardiovasc. Genet..

[195] Liu X., Zheng M., Han R., Yu Z., Yuan W., Xie B., Zhang Y., Zhong J., Wang L., Wang L. (2023). Circulating Exosomal CircRNAs as Diagnostic Biomarkers for Chronic Coronary Syndrome. Metabolites.

[196] Chen M., Cai Y., Guo J., Gong Y., Xu X., Lin Y., Hu Y., Wen Y., Yang L., Li H. (2023). Circ_0000284: A Risk Factor and Potential Biomarker for Prehypertension and Hypertension. Hypertens. Res..

[197] Wang W., Zhang S., Xu L., Feng Y., Wu X., Zhang M., Yu Z., Zhou X. (2021). Involvement of CircHIPK3 in the Pathogenesis of Diabetic Cardiomyopathy in Mice. Diabetologia.

[198] Ni H., Li W., Zhuge Y., Xu S., Wang Y., Chen Y., Shen G., Wang F. (2019). Inhibition of CircHIPK3 Prevents Angiotensin II-Induced Cardiac Fibrosis by Sponging MiR-29b-3p. Int. J. Cardiol..

[199] Zheng M.L., Du X.P., Zhao L., Yang X.C., Hao X.Y., Chen X. (2020). Expression Profile of Circular RNAs in Epicardial Adipose Tissue in Heart Failure. Chin. Med. J..

[200] Zhu Y., Pan W., Yang T., Meng X., Jiang Z., Tao L., Wang L. (2019). Upregulation of Circular RNA CircNFIB Attenuates Cardiac Fibrosis by Sponging MiR-433. Front. Genet..

[201] Liu J., Zhang R., Wang D., Lin Y., Bai C., Nie N., Gao S., Zhang Q., Chang H., Ren C. (2022). Elucidating the Role of CircNFIB in Myocardial Fibrosis Alleviation by Endogenous Sulfur Dioxide. BMC Cardiovasc. Disord..

[202] Chen C., Shen H., Huang Q., Li Q. (2020). The Circular RNA CDR1as Regulates the Proliferation and Apoptosis of Human Cardiomyocytes through the MiR-135a/HMOX1 and MiR-135b/HMOX1 Axes. Genet. Test. Mol. Biomark..

[203] Gonzalez C., Cimini M., Benedict C., Mallaredy V., Trungcao M., Rajan S., Garikipati V.N., Kishore R. (2023). Abstract P3090: The Role Of Circular Cdr1as In Macrophage Cardiac Inflammation And Fibrosis. Circ. Res..

[204] Mester-Tonczar J., Winkler J., Einzinger P., Hasimbegovic E., Kastner N., Lukovic D., Zlabinger K., Spannbauer A., Traxler D., Batkai S. (2020). Association between Circular Rna Cdr1as and Post-Infarction Cardiac Function in Pig Ischemic Heart Failure: Influence of the Anti-Fibrotic Natural Compounds Bufalin and Lycorine. Biomolecules.

[205] Han J., Zhang L., Hu L., Yu H., Xu F., Yang B., Zhang R., Zhang Y., An Y. (2020). Circular RNA-Expression Profiling Reveals a Potential Role of Hsa_circ_0097435 in Heart Failure via Sponging Multiple MicroRNAs. Front. Genet..

[206] Sun L.Y., Zhao J.C., Ge X.M., Zhang H., Wang C.M., Bie Z.D. (2020). Circ_LAS1L Regulates Cardiac Fibroblast Activation, Growth, and Migration through MiR-125b/SFRP5 Pathway. Cell Biochem. Funct..

[207] Sonnenschein K., Wilczek A.L., de Gonzalo-Calvo D., Pfanne A., Derda A.A., Zwadlo C., Bavendiek U., Bauersachs J., Fiedler J., Thum T. (2019). Serum Circular RNAs Act as Blood-Based Biomarkers for Hypertrophic Obstructive Cardiomyopathy. Sci. Rep..

[208] Wang S., Li L., Deng W., Jiang M. (2021). CircRNA MFACR Is Upregulated in Myocardial Infarction and Downregulates MiR-125b to Promote Cardiomyocyte Apoptosis Induced by Hypoxia. J. Cardiovasc. Pharmacol..

[209] Wang K., Gan T.Y., Li N., Liu C.Y., Zhou L.Y., Gao J.N., Chen C., Yan K.W., Ponnusamy M., Zhang Y.H. (2017). Circular RNA Mediates Cardiomyocyte Death via MiRNA-Dependent Upregulation of MTP18 Expression. Cell Death Differ..

[210] Zhao Z., Li X., Gao C., Jian D., Hao P., Rao L., Li M. (2017). Peripheral Blood Circular RNA Hsa_circ_0124644 Can Be Used as a Diagnostic Biomarker of Coronary Artery Disease. Sci. Rep..

[211] Chen T.p., Zhang N.j., Wang H.j., Hu S.g., Geng X. (2021). Knockdown of CircROBO2 Attenuates Acute Myocardial Infarction through Regulating the MiR-1184/TRADD Axis. Mol. Med..

[212] Guo J., Chen L.W., Huang Z.Q., Guo J.S., Li H., Shan Y., Chen Z.R., Yan Y.M., Zhu J.N., Guo H.M. (2022). Suppression of the Inhibitory Effect of Circ_0036176-Translated Myo9a-208 on Cardiac Fibroblast Proliferation by MiR-218-5p. J. Cardiovasc. Transl. Res..

[213] D’alessandra Y., Chiesa M., Carena M.C., Beltrami A.P., Rizzo P., Buzzetti M., Ricci V., Ferrari R., Fucili A., Livi U. (2020). Differential Role of Circulating Micrornas to Track Progression and Pre-Symptomatic Stage of Chronic Heart Failure: A Pilot Study. Biomedicines.

[214] Seronde M.F., Vausort M., Gayat E., Goretti E., Ng L.L., Squire I.B., Vodovar N., Sadoune M., Samuel J.L., Thum T. (2015). Circulating MicroRNAs and Outcome in Patients with Acute Heart Failure. PLoS ONE.

[215] Xu M., Liu D., Gao X., Wang Z., Zhang L., Fan H. (2023). MiR-423-5p Inhibition Exerts Protective Effects on Angiotensin II-Induced Cardiomyocyte Hypertrophy. Tohoku J. Exp. Med..

[216] Pradhan K., Niehues P., Neupane B., Maleck C., Sharif-Yakan A., Emrani M., Zink M.D., Napp A., Marx N., Gramlich M. (2023). MicroRNA-21 Mediated Cross-Talk between Cardiomyocytes and Fibroblasts in Patients with Atrial Fibrillation. Front. Cardiovasc. Med..

[217] Torres-Paz Y.E., Gamboa R., Fuentevilla-Álvarez G., Soto M.E., González-Moyotl N., Martínez-Alvarado R., Torres-Tamayo M., Ramírez-Marroquín E.S., Vásquez-Jiménez X., Sainz-Escarrega V. (2023). Overexpression of MicroRNA-21-5p and MicroRNA-221-5p in Monocytes Increases the Risk of Developing Coronary Artery Disease. Int. J. Mol. Sci..

[218] Tikhomirov R., Reilly-O’donnell B., Lucarelli C., Greco S., Zacagnini G., Maryam A., Menicanti L., Leszek P., Faggian G., Srivastava P. (2021). Identification of MiRNA-497 and MiRNA-27b-5p as Potential Diagnostic Markers of Cardiac Fibrosis. Eur. Heart J..

[219] Bahls M., Könemann S., Markus M.R.P., Wenzel K., Friedrich N., Nauck M., Völzke H., Steveling A., Janowitz D., Grabe H.J. (2019). Brain-Derived Neurotrophic Factor Is Related with Adverse Cardiac Remodeling and High NTproBNP. Sci. Rep..

[220] Jiang Y., Mo H., Luo J., Zhao S., Liang S., Zhang M., Yuan J. (2018). HOTAIR Is a Potential Novel Biomarker in Patients with Congenital Heart Diseases. Biomed. Res. Int..

[221] Taghvimi S., Soltani Fard E., Khatami S.H., Zafaranchi Z.M.S., Taheri-Anganeh M., Movahedpour A., Ghasemi H. (2024). LncRNA HOTAIR and Cardiovascular Diseases. Funct. Integr. Genom..

[222] Lai Y., He S., Ma L., Lin H., Ren B., Ma J., Zhu X., Zhuang S. (2017). HOTAIR Functions as a Competing Endogenous RNA to Regulate PTEN Expression by Inhibiting MiR-19 in Cardiac Hypertrophy. Mol. Cell Biochem..

[223] Pan S.C., Cui H.H., Qiu C.G. (2018). HOTAIR Promotes Myocardial Fibrosis through Regulating URI1 Expression via Wnt Pathway. Eur. Rev. Med. Pharmacol. Sci..

[224] Baral H., Uchiyama A., Yokoyama Y., Sekiguchi A., Yamazaki S., Amalia S.N., Inoue Y., Ogino S., Torii R., Hosoi M. (2021). Antifibrotic Effects and Mechanisms of Mesenchymal Stem Cell-Derived Exosomes in a Systemic Sclerosis Mouse Model: Possible Contribution of MiR-196b-5p. J. Dermatol. Sci..

[225] Bei Y., Lu D., Bär C., Chatterjee S., Costa A., Riedel I., Mooren F.C., Zhu Y., Huang Z., Wei M. (2022). MiR-486 Attenuates Cardiac Ischemia/Reperfusion Injury and Mediates the Beneficial Effect of Exercise for Myocardial Protection. Mol. Ther..

[226] Douvris A., Viñas J., Burns K.D. (2022). MiRNA-486-5p: Signaling Targets and Role in Non-Malignant Disease. Cell. Mol. Life Sci..

[227] Jiang C., Zeng X., Shan R., Wen W., Li J., Tan J., Li L., Wan R. (2020). The Emerging Picture of the Roles of CircRNA-CDR1as in Cancer. Front. Cell Dev. Biol..

[228] Hirt M.N., Werner T., Indenbirken D., Alawi M., Demin P., Kunze A.C., Stenzig J., Starbatty J., Hansen A., Fiedler J. (2015). Deciphering the MicroRNA Signature of Pathological Cardiac Hypertrophy by Engineered Heart Tissue- and Sequencing-Technology. J. Mol. Cell. Cardiol..

[229] Borlaug B.A., Sharma K., Shah S.J., Ho J.E. (2023). Heart Failure with Preserved Ejection Fraction: JACC Scientific Statement. J. Am. Coll. Cardiol..

[230] Omote K., Verbrugge F.H., Borlaug B.A. (2022). Heart Failure with Preserved Ejection Fraction: Mechanisms and Treatment Strategies. Annu. Rev. Med..

[231] Jie R., Zhu P., Zhong J., Zhang Y., Wu H. (2020). LncRNA KCNQ1OT1 Affects Cell Proliferation, Apoptosis and Fibrosis through Regulating MiR-18b-5p/SORBS2 Axis and NF-KB Pathway in Diabetic Nephropathy. Diabetol. Metab. Syndr..

[232] Lazar S., Rayner B., Lopez Campos G., McGrath K., McClements L. (2020). Mechanisms of Heart Failure with Preserved Ejection Fraction in the Presence of Diabetes Mellitus. Transl. Metab. Syndr. Res..

[233] Mgbemena O., Zhang Y., Velarde G. (2021). Role of Diabetes Mellitus in Heart Failure with Preserved Ejection Fraction: A Review Article. Cureus.

[234] Scărlătescu A.I., Barbălată T., Sima A.V., Stancu C., Niculescu L.Ș., Micheu M.M. (2022). MiR-146a-5p, MiR-223-3p and MiR-142-3p as Potential Predictors of Major Adverse Cardiac Events in Young Patients with Acute ST Elevation Myocardial Infarction—Added Value over Left Ventricular Myocardial Work Indices. Diagnostics.

[235] Zhang G., Dou L., Chen Y. (2020). Association of Long-Chain Non-Coding RNA MHRT Gene Single Nucleotide Polymorphism with Risk and Prognosis of Chronic Heart Failure. Medicine.

[236] Murphy S.P., Ibrahim N.E., Januzzi J.L. (2020). Heart Failure with Reduced Ejection Fraction: A Review. JAMA-J. Am. Med. Assoc..

[237] Chen Y.T., Wong L.L., Liew O.W., Richards A.M. (2019). Heart Failure with Reduced Ejection Fraction (HFrEF) and Preserved Ejection Fraction (HFpEF): The Diagnostic Value of Circulating MicroRNAs. Cells.

[238] Takvorian K.S., Wang D., Larson M.G., Courchesne P., Levy D., Ho J.E. (2021). The Association of Protein Biomarkers with Incident HFpEF vs HFrEF. J. Am. Coll. Cardiol..

[239] Lala A., Desai A.S. (2014). The Role of Coronary Artery Disease in Heart Failure. Heart Fail. Clin..

[240] Lin D.S., Zhang C.Y., Li L., Ye G.H., Jiang L.P., Jin Q. (2021). Circ_ROBO2/MiR-149 Axis Promotes the Proliferation and Migration of Human Aortic Smooth Muscle Cells by Activating NF-ΚB Signaling. Cytogenet. Genome Res..

[241] Li X., Zhang Y., Ding Z., Chen Y., Wang W. (2024). LncRNA H19: A Novel Biomarker in Cardiovascular Disease. Acta Cardiol. Sin..

[242] Zhang Z., Gao W., Long Q.Q., Zhang J., Li Y.F., Liu D.C., Yan J.J., Yang Z.J., Wang L.S. (2017). Increased Plasma Levels of LncRNA H19 and LIPCAR Are Associated with Increased Risk of Coronary Artery Disease in a Chinese Population. Sci. Rep..

[243] Busscher D., Boon R.A., Juni R.P. (2022). The Multifaceted Actions of the LncRNA H19 in Cardiovascular Biology and Diseases. Clin. Sci..

[244] Zhang L., Wang Y., Yu F., Li X., Gao H., Li P. (2021). CircHIPK3 Plays Vital Roles in Cardiovascular Disease. Front. Cardiovasc. Med..

[245] Deng Y., Wang J., Xie G., Zeng X., Li H. (2019). Circ-Hipk3 Strengthens the Effects of Adrenaline in Heart Failure by Mir-17-3p-Adcy6 Axis. Int. J. Biol. Sci..

[246] Liu X., Cheng Y., Zhang S., Lin Y., Yang J., Zhang C. (2009). A Necessary Role of MiR-221 and MiR-222 in Vascular Smooth Muscle Cell Proliferation and Neointimal Hyperplasia. Circ. Res..

[247] Jenča D., Melenovský V., Stehlik J., Staněk V., Kettner J., Kautzner J., Adámková V., Wohlfahrt P. (2021). Heart Failure after Myocardial Infarction: Incidence and Predictors. ESC Heart Fail..

[248] Arun G., Aggarwal D., Spector D.L. (2020). MALAT1 Long Non-Coding RNA: Functional Implications. Noncoding RNA.

[249] Zhang T., Luo J.Y., Liu F., Zhang X.H., Luo F., Yang Y.N., Li X.M. (2022). Long Noncoding RNA MALAT1 Polymorphism Predicts MACCEs in Patients with Myocardial Infarction. BMC Cardiovasc. Disord..

[250] Li Y., Liu F., Cai Y., Yang Y., Wang Y. (2021). Lncrna Malat1: A Potential Fibrosis Biomarker and Therapeutic Target. Crystals.

[251] De Gonzalo-Calvo D., Kenneweg F., Bang C., Toro R., Van Der Meer R.W., Rijzewijk L.J., Smit J.W., Lamb H.J., Llorente-Cortes V., Thum T. (2016). Circulating Long-Non Coding RNAs as Biomarkers of Left Ventricular Diastolic Function and Remodelling in Patients with Well-Controlled Type 2 Diabetes. Sci. Rep..

[252] McEwan D.G., Popovic D., Gubas A., Terawaki S., Suzuki H., Stadel D., Coxon F.P., MirandadeStegmann D., Bhogaraju S., Maddi K. (2015). PLEKHM1 Regulates Autophagosome-Lysosome Fusion through HOPS Complex and LC3/GABARAP Proteins. Mol. Cell.

[253] Kotecha D., Piccini J.P. (2015). Atrial Fibrillation in Heart Failure: What Should We Do?. Eur. Heart J..

[254] Bitarafan S., Yari M., Broumand M.A., Ghaderian S.M.H., Rahimi M., Mirfakhraie R., Azizi F., Omrani M.D. (2019). Association of Increased Levels of LncRNA H19 in PBMCs with Risk of Coronary Artery Disease. Cell J..

[255] Zhou B., Yu J.W. (2017). A Novel Identified Circular RNA, CircRNA_010567, Promotes Myocardial Fibrosis via Suppressing MiR-141 by Targeting TGF-Β1. Biochem. Biophys. Res. Commun..

[256] Bai M., Pan C.L., Jiang G.X., Zhang Y.M. (2020). CircRNA 010567 Improves Myocardial Infarction Rats through Inhibiting TGF-Β1. Eur. Rev. Med. Pharmacol. Sci..

[257] Zhao Q., Li W., Pan W., Wang Z. (2021). CircRNA 010567 Plays a Significant Role in Myocardial Infarction via the Regulation of the MiRNA-141/DAPK1 Axis. J. Thorac. Dis..

[258] Tang C.-M., Zhang M., Huang L., Hu Z., Zhu J.-N., Xiao Z., Zhang Z., Lin Q., Zheng X.-L., Yang M. (2017). CircRNA_000203 Enhances the Expression of Fibrosis-Associated Genes by Derepressing Targets of MiR-26b-5p, Col1a2 and CTGF, in Cardiac Fibroblasts. Sci. Rep..

[259] Zhang L., Lou Q., Zhang W., Yang W., Li L., Zhao H., Kong Y., Li W. (2023). CircCAMTA1 Facilitates Atrial Fibrosis by Regulating the MiR-214-3p/TGFBR1 Axis in Atrial Fibrillation. J. Mol. Histol..

[260] Li F., Long T.Y., Bi S.S., Sheikh S.A., Zhang C.L. (2020). CircPAN3 Exerts a Profibrotic Role via Sponging MiR-221 through FoxO3/ATG7-Activated Autophagy in a Rat Model of Myocardial Infarction. Life Sci..

[261] Pang P., Si W., Wu H., Wang C., Liu K., Jia Y., Zhang Z., Zhang F., Kong X., Yang Y. (2023). The Circular RNA CircHelz Enhances Cardiac Fibrosis by Facilitating the Nuclear Translocation of YAP1. Transl. Res..

[262] Ji D.N., Jin S.D., Jiang Y., Xu F.Y., Fan S.W., Zhao Y.L., Liu X.Q., Sun H., Cheng W.Z., Zhang X.Y. (2024). CircNSD1 Promotes Cardiac Fibrosis through Targeting the MiR-429-3p/SULF1/Wnt/β-Catenin Signaling Pathway. Acta Pharmacol. Sin..

[263] Wu N., Li C., Xu B., Xiang Y., Jia X., Yuan Z., Wu L., Zhong L., Li Y. (2021). Circular RNA Mmu_circ_0005019 Inhibits Fibrosis of Cardiac Fibroblasts and Reverses Electrical Remodeling of Cardiomyocytes. BMC Cardiovasc. Disord..

[264] Li X.X., Mu B., Li X., Bie Z. (2022). Dong CircCELF1 Inhibits Myocardial Fibrosis by Regulating the Expression of DKK2 Through FTO/M6A and MiR-636. J. Cardiovasc. Transl. Res..

[265] Zhang L., Bian Y.F., Bai R., Song X.S., Liang B., Xiao C.S. (2020). Circ_bmp2k Enhances the Regulatory Effects of Mir-455-3p on Its Target Gene Sumo1 and Thereby Inhibits the Activation of Cardiac Fibroblasts. Biochem. Cell Biol..

[266] Zheng D., Zhang Y., Hu Y., Guan J., Xu L., Xiao W., Zhong Q., Ren C., Lu J., Liang J. (2019). Long Noncoding RNA Crnde Attenuates Cardiac Fibrosis via Smad3-Crnde Negative Feedback in Diabetic Cardiomyopathy. FEBS J..

[267] Chen H., Liu J., Wang B., Li Y. (2020). Protective Effect of LncRNA CRNDE on Myocardial Cell Apoptosis in Heart Failure by Regulating HMGB1 Cytoplasm Translocation through PARP-1. Arch. Pharm. Res..

[268] Chen W., Ye Q., Dong Y. (2024). Long Term Exercise-Derived Exosomal LncRNA CRNDE Mitigates Myocardial Infarction Injury through MiR-489-3p/Nrf2 Signaling Axis. Nanomedicine.

[269] Micheletti R., Plaisance I., Abraham B.J., Sarre A., Ting C.C., Alexanian M., Maric D., Maison D., Nemir M., Young R.A. (2017). The Long Noncoding RNA Wisper Controls Cardiac Fibrosis and Remodeling. Sci. Transl. Med..

[270] Karras J., Kling F., Marcuello M., Cruz J., Tien C.-L., Roberts C., Gentry K., Watson A., Loetscher A., Rauleac T. (2024). Preclinical Evaluation of HTX-001 as a Novel Anti-Fibrotic Therapy for Non-Obstructive HCM. J. Card. Fail..

[271] Mou Y., Fan X., Sun R., Zhang X., Yu J. (2024). The Role and Mechanism of CircAMOTL1 in Hypertensive Heart Failure. J. Biomed. Nanotechnol..

[272] Chen M.S., Lee R.T., Garbern J.C. (2022). Senescence Mechanisms and Targets in the Heart. Cardiovasc. Res..

[273] Su Y., Zhu C., Wang B., Zheng H., McAlister V., Lacefield J.C., Quan D., Mele T., Greasley A., Liu K. (2021). Circular RNA Foxo3 in Cardiac Ischemia-Reperfusion Injury in Heart Transplantation: A New Regulator and Target. Am. J. Transplant..

[274] Jin L., Cai Q., Wang S., Wang S., Wang J., Quan Z. (2020). Long Noncoding RNA PVT1 Promoted Gallbladder Cancer Proliferation by Epigenetically Suppressing MiR-18b-5p via DNA Methylation. Cell Death Dis..

[275] Wang W.X., Rajeev B.W., Stromberg A.J., Ren N., Tang G., Huang Q., Rigoutsos I., Nelson P.T. (2008). The Expression of MicroRNA MiR-107 Decreases Early in Alzheimer’s Disease and May Accelerate Disease Progression through Regulation of β-Site Amyloid Precursor Protein-Cleaving Enzyme 1. J. Neurosci..

[276] Ke S., Yang Z., Yang F., Wang X., Tan J., Liao B. (2019). Long Noncoding RNA NEAT1 Aggravates Aβ-Induced Neuronal Damage by Targeting MiR-107 in Alzheimer’s Disease. Yonsei Med. J..

[277] Qu K., Zhang X., Lin T., Liu T., Wang Z., Liu S., Zhou L., Wei J., Chang H., Li K. (2017). Circulating MiRNA-21-5p as a Diagnostic Biomarker for Pancreatic Cancer: Evidence from Comprehensive MiRNA Expression Profiling Analysis and Clinical Validation. Sci. Rep..

[278] Dioguardi M., Caloro G.A., Laino L., Alovisi M., Sovereto D., Crincoli V., Aiuto R., Coccia E., Troiano G., Muzio L. (2020). Lo Circulating MiR-21 as a Potential Biomarker for the Diagnosis of Oral Cancer: A Systematic Review with Meta-Analysis. Cancers.

[279] Li Z., Zhou Y., Yang G., He S., Qiu X., Zhang L., Deng Q., Zheng F. (2019). Using Circular RNA SMARCA5 as a Potential Novel Biomarker for Hepatocellular Carcinoma. Clin. Chim. Acta.

[280] Hussein N.A., El Sewedy S.M., Zakareya M.M., Youssef E.A., Ibrahim F.A.R. (2023). Expression Status of Circ-SMARCA5, Circ-NOL10, Circ-LDLRAD3, and Circ-RHOT1 in Patients with Colorectal Cancer. Sci. Rep..

[281] Li B., Wang K., Cheng W., Fang B., Li Y.H., Yang S.M., Zhang M.H., Wang Y.H., Wang K. (2024). Recent Advances of PIWI-interacting RNA in Cardiovascular Diseases. Clin. Transl. Med..

[282] Lv L., Yuan K., Li J., Lu J., Zhao Q., Wang H., Chen Q., Dong X., Sheng S., Liu M. (2024). PiRNA CFAPIR inhibits cardiac fibrosis by regulating the muscleblind-like protein MBNL2. Biochim. Biophys. Acta Mol. Basis Dis..

[283] Gao X.Q., Zhang Y.H., Liu F., Ponnusamy M., Zhao X.M., Zhou L.Y., Zhai M., Liu C.Y., Li X.M., Wang M. (2020). The piRNA CHAPIR regulates cardiac hypertrophy by controlling METTL3-dependent N^6^-methyladenosine methylation of Parp10 mRNA. Nat. Cell Biol..

[284] Koch C., Reilly-O’Donnell B., Gutierrez R., Lucarelli C., Ng F.S., Gorelik J., Ivanov A.P., Edel J.B. (2023). Nanopore Sequencing of DNA-Barcoded Probes for Highly Multiplexed Detection of MicroRNA, Proteins and Small Biomarkers. Nat. Nanotechnol..

[285] Ren R., Cai S., Fang X., Wang X., Zhang Z., Damiani M., Hudlerova C., Rosa A., Hope J., Cook N.J. (2023). Multiplexed Detection of Viral Antigen and RNA Using Nanopore Sensing and Encoded Molecular Probes. Nat. Commun..

[286] Schubert S.M., Arendt L.M., Zhou W., Baig S., Walter S.R., Buchsbaum R.J., Kuperwasser C., Walt D.R. (2015). Ultra-Sensitive Protein Detection via Single Molecule Arrays towards Early Stage Cancer Monitoring. Sci. Rep..

[287] Takvorian K.S., Wang D., Courchesne P., Vasan R.S., Benjamin E.J., Cheng S., Larson M.G., Levy D., Ho J.E. (2023). The Association of Protein Biomarkers with Incident Heart Failure with Preserved and Reduced Ejection Fraction. Circ. Heart Fail..

[288] Vanhaverbeke M., Attard R., Bartekova M., Ben-Aicha S., Brandenburger T., De Gonzalo-Calvo D., Emanueli C., Farrugia R., Grillari J., Hackl M. (2022). Peripheral Blood RNA Biomarkers for Cardiovascular Disease from Bench to Bedside: A Position Paper from the EU-CardioRNA COST Action CA17129. Cardiovasc. Res..

